# Molecular Characterization of Calu‑3 Cells from Submerged to Air–Liquid Interface to Model Lung Infections

**DOI:** 10.1021/acs.jproteome.4c00975

**Published:** 2026-01-28

**Authors:** Deivid Martins Santos, Edmarcia Elisa de Souza, Janaina Macedo-da-Silva, Sueli Mieko Oba-Shinjo, Claudia Blanes Angeli, Vinícius de Morais Gomes, Simon Ngao Mule, Lays Adrianne Mendonça Trajano, Guilherme Antonio de Souza-Silva, Silvia Beatriz Boscardin, Edison Luiz Durigon, Ruy Gastaldoni Jaeger, Vanessa Morais Freitas, Carsten Wrenger, Martin Røssel Larsen, Livia Rosa-Fernandes, Suely Kazue Nagashi Marie, Giuseppe Palmisano

**Affiliations:** † GlycoProteomics Laboratory, Department of Parasitology, Institute of Biomedical Sciences, 28133 University of São Paulo, São Paulo 05508-000, Brazil; ‡ Unit for Drug Discovery, Department of Parasitology, Institute of Biomedical Sciences, University of São Paulo, São Paulo 05508-000, Brazil; § Cellular and Molecular Biology Laboratory, Department of Neurology, Faculty of Medicine (FMUSP), University of São Paulo, São Paulo 01246-903, Brazil; ∥ Laboratory of Antigen Targeting for Dendritic Cells, Department of Parasitology, Institute of Biomedical Sciences, University of São Paulo, São Paulo 05508-000, Brazil; ⊥ Laboratory of Clinical and Molecular Virology, Department of Microbiology, Institute of Biomedical Sciences, University of São Paulo, São Paulo 05508-000, Brazil; # Institut Pasteur of São Paulo, São Paulo 05508-020, Brazil; ¶ Department of Biochemistry and Molecular Biology, University of Southern Denmark, Campusvej 55, Odense M 5230, Denmark; ∇ Centre for Motor Neuron Disease Research, Faculty of Medicine, Health & Human Sciences, Macquarie Medical School, Sydney 2113, Australia; ○ School of Natural Sciences, Macquarie University, Sydney 2113, Australia; ⧫ Tumor Microenvironment Lab, Institute of Biomedical Sciences, University of São Paulo, São Paulo 05508-000, Brazil

**Keywords:** air–liquid interface, calu-3, polarization, proteomics, transcriptomics, tight junctions, SARS-CoV-2

## Abstract

The air–liquid interface (ALI) model using Calu-3 cells has been used to model lung diseases. In ALI, Calu-3 polarizes and changes to a mucus-producing cell. Polarized Calu-3 similarity with primary cells has been proven; however, no studies have been focusing on the pathways differentially expressed in ALI. Here, we profiled the proteome and transcriptome of Calu-3 from submerged (nonpolarized) to ALI (polarized) conditions, and in the omics data, we observed an increase in cell replication in the nonpolarized condition while polarized cells presented higher activation of cellular energy production, protein maturation and recycle, and expression of immune molecules. Moreover, the omics findings showed upregulation of different biological processes related to the protein quality control system and antigen processing presentation in polarized cells. Immunoblot and fluorescence microscopy confirmed increased expression of bronchial epithelium integrity components such as mucus and tight junctions in polarized cells and revealed a characteristic protein expression and cellular organization found in normal lung epithelium. Furthermore, SARS-CoV-2 infection in polarized cells revealed increased cell death associated with the higher expression of ACE2. The differences observed in this study give us a better understanding of how ALI can mimic human bronchial-epithelial cells and its applications in different contexts of lung diseases.

## Graphical abstract

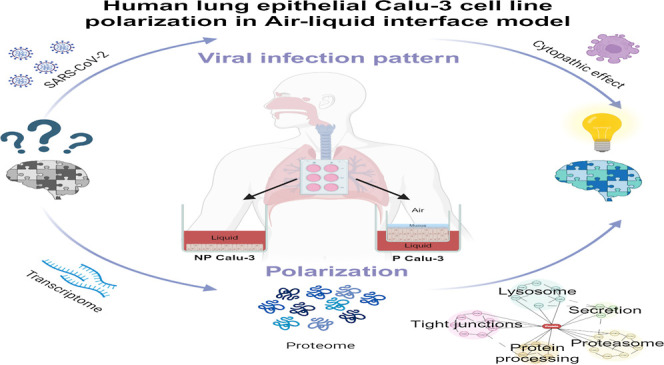

## 1 Introduction

In recent decades, lung infections have been the subject of great concern, especially those caused by coronaviruses (CoVs), a group of positive-sense single-stranded RNA viruses from the *Coronaviridae* family. The human CoVs infections usually present as a common cold; however, the emergence of zoonotic CoVs infecting humans has caused great concern in public health.^1^ The first epidemics caused by zoonotic CoVs occurred with the emergence of severe acute respiratory syndrome coronavirus (SARS-CoV) in 2002, presenting a mortality rate of 9.2%.^2^ Ten years later, in 2012, the Middle East Respiratory syndrome coronavirus (MERS-CoV) was isolated for the first time, and contrary to SARS-CoV, MERS-CoV still causes outbreaks in the Middle East in current days with a 34% mortality rate.^3^ Lastly, in December 2019, a new coronavirus emerged, the severe acute respiratory syndrome coronavirus 2 (SARS-CoV-2), presenting itself as a highly contagious virus that resulted in the COVID-19 pandemic.^4^ Due to the great epidemic and pandemic potential related to CoVs, these pathogens are on the priority list in research for the development of therapies.^5^

The need to develop new in vitro cell culture techniques has become urgent over time. This need arises from ethical questions regarding animal use in research and the low translation of knowledge from animal to human trials.^6^ On the other hand, conventional in vitro models cannot accurately reproduce the complexity of a biological system, and, even using primary cell lines, there are still challenges regarding the low rate of replication and survival of these cell types in culture. Because of all these limitations, in the drug discovery field, only less than half of all potential new drugs succeed in phase II and III tests.^7^ In the past years, advances in the development of new cell culture techniques have been proposed in different areas of research.^8–10^ For example, in 3D cultures, the cells have a format and arrangement closer to what is naturally found in the human body and present a higher degree of differentiation.^7^

The air–liquid interface (ALI) cell culture is a model in which the cell is cultivated in an insert composed of a porous membrane in which the cell is in contact with a liquid environment (culture medium) in the basal portion and in direct contact with the air in the apical portion. This model has been widely used in studies of the cytotoxicity of polluting agents, drug development, and infections, including lung infections.^11–13^ Among the cell lines that can be used in ALI, one cell that has stood out is the human lung broncho epithelial adenocarcinoma, Calu-3. Under ALI conditions, Calu-3 cells undergo from a nonpolarized state (no mucus production) to a polarized state, becoming a ciliated mucus-producing cell and forming a pseudostratified tissue.^14^

The advantages of using Calu-3 in ALI have been proven in different ways. Sanchez-Guzman et al. (2021)^14^ showed that Calu-3 cells secretome presents high proteome similarity when compared to secretome of normal human bronchial-epithelial (NHBE) primary cells. Moreover, they observed that barrier function and mucus production depend on fetal bovine serum supplementation. Mukherjee; Pritchard; Bosquillon (2012)^15^ reported that Calu-3 in ALI expresses organic cation transporters (OCT) like that observed in NHBE cells, evidencing its usefulness in studies of inhaled drug transport. Tseng et al. (2005)^12^ showed that polarization of Calu-3 induces differences in abundance and localization of membrane receptors, such as angiotensin-converting enzyme 2 (ACE-2).

Despite the advances in the characterization of the ALI model using Calu-3 cells, all of the information obtained cannot show the complete scenario. A global analysis of the changes that occur in this cell during its process of changing from submerged culture (nonpolarized, NP) to ALI (polarized, P) is still needed. This study aimed to analyze the molecular changes that occur during Calu-3 cell polarization using RNA-seq transcriptomics and MS-based proteomics approaches, the regulation of the protein quality control system through immunoblots, the expression of important components for lung epithelial integrity and host–pathogen interaction by microscopy techniques, and the impact of ALI-cultured cells on SARS-CoV-2 infection.

## 2 Materials and Methods

### 2.1 Cell Culture Conditions

Calu-3 cells were cultivated in DMEM high glucose supplemented with 20% (v/v) FBS, 1% (v/v) nonessential amino acids, 4.5 g/L glucose, 2 mM l-glutamine, 1 mM sodium pyruvate, 100 U/mL penicillin–streptomycin, and 1.5 g/L NaHCO_3_ and were kept at 37 °C with 5% CO_2_.

Nonpolarized Calu-3 cells were cultivated in submerged conditions in a 6-well plate (5 × 10^5^ cells/well) and kept in culture for 7 days. Polarized Calu-3 cells were cultivated in an insert for a 6-well plates transparent membrane (PET), pore diameter: 0.4 μm (GREINER-657641) (5 × 10^5^ cells/insert). The cells were kept in culture in the same medium as that described above. Fresh medium was replaced in the apical and basolateral compartments every 2 days. After the formation of the cell layer (7 days of culturing), the Calu-3 polarization was induced by removing the medium from the apical part. Cell layer formation in Polarized Calu-3 was evaluated by Trans-epithelial electrical resistance (TEER) measurements following the instructions previously reported.^14^ The NP Calu-3 cells were collected for analysis after 7 days of culturing (monolayer formation), and P Calu-3 cells were collected after 11 days at ALI (18 days of culturing).

### 2.2 Transcriptomics

High-throughput sequencing for transcriptomics analysis was performed similarly to the conditions previously reported.^16^ Briefly, triplicates of NP- and P-Calu-3 cells were collected in RLT Buffer (Qiagen), and total RNA was extracted using an RNeasy mini kit (Qiagen). After RNA integrity and concentration assessment, the library was constructed from 500 ng of total RNA using QuantSeq 3′ mRNA-Seq Library Prep kit FWD for Illumina (Lexogen, Vienna, Austria). Qubit dsDNA HS Assay Kit (Thermo Fisher Scientific) was used for library concentration measurement. The Agilent D1000 ScreenTape System on TapeStation 4200 (Agilent Technologies) was used to determine the size distribution. Sequencing was performed at the next-generation sequencing facility core (SELA) at the Faculty of Medicine, University of São Paulo (FMUSP), using the NextSeq 500 platform (Illumina, San Diego, CA, USA).

### 2.3 Proteomics

#### 2.3.1 Trypsin Digestion

For trypsin digestion, Calu-3 cells (nonpolarized and polarized) were collected in 8 M urea + 50 mM ammonium bicarbonate + 1 × protease/phosphatase cocktail inhibitors (Sigma-Aldrich) and then lysed by three cycles of ultrasonication on ice (30% amplitude, 15 s ON, and 15 s OFF).^17^ Protein quantification was performed using Qubit fluorometric quantitation (Thermo Fisher Scientific) following the manufacturer’s instructions. For proteomics analysis, 30 μg of proteins was reduced with dithiothreitol (DTT) to a final concentration of 10 mM for 45 min at 30 °C, alkylated with iodoacetamide (IAA) to a final concentration of 40 mM for 30 min at room temperature in the dark, and then digested with trypsin (1:50) (Promega). After 16 hs of trypsin digestion at 30 °C, the reaction was stopped by adding trifluoroacetic acid (TFA) to a final concentration of 1%. The peptides were desalted in a homemade C18 tip column, and the desalted peptides were eluted with 100 μL 50% acetonitrile diluted in 0.1% TFA, followed by 100 μL 70% acetonitrile diluted in 0.1% TFA. Eluted peptides were dried down by vacuum centrifugation and stored at −20 °C until LC–MS/MS analysis.

#### 2.3.2 Mass Spectrometry-Based Analysis

LC–MS/MS analysis was performed on an EASY-Spray PepMap 50 cm × 75 μm C18 column using an Easy nLC1000 nanoflow system (Thermo Fisher Scientific) coupled to an Orbitrap Velos mass spectrometer (Thermo Fisher Scientific). The HPLC gradient was 2–30% solvent B (*A* = 0.1% formic acid; *B* = 95% ACN, 0.1% formic acid) in 80 min at a flow of 300 nL/min. The total acquisition time was 105 min. The top 20 most intense precursors selected from the FT MS1 full scan acquired in the Orbitrap (at resolving power 60 K) were ion trap-isolated (isolation width 2) and fragmented by CID and detected in the ion trap with 35% as normalized collision energy. The MS1 scan range was between 350 and 1500 *m*/*z*, and the activation time in MS2 was 10.000 ms. A minimum intensity of 5 × 10^3^ was used as a threshold for precursor ion selection and default charge state of 2. The dynamic exclusion time is 15 s. Internal calibration was performed using the ion at *m*/*z* 445.12003.

#### 2.3.3 Protein Identification and Quantification

LC–MS/MS raw files were analyzed using the Thermo Proteome Discoverer v. 2.4.1.15 for identification and label-free quantification (LFQ) of the proteins using Sequest HT. MS/MS spectra were searched against *Homo sapiens* (SwissProt TaxID = 9606). Processing workflow was based on Spectrum files RC, Spectrum selector, Sequest HT, Percolator, and Minora feature detector nodes. The mass tolerance level was set to 10 ppm for MS and 0.6 Da for MS/MS. Enzyme specificity was set to trypsin with a maximum of two missed cleavages. Dynamic modifications were set for oxidation (M) (+15.995 Da). Dynamic modifications (protein terminus) were acetyl (+42.011 Da) (N-Terminus), Met-loss (−131.040 Da) (M), and Met-loss + acetyl (−89.030 Da) (M). Static modifications were set to carbamidomethyl (C) (+57.021 Da). The minimum peptide length was set to six amino acid residues. PSMs, peptides, and proteins were filtered to achieve a false discovery rate (FDR) < 1%. The consensus workflow was based on PSM grouper, peptide validator, peptide and protein filter, protein score, protein grouping, peptide protein annotation, and protein FDR validator. For peptide and protein quantification, the following were applied: feature mapper and precursor ions quantifier nodes. Peptide normalization was based on total peptide amount. Protein abundances were calculated based on the summed top 3 peptides abundances. Protein ration between conditions was calculated using the Pairwise ratio excluding the modified peptides. Proteins that did not present at least two unique peptides were excluded from the final protein table before performing statistical analysis. Detailed workflow parameters are shown in the Supporting Information, page S18.

### 2.4 Bioinformatics Analysis

Differentially regulated proteins were accessed using Perseus software after *t*-test statistical analysis with Benjamini–Hochberg-based FDR correction, at a cutoff FDR < 0.05. Functional enrichment analysis was performed on the STRING Database (https://string-db.org/) using only annotated genes with a significant threshold of *q*-value of 0.05 adjusted with Benjamini–Hochberg FDR method.^18^ Protein and mRNA network interaction analysis was performed using Cytoscape v.3.10.0 by selecting the *H. sapiens* species, applying full STRING network type and adjusting the confidence score cutoff to 0.4.^19^ Enriched pathways analysis was performed using the Reactome and KEGG platform (*q*-value <0.05).^20^

### 2.5 Viral Infection

All of the SARS-CoV-2 infections were performed in the BSL-3 Cell Culture Facility for Animal and Vector Research at the Department of Parasitology, Institute of Biomedical Sciences, University of São Paulo, under laboratory biosafety guidance related to coronavirus disease (COVID-19): Interim guidance, January 28, 2021 (https://www.who.int/publications/i/item/WHO-WPE-GIH-2021.1, accessed May 1, 2021).

Nonpolarized Calu-3 cells were infected after 7 days of culturing in submerged conditions. Polarized Calu-3 cells were infected in the apical region after 11 days of culturing at ALI. Both Calu-3 cells were infected with SARS-CoV-2 wild-type isolate (HIAE-02: SARS-CoV-2/SP02/human/2020/BRA, GenBank accession number MT126808) with MOI: 0.1 and mock-infected control for each condition in the serum-free medium for 1 h at 37 °C and 5% CO_2_. Following adsorption, the medium was removed, and fresh DMEM HG supplemented with 2.5% FBS was added in the well (NP cells) and in the basal region of the insert (P cells), and cells were further incubated at 37 °C and 5% CO_2_.

After 24 h of infection, mock and infected Calu-3 cells were collected. For Western blot applications, the cell pellet was collected in the 8 M urea + 50 mM AmBic + 1 × protease/phosphatase inhibitor. For immunofluorescence microscopy, the cells were washed with a PBS buffer and fixed with 4% PFA.

### 2.6 Immunoblotting

A total of 30 μg of proteins obtained from the cells was denatured in a dry bath at 99 °C for 10 min in 1 × sample buffer (50 mM Tris–HCl pH 6.8; 2% m/v SDS; 10% v/v glycerol; 5% v/v betamercaptoethanol; and 5% v/v bromophenol 0.3% m/v) and subsequently subjected to SDS-PAGE (12%). The fractionated proteins were transferred to PVDF membranes by wet transfer and blocked by incubation with 5% nonfat dry milk in TBS with 0.1% of Tween 20. The membranes were incubated overnight at 4 °C with primary antibodies, and then secondary antibody incubation was performed for 1 h at room temperature. The complete list of antibodies is described in Table S1. The membranes were developed through the SuperSignal TM West Pico PLUS Chemiluminescent substrate kit (Thermo Scientific), and the images were obtained using ChemiDoc TM Imaging System (BioRad Laboratories, CA, USA). The volume density of the chemiluminescent bands was calculated using ImageJ (National Institutes of Health) as an integrated optical density × mm^2^ after background correction in two independent experiments. The bands quantification was analyzed using GraphPad v8.0.1 software using *t*-test (*p* < 0.05) after the normality test.

### 2.7 Fluorescence Microscopy and Image Analysis

NP cells were cultivated (5 × 10^5^ cells/well) on 13 mm diameter coverslips placed into a 6-well plate and kept in standard culture conditions for 7 days, while P cells were cultivated (5 × 10^5^ cells/insert) in 6-wells plate inserts and kept in culture for 11 days at ALI. For P Calu-3 cells, all the fluorescence microscopy steps were performed inside the insert.

The cells were washed with 1 × PBS and fixed using 4% paraformaldehyde (Sigma-Aldrich, F1635) for 10 min at room temperature. Then, the cells were permeabilized with Triton X-100 0.1% diluted in 1 × PBS for 10 min and blocked with 5% BSA + 2% FBS + 0.1% Tween 20 diluted into 1 × PBS for 1 h at RT. Primary antibodies mouse antispike (MA536245; 1:200 dilution), rabbit anti-ACE2 (SAB3500346; 1:100 dilution), rabbit anti-ZO1 (61-730-0; 1:300 dilution), and Hoechst 33,342, all from Thermo Fisher Scientific, were diluted in 1 × PBS and added to NP coverslips or P inserts following overnight incubation at 4 °C. Then, the cells were washed with 1 × PBS and incubated at RT for 1 h with respective secondary antibodies antimouse Alexa Fluor 488 (A-21200; 1:500 dilution) and antirabbit Alexa Fluor 568 (A10042; 1:500), all from Thermo Fisher Scientific.

The insert membranes containing P Calu-3 were kindly excised using a scalpel and mounted on coverslips and glass slides using ProLong Gold mounting media (Thermo Fisher Scientific). Images were obtained through a series of Z stack images captured in 0.22 μm-thick sections using a ZEISS AxioObserver Z1 with ApoTome2 and a biogas incubation chamber with a ×63 oil-immersion objective. To perform comparative analysis between NP- and P-cells, fluorescence range intensities were adjusted identically during image capture. Z stack image series of the entire cell volume were projected in 2D maximum projections using ImageJ FIJI (National Institutes of Health) or rendered for 3D projections using Zen 2.6 blue software (ZEISS).

#### 2.7.1 Scanning Electron Microscopy

Polarized and nonpolarized cells were fixed in 2% (v/v) glutaraldehyde in 0.1 M cacodylate buffer, pH 7.4 overnight at 4 °C. After that, cells were rinsed in 0.2 M cacodylate buffer, followed by postfixation in 1% (w/v) osmium tetroxide for 1 h at room temperature. Cells were dehydrated using a series of increasing concentrations of ethanol, dried with Hexamethyldisilazane (HMDS, Ted Pella) for 2 min, air-dried, coated with gold in a sputter coater (Bal Tec), and examined with a Jeol JSM-IT710HR SEM microscope.

### 2.82.9 Transmission Electron Microscopy

Cells were fixed in 2% glutaraldehyde in 0.1 M cacodylate buffer, pH 7.4 overnight at 4 °C. The fixation was followed by rinsing in 0.2 M cacodylate buffer, followed by postfixation in 1% (w/v) osmium tetroxide for 1 h at room temperature. Samples were then washed in distilled water, stained in bloc with 0.5% uranyl acetate, rinsed, and dehydrated in graded ethanol. After immersion in propylene oxide, samples were embedded in epoxy resin (Spurr, Electron Microscopy Sciences, EMS, Hatfield PA, USA) and polymerized for 48 h at 75 °C. Sections (0.5 μm-thick) were stained with 1.0% toluidine blue in 1.0% aqueous sodium borate for light microscopic examination. Ultrathin sections were stained with lead citrate and uranyl acetate and examined with a TECNAI G20 transmission electron microscope at 200 kV.

### 2.9 Statistical Analysis

Statistical analyses were performed with GraphPad Prism version 8.0. Gaussian distribution and normality test were applied in all results. To obtain the statistical differences, an unpaired *t*-test with Welch’s correction and ordinary one-way ANOVA with the Tukey posthoc test was used for multiple comparisons. For all tests, statistically significant differences were defined as *p* < 0.05.

## 3 Results

### 3.1 Comparative Transcriptomic Analysis of Nonpolarized and Polarized Calu-3 Cells Reveals a Remodeling of Cellular Pathways

An RNaseq-based transcriptomics analysis was performed to study the cellular alterations in Calu-3 cells cultured in different models. To achieve that, NP cells were grown in submerged cell culture conditions for 7 days until cell monolayer formation and P cells were cultured at ALI culture conditions and collected after 11 days, as detailed in the materials and methods section and summarized in Figure 1. The days of collection for P cells were chosen based on previous studies that evaluated mucus production and secretome analysis, in which a mature secretome profile was observed after 11 days of ALI culture.^14^

**Figure 1:**
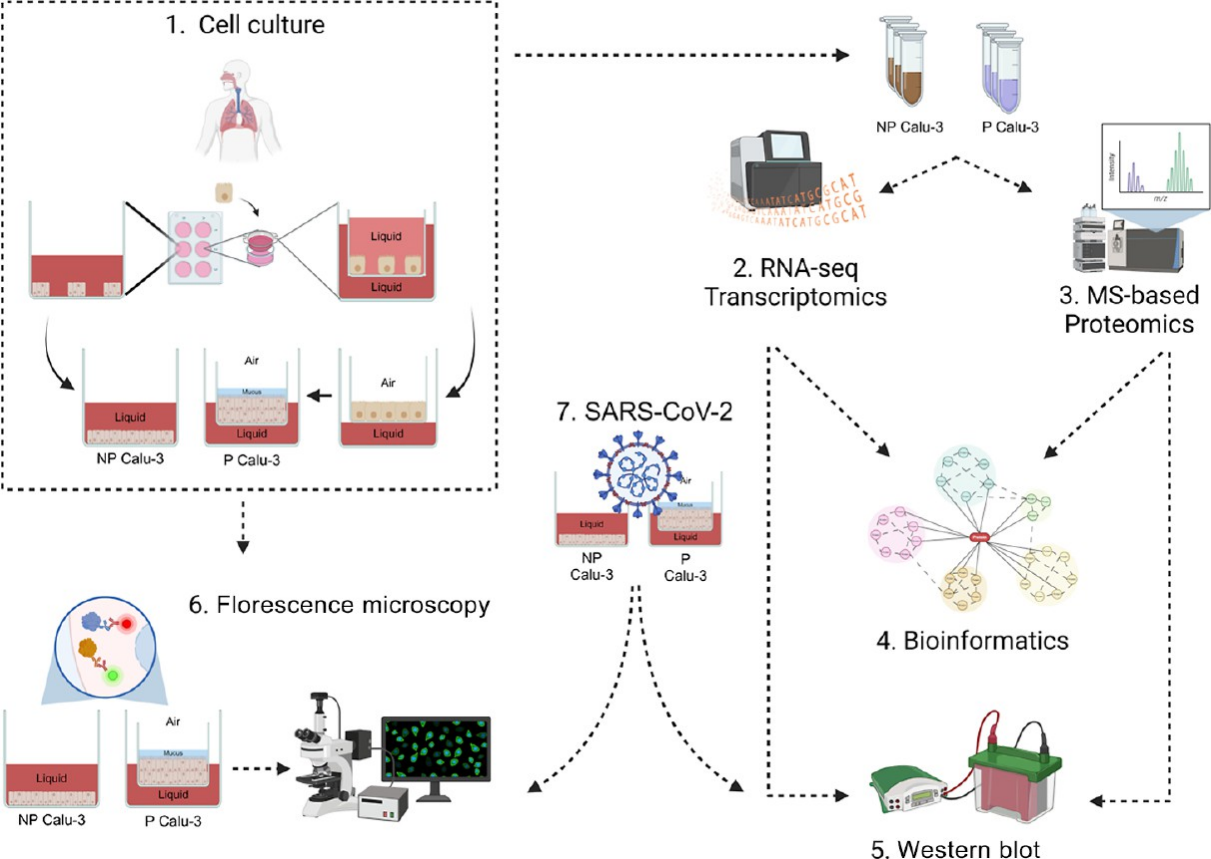
Experimental design applied to study the Calu-3 cell polarization. NP Calu-3 cells were cultured in submerged cell culture conditions and collected after monolayer formation, P Calu-3 cells were cultured in inserts for 6-well plates, and after monolayer formation, the polarization was induced by removing the medium from the apical space, and the cells were collected 11 days later (1). Omics characterization of Calu-3 cell polarization was performed using RNA-seq transcriptomics and MS-based proteomics after collecting NP and P cells with the proper lysis buffer (2,3). To access the biological processes regulated in NP and P cells, *t*-test statistical analysis using Benjamini–Hochberg FDR < 0.05 correction was applied to obtain the regulated mRNAs and proteins, followed by enrichment analysis using STRING DB, GProfiler, and Cytoscape (4). Omics data validations were performed using Western blot and microscopy techniques like immunofluorescence, scanning electron microscopy, and transmission electron microscopy (5,6). Cytopathic effect pattern and viral replication of NP and P cells were performed after 24 h of infection with SARS-CoV-2 Wuhan strain with a multiplicity of infection of 0.1 using Western blot and microscopy techniques (7). All experiments were performed in biological triplicates. Figure created in BioRender: https://BioRender.com/f05d461.

The RNaseq of NP and P cells yielded 12,259 and 12,297 genes, respectively. Principal Component Analysis separated the samples into two groups (Figure 2a), one for each culture condition, presenting a separation between them (component 1 86, 9%). Compared to NP cells, a total of 397 and 359 transcripts were down- and upregulated to P cells, respectively (Figure 2b and Table S2) and assembled into 15 clusters, with a *p*-value correction. The clusters associated with upregulated transcripts in P cells were vesicle transport, mitochondria metabolism, autophagy, antigen processing and peptide presentation, and protein degradation. On the other hand, for NP cells, the regulated transcripts were related to DNA replication, RNA splicing, cytoskeletal organization, and response to hypoxia, Figure S1. Moreover, we found two ciliary cells markers (ODF3B and TUBB4B) and three microvilli-associated proteins (SLC9A3R1, NPC1L1, and VIL1) upregulated in P cells.

**Figure 2:**
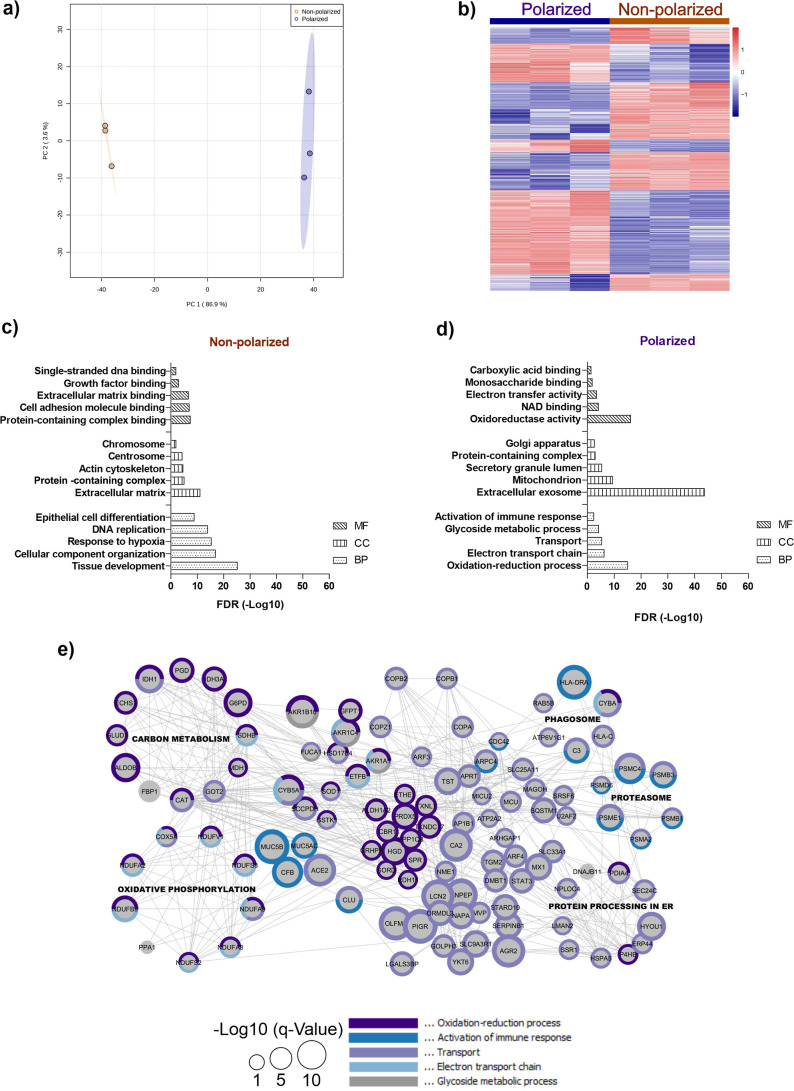
RNA-seq transcriptome analysis of nonpolarized compared to polarized Calu-3 cells. (a) Principal component analysis based on the transcript abundance of NP and P Calu-3 cells. (b) Heatmap of the NP and P Calu-3 cell-regulated genes, in which the up- and downregulated genes are represented by the colors red and blue, respectively. Regulated genes were accessed using *t*-test with Benjamini–Hochberg FDR < 0.05 correction. The clusters on the heatmap were obtained after Pearson test. Enrichment analysis of the upregulated transcripts of NP (c) and P (d) Calu-3 cells was stratified in three categories: biological process (BP), cellular component (CC), and molecular function (MF), using only annotated genes with Benjamini–Hochberg FDR < 0.05 correction. Gene network interaction analysis of upregulated genes in P Calu-3 (e) cells was plotted using Cytoscape. The rings surrounding the genes correspond to the biological processes highlighted in graph d. Node sizes correspond to the gene *q*-Value (−Log10). Highlighted gene clusters (black) in the network represent the activated KEEG pathways.

To get an overview about the biological processes activated in Calu-3 cells cultured in different models, we performed a gene ontology (GO) analysis using the regulated genes, Figure 2c,d and Table S2. Functional enrichment analysis of the upregulated transcripts in NP cells (Figure 2c) showed increased cell growth. Moreover, most of the cellular compartments of NP Calu-3 cells corresponded to cell replication like chromosome, centrosome, and actin cytoskeleton. The gene network interaction analysis also showed the KEGG Pathway enriched for cell cycle, and most of the genes were related to cell replication (Figure S2a).

Transcripts upregulated in P cells were enriched in electron transport chain, vesicle transport, and immune response activity processes (Figure 2d). The enriched KEGG pathways including these transcripts were related to cell energy metabolism like carbon metabolism and oxidative phosphorylation and proteostasis like protein processing in ER, proteasome, and phagosome (Figure 2e).

### 3.2 Protein Regulation during Calu-3 Cells Polarization

Quantitative shotgun MS-based proteomics analysis of NP and P cells was performed and compared to the transcriptomics results. In total, 1540 proteins were identified, and the PCA analysis showed two groups (Component 1, 80.6%) (Figure 3a) as observed for the RNA-seq data (Figure 2a). Compared to NP cells, a total of 349 and 398 were downregulated and upregulated for P cells, respectively (Figure 3b and Table S2). Regarding ciliary cells markers, proteomics data showed only the upregulation of TUBB4B in P cells, partially corroborating with transcriptomics findings.

**Figure 3:**
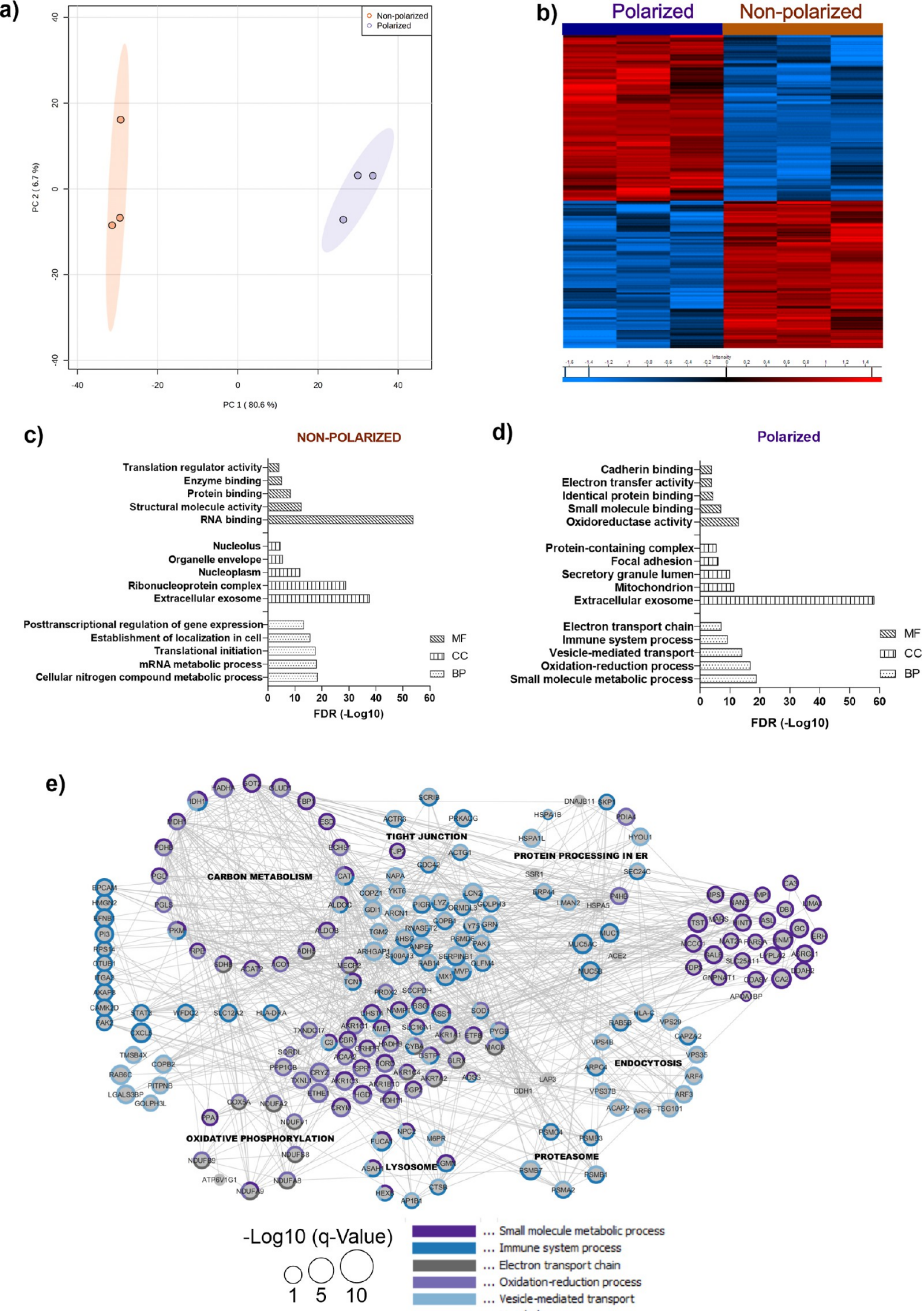
Proteome modulation of nonpolarized and polarized Calu-3 cells. (a) Principal component analysis of the identified proteins. (b) Heatmap of the NP and P Calu-3 cell-regulated proteins, in which colors red and blue represent upregulated and downregulated proteins, respectively. Regulated proteins were accessed using *t*-test with Benjamini–Hochberg FDR < 0.05 correction. Enrichment analysis of the upregulated proteins of NP (c) and P (d) Calu-3 cells were divided into three categories: biological process (BP), cellular component (CC), and molecular function (MF). Enrichment analyses were performed using only annotated proteins with Benjamini–Hochberg FDR < 0.05 correction. Protein network interaction analysis of P Calu-3 (e) cell-regulated proteins was performed using Cytoscape. The nodes represent each upregulated protein. The rings surrounding the proteins correspond to the biological processes highlighted in graph c,e for NP and P Calu-3, respectively. Highlighted gene clusters (black) in the network represent the activated KEEG pathways. Node sizes correspond to the *q*-Value (−Log10).

Proteins upregulated in NP cells were related to cell cycle and proliferation processes such as mRNA metabolic process, translational initiation, establishment of localization in cell, and posttranscriptional regulation of gene expression (Figure 3c). Protein–protein interaction network analysis revealed enrichment of cell cycle, ribosome, RNA transport, and purine metabolism, confirming an enrichment of processes associated with cell proliferation as detected in the RNA-seq analysis (Figure S2b).

Proteins upregulated in P cells were related to cadherin binding, redox, and electron transport chain processes. Focal adhesion, mitochondria, and extracellular vesicles were enriched cellular components (Figure 3d and Table S2). Protein–protein interaction analysis was related to cell energy, protein metabolism, tight junction, and protein processing in ER, lysosome, and proteasome (Figure 3e). Moreover, enrichment analysis showed in both omics data the regulation of the immune-related process in P cells and the regulated proteins are highlighted in the network analysis (Figure 4), in which there were molecules regulated only in the transcriptomics (blue) or proteomics (gray) data but also proteins that were found regulated in both omics data (purple), like components from the complement system (C3 and CFB), mucus (MUC1, MUC5AC, and MUC5B) antigen processing presentation complex classes I and II (HLA-DR, HLA-C, LY75, and ANPEP), response to infection (STAT1, RNASET2, LYZ, SERPINB1, MX1, LCN2, and DMBT1), and immune cells recruitment molecules (CHST4, CXCL5, and STAT3). Transcriptomic and proteomics data revealed that NP cells present an upregulation of processes related to cell cycle and proliferation, being most of the regulated proteins enriched in the nuclear localization, while P cells present an upregulation of processes related to tight junction and protein quality control, being most of the regulated proteins enriched in ER and extracellular localization.

**Figure 4:**
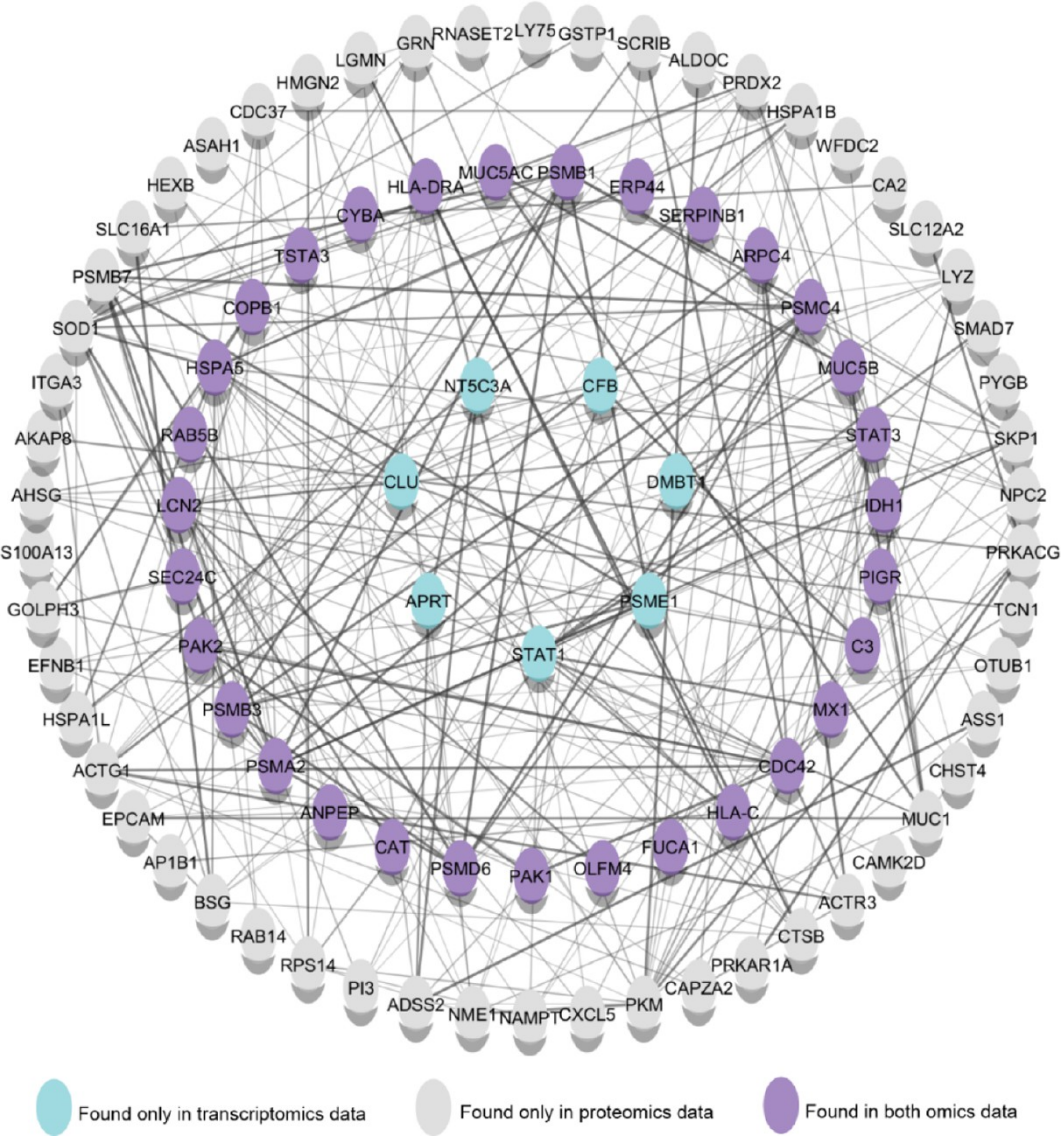
Upregulated components of biological processes related to the immunological system in polarized Calu-3 cells cultured in the air–liquid interface model. The network interaction analysis was performed on Cytoscape using the data obtained from enrichment analysis (Supporting Information S1) of polarized Calu-3 cells. Blue, gray, and purple nodes represent the upregulated proteins found only in transcriptomics data, only in proteomics data, and in both omics data. Regulated mRNA and proteins were accessed after *t*-test analysis with Benjamini–Hochberg FDR < 0.05 correction. Enrichment analysis of upregulated mRNA and proteins on STRING DB using only annotated genes.

Protein quality control is increased in Polarized Calu-3 cells cultured under ALI conditions.

Transcriptomic and proteomic analyses revealed an enrichment of protein quality control and the ER in P cells. Due to that, we monitored the levels of HSP70, BiP, and PDI, chaperones found in the ER.^21^ Higher levels of HSP70, BiP, and PDI were detected in P cells as identified in the omics analyses (Figure 5a), indicating an increased protein folding activity in P cells.

**Figure 5:**
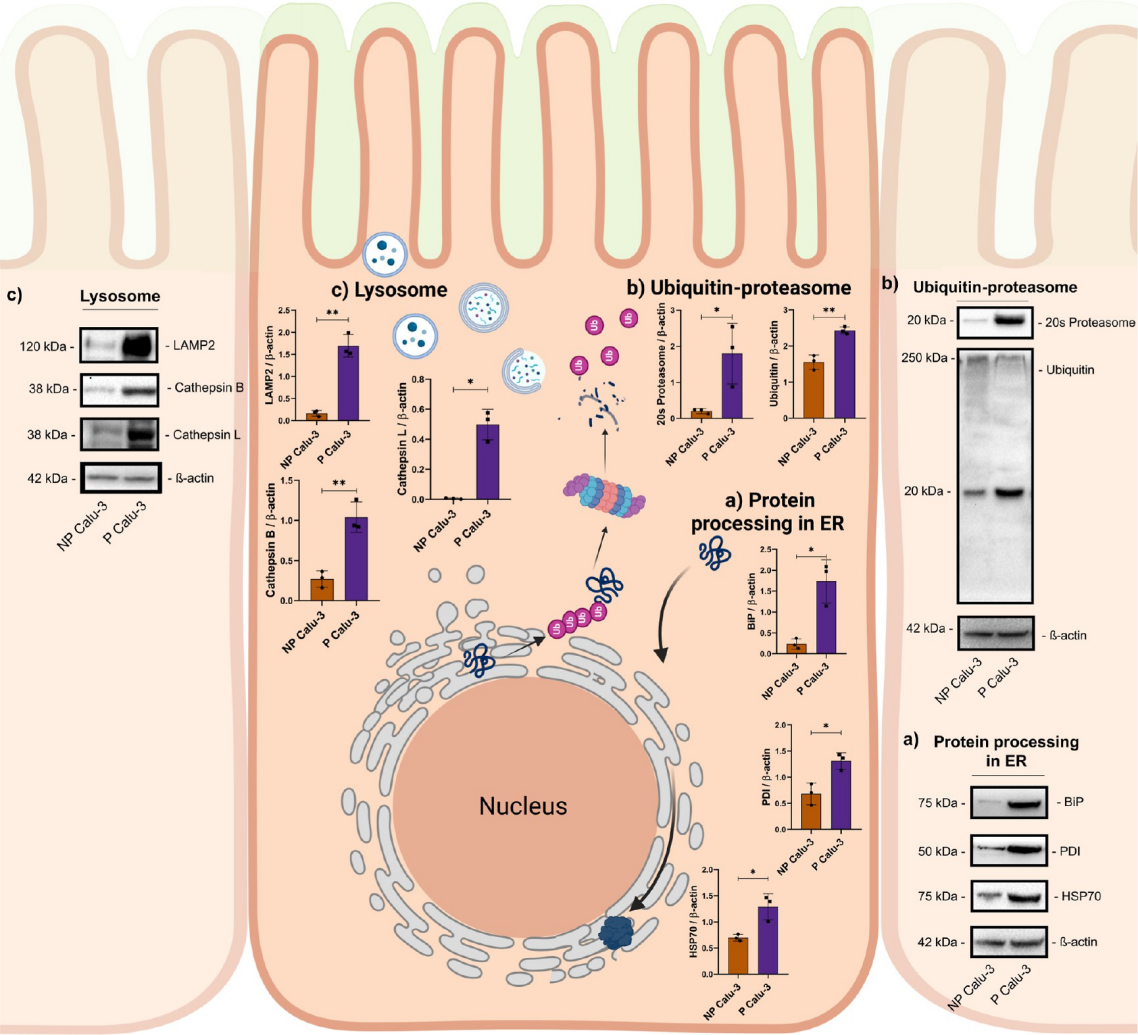
Protein quality control system in nonpolarized and polarized Calu-3 cells. Western blotting of Protein processing in endoplasmic reticulum (a), ubiquitin-proteasome (b), and lysosome (c) biological process in nonpolarized and polarized Calu-3 cells and their respective protein levels quantification and representative blot membranes. Each dot represents a replicate. The asterisk symbol (*) represents the statistical differences (****p < 0.0001; ***p < 0.001; **p < 0.005; and *p < 0.05). All western blots were performed in two independent assays (assay 2—Figure S5).

The ubiquitin-proteasome pathway (UPP) is an intracellular pathway responsible for the degradation, recycling, and subcellular redirection of proteins.^22^ Transcriptome (Figure 2e) and proteome (Figure 3e) analyses showed an upregulation of proteasomes in P cells and were corroborated by Western blot analysis with increased levels of ubiquitinated proteins and 20S proteasome (Figure 5b). In addition to the UPP, the lysosome-proteasome pathway also makes up the intracellular proteolytic pathway.^23^ Lysosome-associated membrane protein 2 (LAMP2) and Cathepsin B and L were upregulated in P cells (Figure 5c). These results showed that both Proteasome pathways (ubiquitin-dependent and lysosomal-dependent) were activated in P cells.

### 3.3 Tight Junction Expression Profile Changes in Polarized Calu-3 Cultured in ALI

Tight junctions (TJ) are intercellular connections that promote a physical barrier in the intercellular regions (Figure 6a), exerting a barrier function for the entry of foreign bodies into the innermost regions of the epithelium and participating in communication between cells; moreover, Zonula occludens 1 (ZO-1) protein was the first TJ family member discovered.^24^ In our analysis, the TJ were found upregulated in P cells in the proteomics data (Figure 3e) and validated by Western blot and immunofluorescence microscopy (Figure 6b,c), corroborating the increased TEER values in Calu-3 cells after ALI culture (Figure S3). NP cells showed a dispersed ZO-1 staining in the cytoplasm, while P cells showed a higher staining in the intercellular region. Interestingly, through 3D image projection, it could be observed that P cells expressed ZO-1 only in the apical region (Figure 6d). These results showed that Calu-3 cell polarization at ALI induced changes in ZO-1 expression in protein abundance and cellular localization.

**Figure 6:**
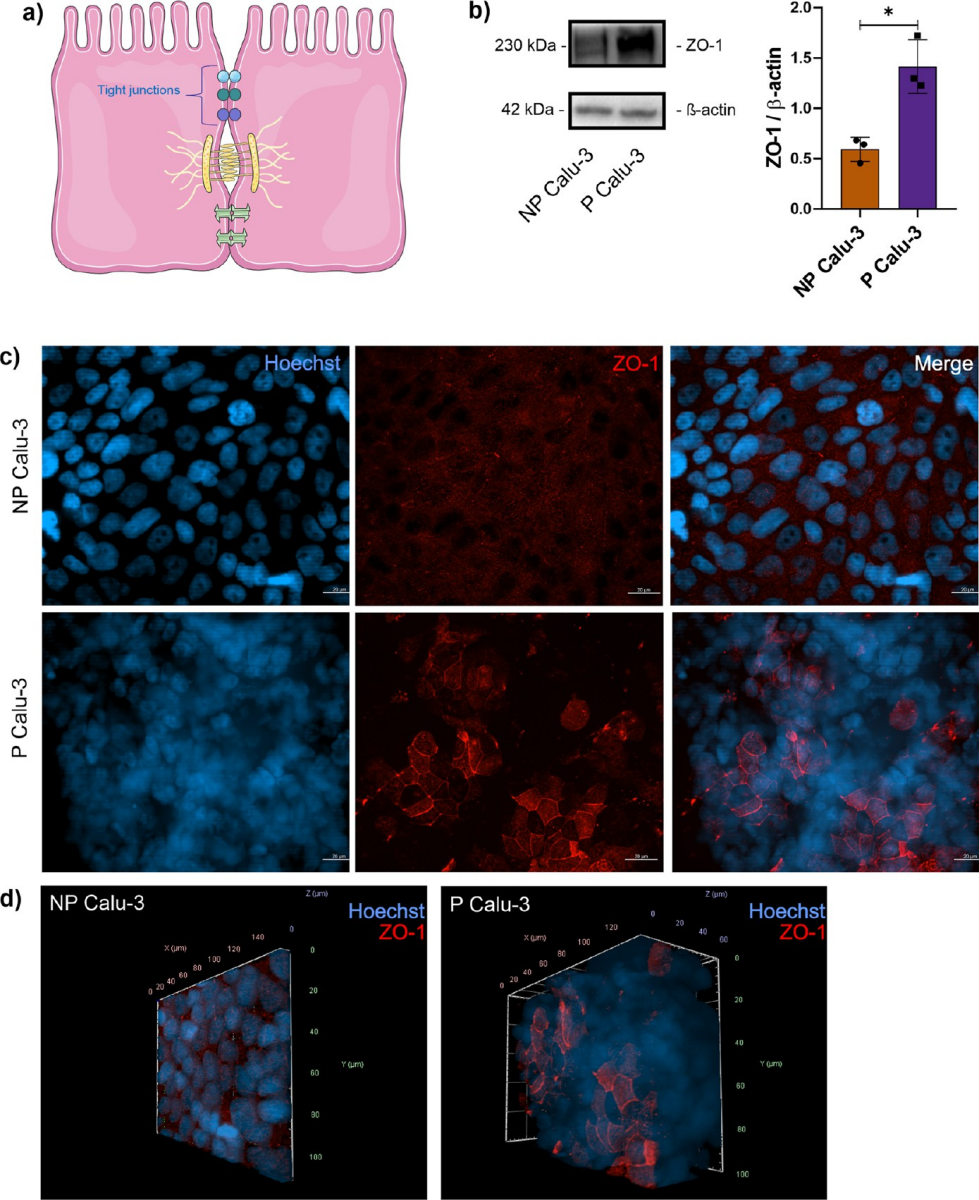
Tight junction protein expression in nonpolarized and polarized Calu-3 cells. (a) Schematic tight junctions’ expression in epithelial cells. (b) ZO-1 protein level in nonpolarized and polarized Calu-3 cells. Representative fluorescence microscopy of ZO-1 (red) and DNA by Hoechst (blue) in nonpolarized and polarized Calu-3 cells in 2D (c) and 3D (d) projections (images are representative of two independent experiments). Western blot protein levels’ statistical differences were analyzed using *t*-test with Welch’s correction. Each dot represents a replicate. The asterisk symbol (*) represents the statistical differences (****p < 0.0001; ***p < 0.001; **p < 0.005; and *p < 0.05). All Western blots were performed in two independent assays (assay 2—Figure S5).

### 3.4 Calu-3 Cell Morphological Reshape Is Induced by Polarization

SEM analysis of NP cells showed that Calu-3 cells were flat when cultured as monolayers, while P cells are dome shaped (Figure 7a–d). In higher magnification, P cells displayed a large number of microvilli at the cell surface compared to NP cells (Figure 7e,f), corroborating with the transcriptomics findings. Interestingly, although we found cilium protein markers in our omics data, electron microscopy showed only microvilli structures, and this is going to be discussed later.

**Figure 7:**
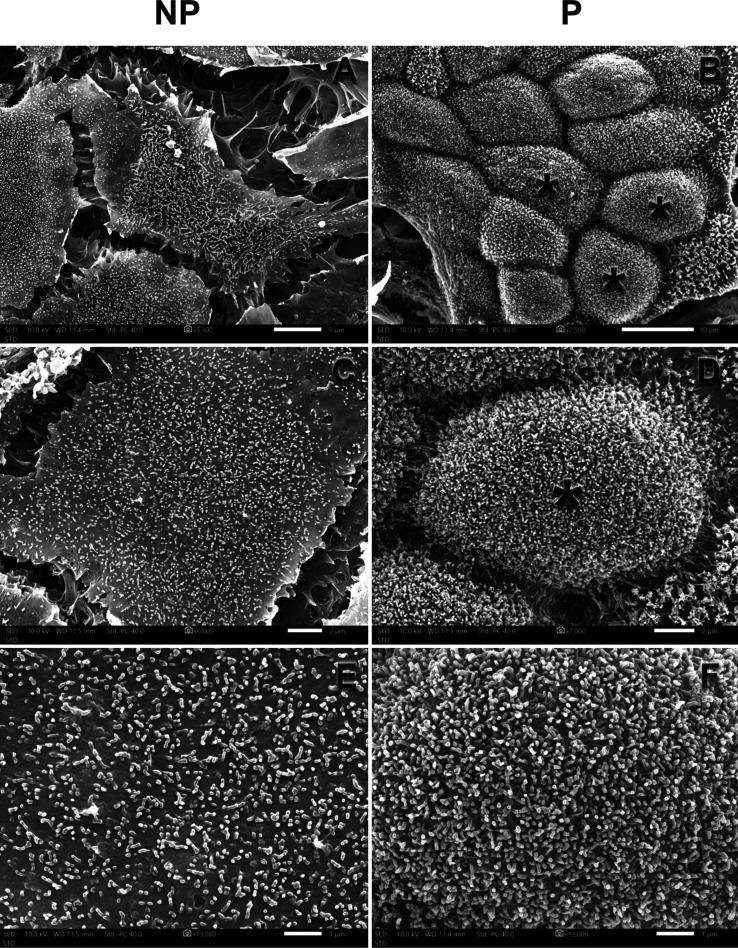
Nonpolarized and polarized Calu-3 cells morphology analysis by scanning electron microscopy. NP cells are flat (a), while P cells are dome shaped (b,c, asterisks). Higher magnification shows a profuse microvilli-like structure in P cells (d,f) compared to NP cells (c,e).

The ultrastructure of Calu-3 cells showed a loose cell–cell junction in NP when compared to P cells (Figure 7a,b). Both NP and P cells presented classical microvilli (finger-like structures formed by projections of plasma membrane sustained by the actin cytoskeleton) but in different abundance and shape. TEM and SEM analyses show microvilli more numerous and more elongated in P cells (Figures 7e,f and 8c,d, arrow). Actin terminal web was observed in both groups (Figure 8c,d). P cells exhibit cell–cell junctions with higher extension of the juxtaposed neighbor cell membrane, desmosome, and membrane fusion suggestive of TJ’ presence (Figure 8f), corroborating with proteomics data (Figure 3e), Western blot (Figure 6b), and fluorescence microscopy (Figure 6c,d). Moreover, TEM analysis showed abundant mucus secretory granules in P cells (Figure 8b,d, arrowheads), which were not observed in NP cells (Figure 8a,c). These findings corroborate with transcriptomics (Figure 2) and proteomics (Figure 3) analyses, where enrichment analysis showed that secretory granule lumen, vesicle-mediated transport, and mucus were increased in P cells.

**Figure 8:**
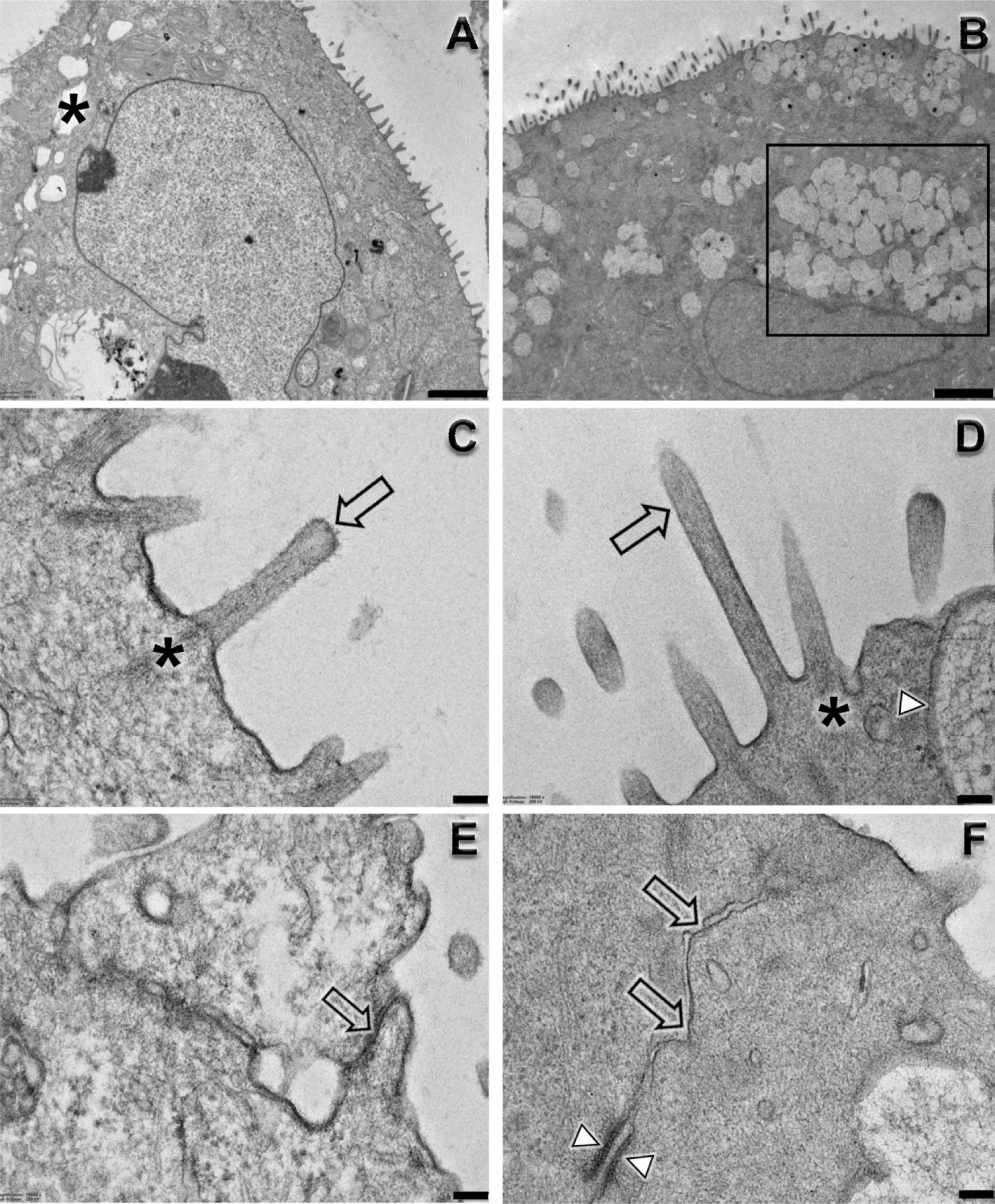
Ultrastructure of NP and P cells. NP cells show a loose cell–cell junction (a,c, asterisk) and an apical membrane with projections. NP cells are devoid of secretory granules in the cytoplasm. Polarized cells show a large amount of mucus secretory granules ((b), boxed and (d), arrowhead) and elongated microvilli ((d), arrow). Moreover, P cells are in close proximity to the neighbor cells, showing desmosomes ((f), arrowheads) and membrane fusion suggestive of tight junctions ((f), arrows). Nonpolarized cells also exhibit scatter areas of membrane fusion suggestive of tight junctions ((e), arrow). Scale bars: (A,B): 2 μm, C–F: 100 nm.

### 3.5 Polarization Makes Calu-3 Cells More Susceptible toward SARS-CoV-2 Due to Differential Expression of Viral Receptors

Angiotensin-converting enzyme 2 (ACE2) is a host surface receptor used by SARS-CoV-2, for viral adhesion and internalization.^25^ ACE2 expression was increased in P cells at the mRNA (Figure 2e) and protein (Figure 3e) levels. This was confirmed by Western blot analysis (Figure 9a,b).

**Figure 9:**
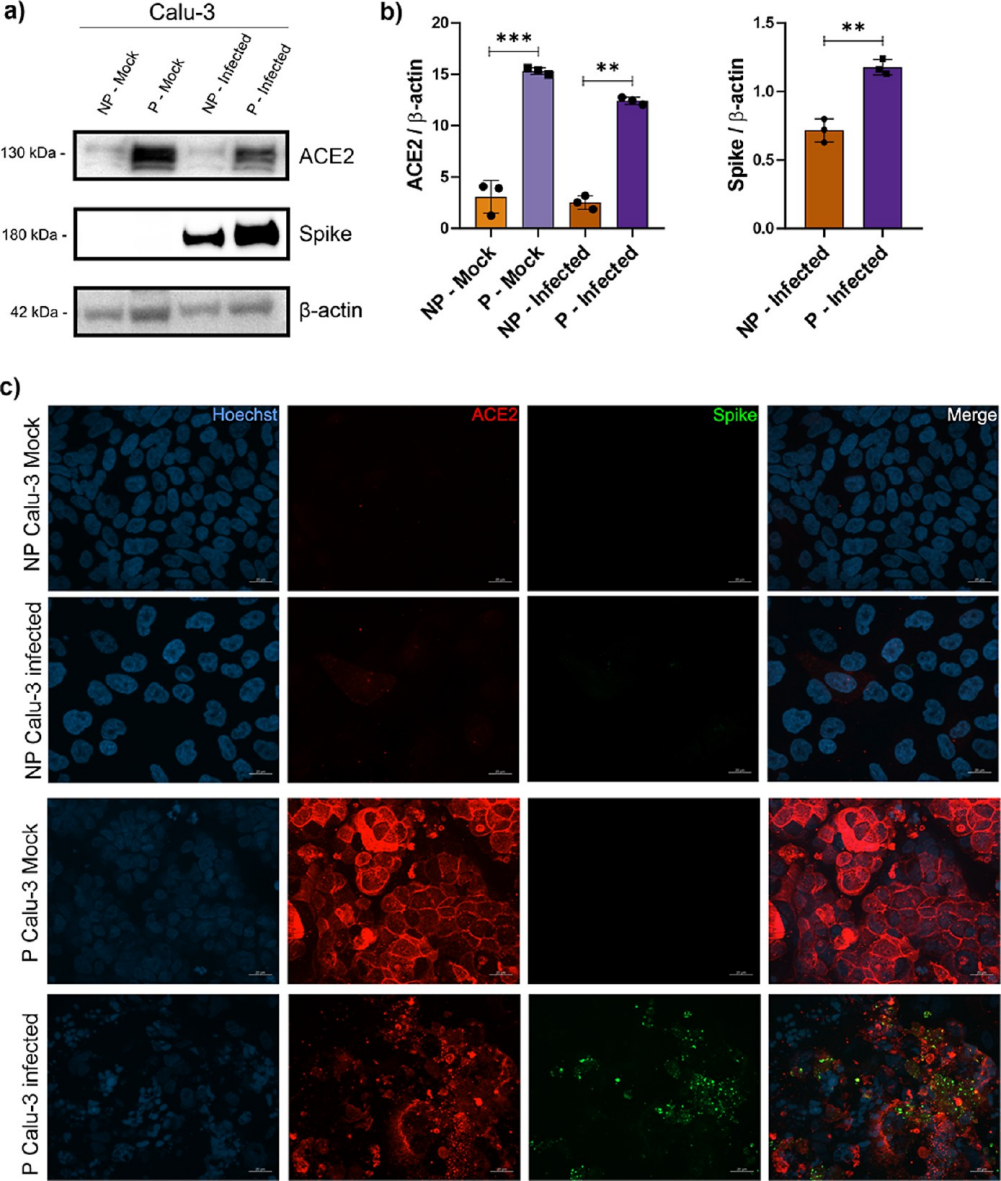
SARS-CoV-2 infection profile in nonpolarized and polarized Calu-3 cells. Expression of ACE2 and SARS-CoV-2 spike protein in infected NP and P Calu-3 cells and their corresponding mock control by representative Western blot (a) and its protein levels (b) and fluorescence microscopy (c). ACE2 = red; spike = green; and DNA = blue. Statistical differences were analyzed using *t*-test with Welch’s correction and One-way ANOVA with the Tukey posthoc test for multiple comparisons. Each dot represents a replicate. The asterisk symbol (*) represents the statistical differences (****p < 0.0001; ***p < 0.001; **p < 0.005; and *p < 0.05). All Western blots were performed in two independent assays (assay 2—Figure S5).

To analyze the effect of cell polarization on viral infection, NP and P cells were infected with Wuhan SARS-CoV-2 strain at MOI: 0.1, and the viral protein levels and cytopathic effect were evaluated after 24 h of infection. Increased levels of spike protein were found in P cells which correlated with increased ACE2 protein levels in P infected cells by Western blotting analysis (Figure 9a,b). Consistently, increased staining of ACE2 and spike proteins were detected by immunofluorescence microscopy in P cells, leading to a higher cytopathic effect (Figures 9c and S4). These results showed that the Polarized Calu-3 cells cultured under ALI conditions present increased ACE2 expression, making these cells more susceptive to SARS-CoV-2 infection.

## 4 Discussion

In this study, we used transcriptomic and proteomic approaches to analyze the changes triggered in Calu-3 cells cultured under submerged or ALI conditions. P cells increase the levels of proteins related to energy production and protein processing. Furthermore, we observed increased expression of mucins, TJ, autophagy, and components of the immune response that together are known to be essential processes for the integrity of bronchial-epithelial tissue.^26^

Morphologically, P cells cultured in ALI conditions grow forming a pseudostratified tissue, as seen in our fluorescence microscopy images and in previous work through histological analysis,^27–30^ which is the cellular arrangement characteristic of human lung epithelium. Furthermore, electron microscopy analysis showed that Calu-3 cells go through extensive morphological reshape, changing from a flat shape cell with a low number of microvilli to a dome-shaped cell with a higher number of elongated microvilli and mucus secretory granules. Calu-3 is described as a cell line originating from glands in the submucosa of human bronchi, a region responsible for the secretion of mucus and other immunologically active substances.^26^ Previous analyses of the secretome from polarized Calu-3 and the NHBE primary cells, both grown in ALI, showed the secretion of molecules related to the immune system, reporting that the P cells secretome is highly like a primary cell one.^14^ Although the immunological response is not part of the scope of this study, biological processes related to the immune system were upregulated in P cells in both omics data, such as antigen presentation complex class I (HLA-C) and class II (HLA-DRA) and related biological process like the proteasome pathways, components from the complement system, and molecules with antimicrobial activity. The human leukocyte antigens I (HLA) compose the molecules HLA-A, -B, and -C and are expressed on the surface of most mammalian cells. The HLA II are subdivided into HLA-DR, -DQ, and -DP, and, although these molecules are mainly expressed by immune cells, they can also be expressed by airway epithelial cells.^31^ Moreover, other molecules that compose mucosal immune surveillance were upregulated in P cells like CXCL5 and STAT3; these are important signals of lung epithelial cells in response to lung infection, with some functions such as recruitment of innate immune cells and production of TNF-α and IFN-γ, respectively.^32,33^

The hallmark of P cells in ALI is mucus secretion. It is noteworthy that the presence of mucus secretory granules in P cells is observed by TEM. Sanchez-Guzman et al. (2020)^14^ reported through proteomic analysis the regulation of MUC5AC, which was also identified in our P cells data, and we also detected the upregulation of MUC5B. Finally, MUC1 was upregulated in the NHBE secretome^14^ and was also found on P cells but only in our proteome data. MUC5AC, MUC5B, and MUC1 are oligomeric mucus gel forming proteins, acting as a two-edges sword. Although mucus production in normal physiological conditions is crucial for lung epithelial homeostasis and composes the mucosal immune response,^34^ studies show that dysregulation in mucus synthesis is related to the pathogenesis of several lung infections. In SARS-CoV-2-infected patients, the virus induces an overexpression of MUC1 and MUC5AC that leads to respiratory distress and are directly correlated with COVID-19 illness.^35^

The lung epithelium is a region that is constantly in contact with the external environment, requiring the resident cells to develop a barrier function to prevent foreign bodies from entering the innermost layers.^36^ To this end, lung epithelial cells express proteins that make up TJ, a group of proteins formed by claudins, tight junction-associated MARVEL protein family, and immunoglobulin-like proteins.^36^ In this study, the proteome, Western blot, and fluorescence microscopy analysis showed an upregulation of ZO-1 in P cells cultured in ALI conditions for 11 days. These findings are corroborated by TEM images, showing that P cells present plasma membranes in closer proximity compared to NP cells. In previous studies, time-course fluorescence microscopy showed that ZO-1 expression increases over time, presenting the most intense tight junction staining after 14 days.^37^ Interestingly, through a 3D projection of fluorescence microscopy images, we showed that the expression of TJ in P cells occurs only in the external layer of the apical region of the cell culture, corroborating the profile expression described previously in normal tissue.^38^ Even with all of these findings, there are still some gaps of knowledge regarding Calu-3 morphology. For example, while there are studies that report cilia expression by Calu-3,^30^ there are others that state that these cells present only microvilli structures.^29^ In our study, although we found proteins related to cilia in our transcriptomic and proteomic data, both transmission and SEM analyses showed only microvilli. The difference between P and NP cells was the higher density and the size of the microvilli structures in P cells.

The activation of the lysosomal processes, proteasome, and ER protein processing pathways was detected in P cells, indicating an increased activity of the protein quality control system, which is responsible for the proteostasis of a cell and many other processes like antigen processing presentation. Moreover, chaperones are involved in protein quality control.^39^ We observed an upregulation of PDI, HSP70, and BiP. PDI is an enzyme responsible for catalyzing the reaction that forms the disulfide bonds of polypeptide chains.^40^ HSP70 is one of the main chaperones responsible for the binding and refolding of misfolded proteins.^41^ BiP is a chaperone that belongs to the HSP70 protein family and can be found on the ER and on the cell surface membrane, where it is used as a coreceptor for MERS-CoV and SARS-CoV-2 entry^42,43^ and influences the viral replication in infections caused by SAR-CoV-2. These results showed that P cells have higher levels of chaperones, which are important during viral infections and, together with the increased expression of ACE2, that may explain the increased SARS-CoV-2 spike protein level observed in the immunoblot and microscopy in the infected P cells.

Calu-3 cells have widely been used in studies related to CoVs, mainly SARS-CoV and SARS-CoV-2, considering that this cell line expresses ACE2, the main receptor of these CoVs.^12,44^ Corroborating previous studies,^27,45^ we observed in transcriptomic, proteomic, Western blot, and IF microscopy analyses an increase in ACE2 expression in P cells. Due to that, an increased SARS-CoV-2 infection was observed in polarized cells with higher cytopathic effect.^45^ Previous studies have reported that 30% of Calu-3 (nonpolarized) express ACE2.^12^ In this study, we report a higher number of Calu-3 cells positive to ACE2 in the polarized condition compared to the nonpolarized one, showing that polarization induces broader expression of ACE2 associated with higher susceptibility to SARS-CoV-2 infection. Although ACE2 has been considered as the main receptor for SARS-CoV-2 infection,^12^ we demonstrated the increased levels of other proteins like BiP and the lysosomal proteases Cathepsins B/L, which could contribute to higher permittivity in P cells. Moreover, we observed in SEM analysis an increased number and elongated shape of microvilli in P cells. On this regard, Wu et al. (2023)^46^ demonstrated using human nasal epithelial cells cultured in ALI that although microvilli do not present ACE2 and TMPRSS2 receptors, these structures are essential for SARS-CoV-2 egress and spread, enabling the viral particles to egress the peri-ciliary layer and transport through the mucus layer.

This study elucidated the molecular changes during polarization and highlighted the use of polarized Calu-3 cells as a model to study other lung diseases, making it a viable and reliable alternative to primary cells.

## 5 Conclusion

This study highlighted transcriptome and proteome remodeling during polarization of Calu-3 cells grown in ALI conditions. The morphological changes, like growing in multilayers, were sustained by the increased expression of mucins, TJ, and processes related to proteostasis, which are components that are crucial to evaluate the lung epithelium physiology and the pathogenesis of different diseases. Increased activation of antigen presentation complexes I and II together with other immunologically active molecules indicated an immune surveillance activity in polarized Calu-3 cells, which corroborates with the normal epithelial cells that compose the mucosal immune response. Moreover, the increased expression of membrane structures, like microvilli, and ACE2 receptors enables these polarized cells to be used in investigations focused on lung viral infections, mainly those caused by SARS-CoV-2. These findings aligned with the bronchial-epithelial cell phenotype, indicating that the polarized Calu-3 cells model can mimic the lung epithelium.

## Supplementary Material







## Data Availability

The mass spectrometry proteomics data have been deposited to the PRIDE Archive (http://www.ebi.ac.uk/pride/archive/) via the PRIDE partner repository with the data set identifier PXD056869.

## Notes

The following Supporting Information is available free of charge at ACS Web site The Supporting Information is available free of charge at https://pubs.acs.org/doi/10.1021/acs.jproteome.4c00975.

List of antibodies used for Western blot and fluorescence microscopy analysis (XLSX)
List of the regulated genes and proteins for nonpolarized and polarized Calu-3 cells and their corresponding gene ontology analysis (XLSX)
Gene clusters regulation in nonpolarized and polarized Calu-3 cells; network interaction analysis of nonpolarized Calu-3 cells; evolution of Trans-epithelial electrical resistance (TEER) in polarized Calu-3 cells cultured in the air–liquid interface; cytopathic effects in nonpolarized and polarized Calu-3 cells infected with SARS-CoV-2; Western blot assays of nonpolarized and polarized Calu-3 cells; entire Western blot membranes from assay 1 and 2; and Proteome Discoverer workflows used for protein identification and quantification (PDF)
