# Supplementary material for: Molecular Characterization of Calu‑3 Cells from Submerged to Air–Liquid Interface to Model Lung Infections
Source: J Proteome Res. 2026 Jan 28;25(2):562–77. doi: 10.1021/acs.jproteome.4c00975 (PMC12888003; doi:10.1021/acs.jproteome.4c00975)
Supplement: Supplementary file 3 [file pr4c00975_si_003.pdf]

Suppementary material for

Molecular characterization of Calu-3 cells from submerged to Air-liquid interface to model lung infections.

Deivid Martins Santos[1], Edmárcia Elisa de Souza[2], Janaina Macedo-da-Silva[1], Sueli Mieko Oba-Shinjo[3], Claudia Angeli Blanes[1], Vinícius de Morais Gomes[1], Simon Ngao Mule[1], Lays Adrianne Mendonça Trajano[1], Guilherme Antonio de Souza-Silva[4], Silvia Beatriz Boscardin[4], Edison Luiz Durigon[5,6], Ruy Gastaldoni Jaeger[10], Vanessa Morais Freitas[10], Carsten Wrenger[2,6], Martin Røssel Larsen[7], Livia Rosa-Fernandes[1,8, *], Suely Kazue Nagahashi Marie[3, *], Giuseppe Palmisano[1,9, *].

1. GlycoProteomics Laboratory, Department of Parasitology, Institute of Biomedical Sciences, University of São Paulo, Brazil

2. Unit for Drug Discovery, Department of Parasitology, Institute of Biomedical Sciences, University of São Paulo, Brazil

3. Cellular and Molecular Biology Laboratory, Department of Neurology, Faculty of Medicine (FMUSP), University of São Paulo, São Paulo, Brazil

4. Laboratory of Antigen Targeting for Dendritic Cells, Department of Parasitology, Institute of Biomedical Sciences, University of São Paulo, Brazil

5. Laboratory of Clinical and Molecular Virology, Department of Microbiology, Institute of Biomedical Sciences, University of São Paulo, Brazil

6. Institut Pasteur of São Paulo, São Paulo, Brazil

7. Department of Biochemistry and Molecular Biology, University of Southern Denmark, Campusvej 55, 5230 Odense M, Denmark

8. Centre for Motor Neuron Disease Research, Faculty of Medicine, Health & Human Sciences, Macquarie Medical School, Sydney, Australia

9. School of Natural Sciences, Macquarie University, Sydney, Australia

10. Tumor Microenvironment Lab, Institute of Biomedical Sciences, University of São Paulo, Brazil

* Correspondence to:

Dr. Livia Rosa-Fernandes, liviarosa.f@gmail.com

Prof. Dr. Suely K. N. Marie, sknmarie@usp.br

Prof. Dr. Giuseppe Palmisano, palmisano.gp@gmail.com

Table of Contents

Figure S1. Gene clusters regulation in Non-polarized and Polarized Calu-3 cells S6

Figure S2. Network interaction analysis of Non-polarized Calu-3 cells S7

Figure S3. Evolution of Trans-epithelial electrical resistance (TEER) in polarized Calu-3 cells cultured in Air-liquid Interface S8

Figure S4. Cytopathic effect in Non-polarized and Polarized Calu-3 cells infected with SARS-CoV-2 .... S8

Figure S5. Western blot assays of non-polarized and polarized Calu-3 cells............................ S13

Figure S6. Entire western blot membranes from assay 1 (a) and 2 (b) S17

Proteome Discoverer workflows used for protein identification and quantification. S18

Table S1: list of antibodies used for western blot and fluorescence microscopy analysis (xlsx).

Table S2: list of the regulated genes and proteins for Non-polarized and Polarized Calu-3 cells and their corresponding gene ontology analysis (xlsx).

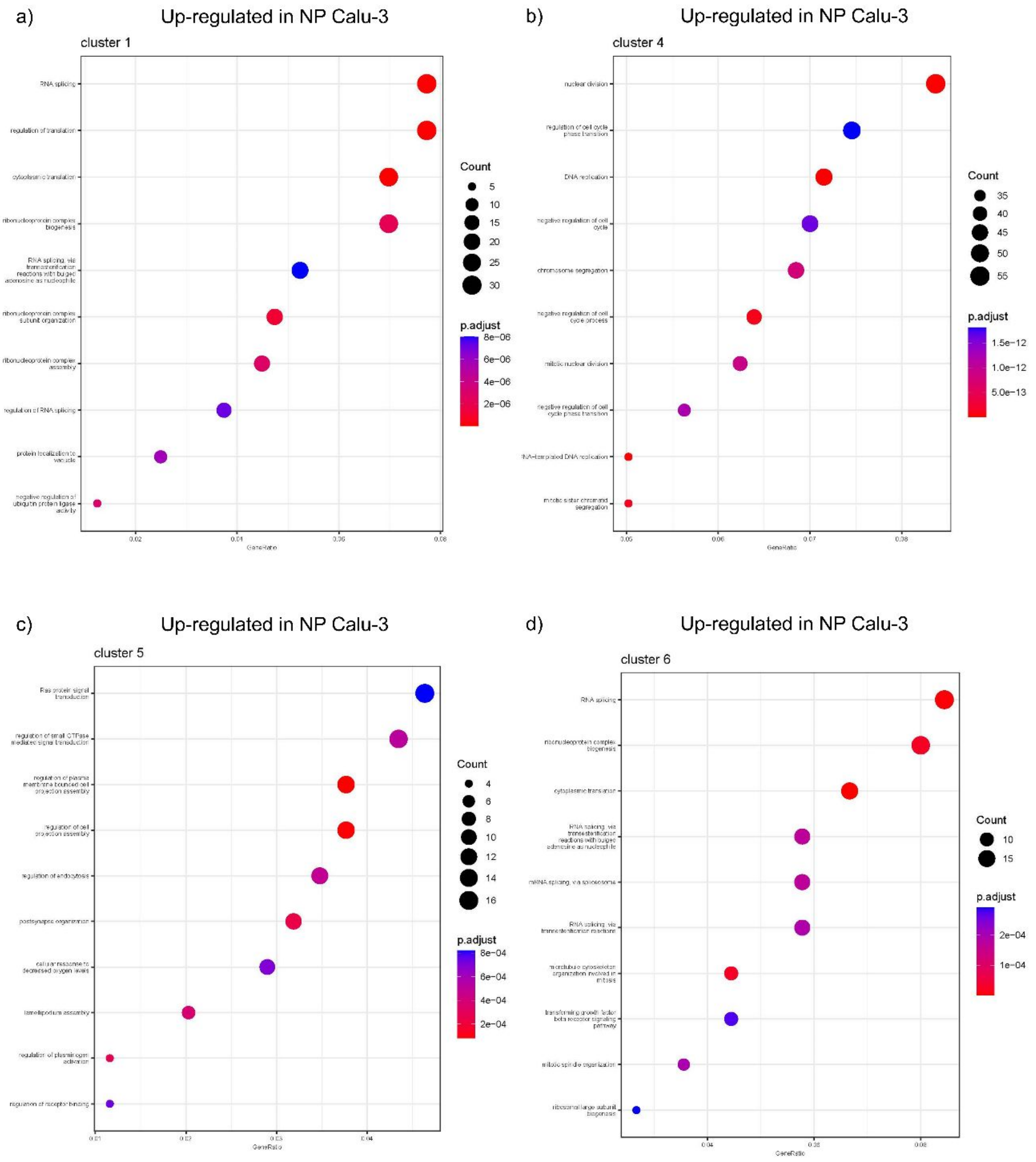
a)
Up-regulated in NP Calu-3
cluster 1
RNA splicing
regulation of translation
ribonucleoprotein complex biogenesis
ribonucleoprotein complex subunit organization
ribonucleoprotein complex assembly
regulation of RNA splicing
GeneRatio
Count
5
10
15
20
25
30
p.adjust
8e−06
6e−06
4e−06
2e−06
b)
Up-regulated in NP Calu-3
cluster 4
nuclear division
DNA replication
negative regulation of cell cycle
chromosome segregation
negative regulation of cell cycle process
mitotic nuclear division
negative regulation of cell cycle phase transition
GeneRatio
Count
35
40
45
50
55
p.adjust
1.5e−12
1.0e−12
5.0e−13
c)
Up-regulated in NP Calu-3
cluster 5
Ras protein signal transduction
regulation of plasma membrane bounded cell projection assembly
regulation of cell projection assembly
regulation of endocytosis
lamellipodium assembly
GeneRatio
Count
4
6
8
10
12
14
16
p.adjust
8e−04
6e−04
4e−04
2e−04
d)
Up-regulated in NP Calu-3
cluster 6
RNA splicing
ribonucleoprotein complex biogenesis
mRNA splicing, via spliceosome
transforming growth factor beta receptor signaling pathway
mitotic spindle organization
ribosomal large subunit biogenesis
GeneRatio
Count
10
15
p.adjust
2e−04
1e−04

e) Up-regulated in NP Calu-3

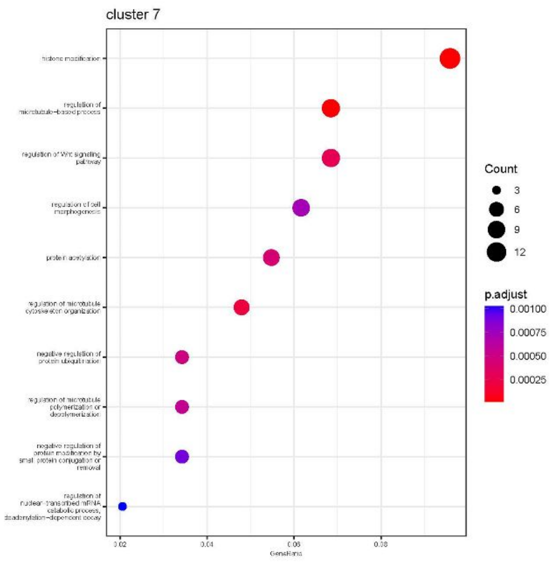

f) Up-regulated in NP Calu-3

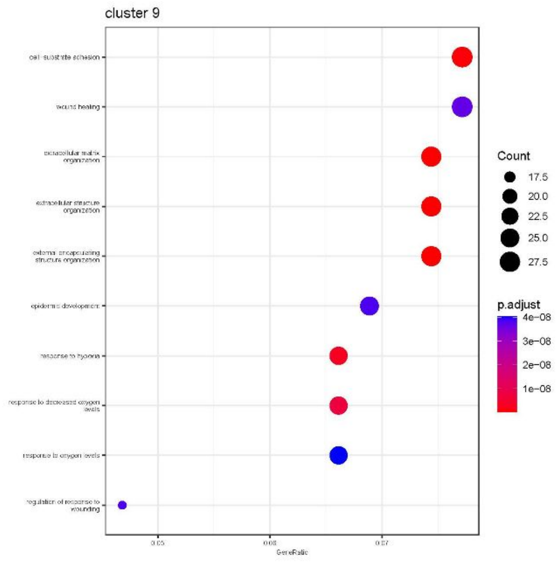

g) Up-regulated in NP Calu-3

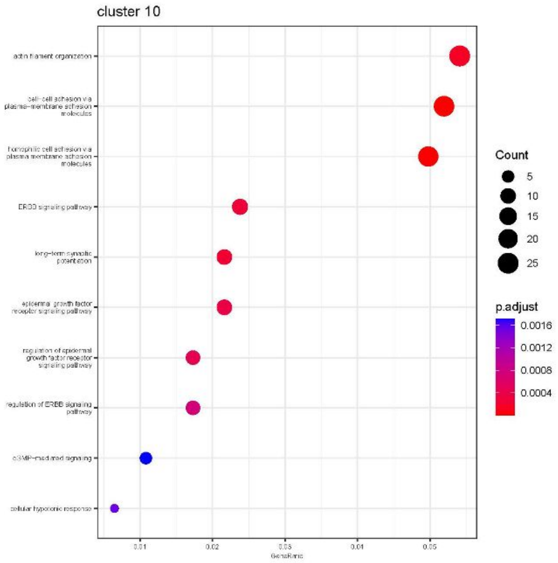

h) Up-regulated in NP Calu-3

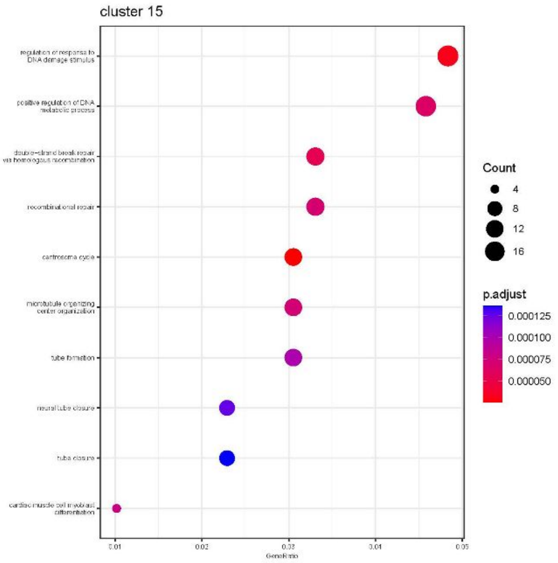

i) Up-regulated in P Calu-3

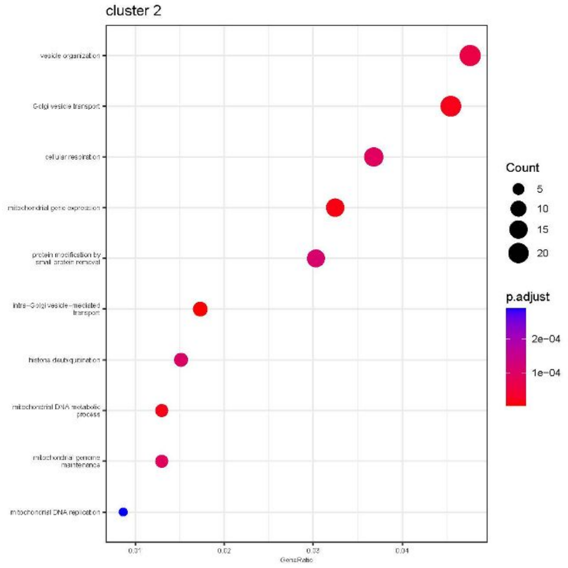

j) Up-regulated in P Calu-3

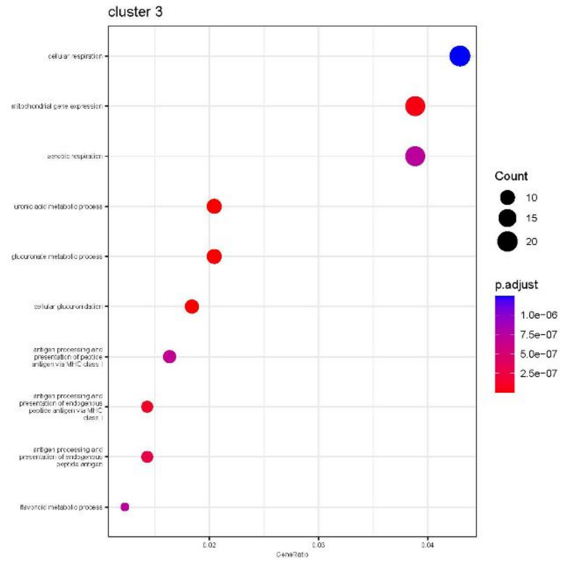

k) Up-regulated in P Calu-3

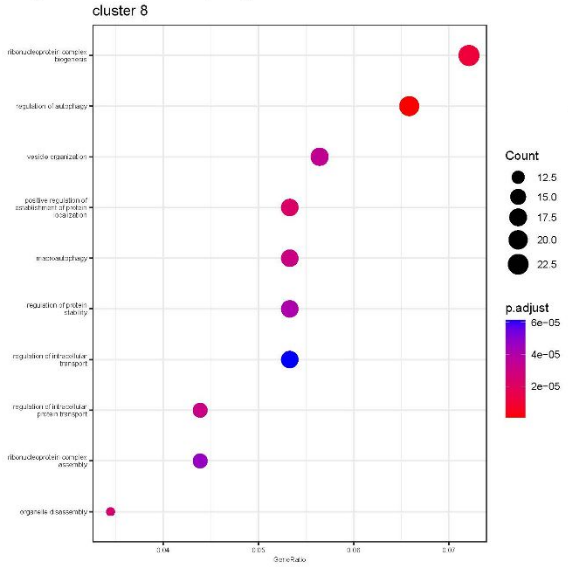

l) Up-regulated in P Calu-3

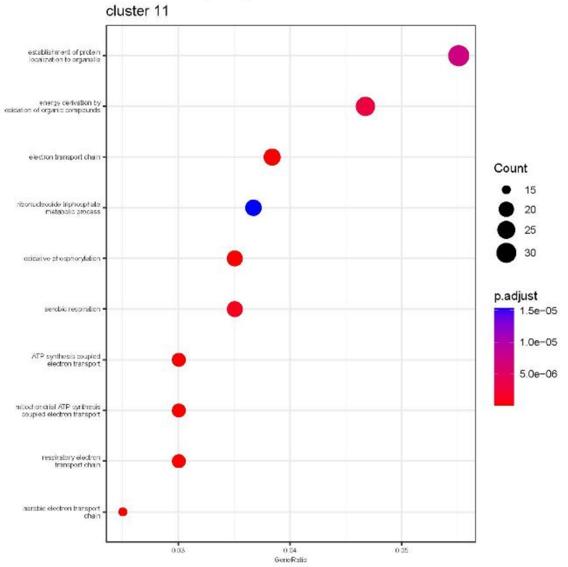

m)

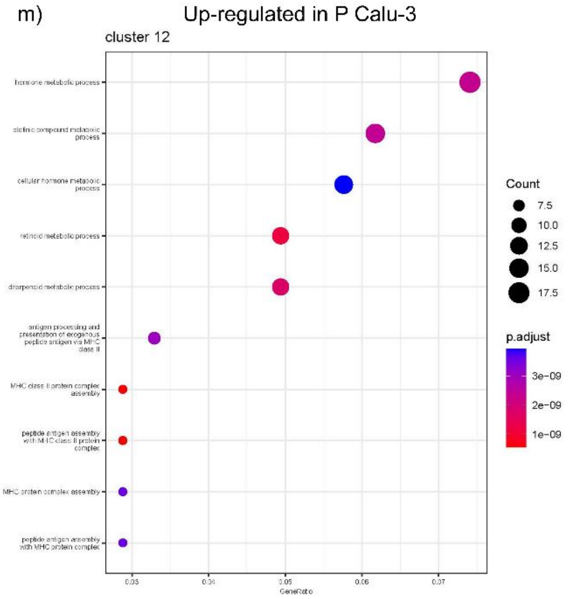

n)

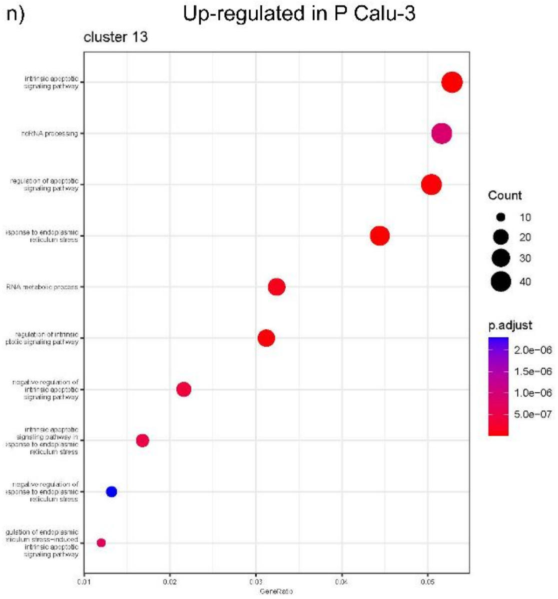

o)

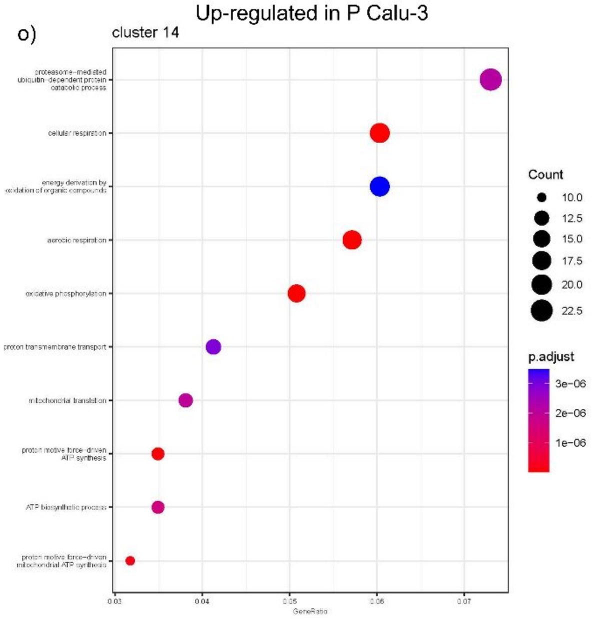

Figure S1. Gene clusters regulation in Non-polarized and Polarized Calu-3 cells. Up-regulated gene clusters in Non-polarized Calu-3 cells (**a, b, c, d, e, f, g** and **h**) and Polarized Calu-3 cells (**I, j, k, l, m, n** and **o**). The gene enrichment analysis was performed using clusterProfiler.

a)

Transcriptomics

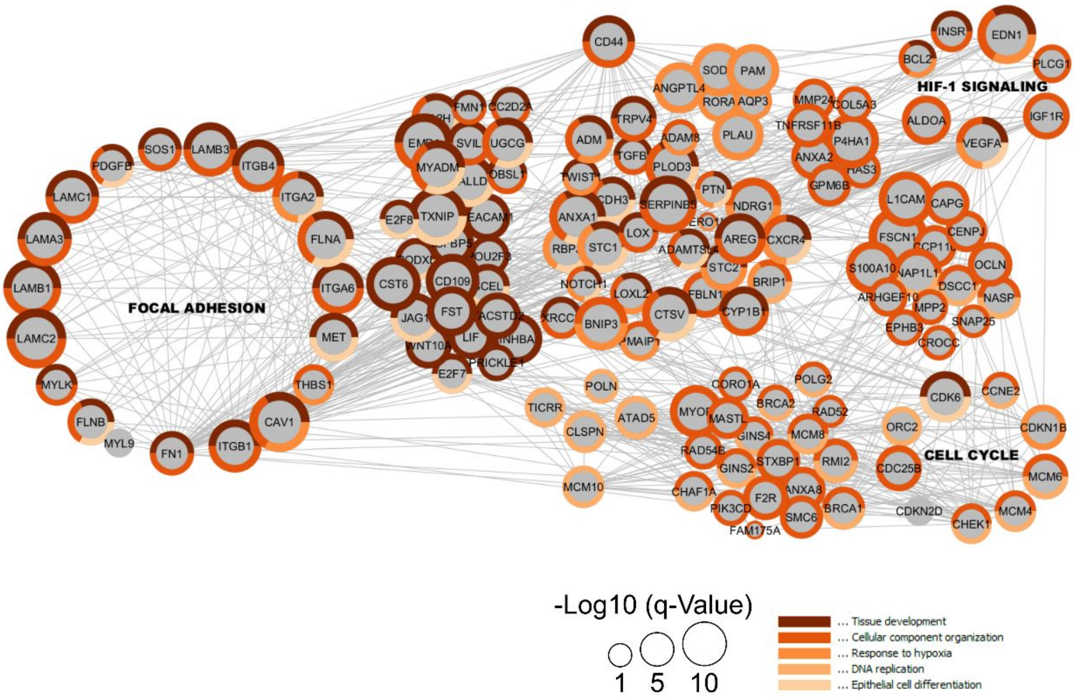

b)

Proteomics

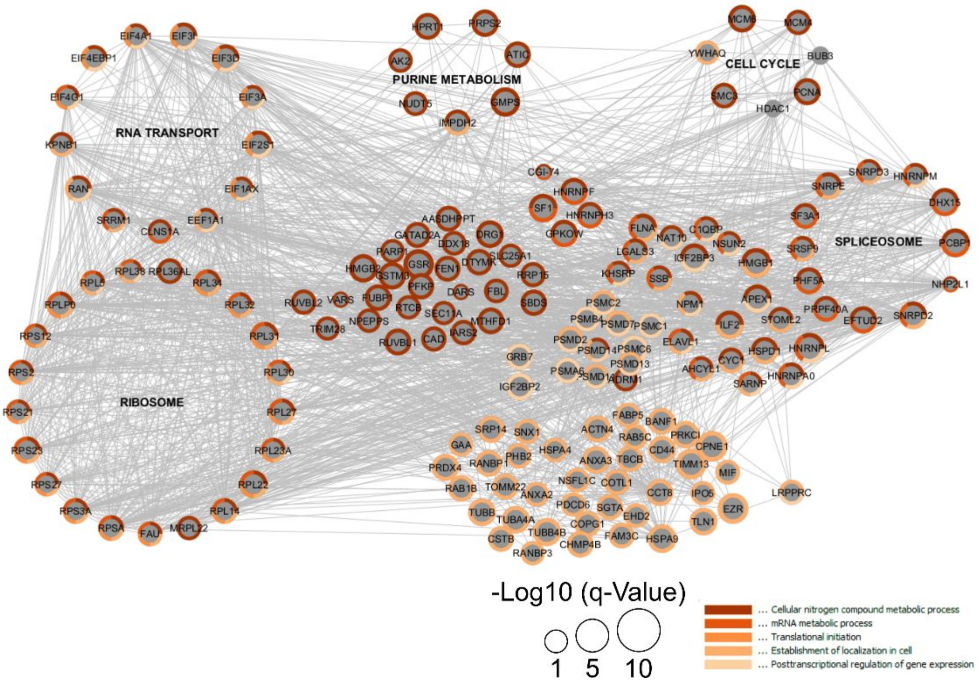

Figure S2. Network interaction analysis of Non-polarized Calu-3 cells. Network interaction of up-regulated RNA (a) and protein (b) of Non-polarized Calu-3 cells was performed using Cytoscape software. Gene ontology analysis was performed using only annotated genes with Benjamini-Hochberg FDR < 0.05 correction, in which the rings surrounding the RNA/proteins nodes correspond to the top five enriched biological processes. Highlighted clusters were obtained based on the KEEG Pathways activated. Node sizes correspond to the q-Value (-Log10) after differential regulation analysis using t-test with Benjamini-Hochberg FDR < 0.05 correction.

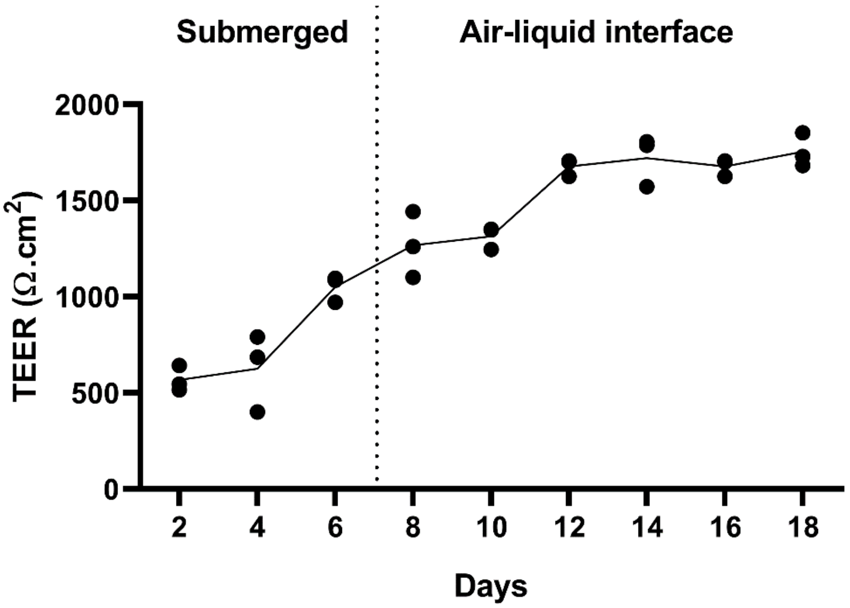

Figure S3. Evolution of Trans-epithelial electrical resistance (TEER) in polarized Calu-3 cells cultured in Air-liquid Interface. Calu-3 cells (n: 3) cultured in inserts presents growing TEER values, corresponding to the cell layer formation in the submerged conditions and the tight junctions in the Air-liquid interface.

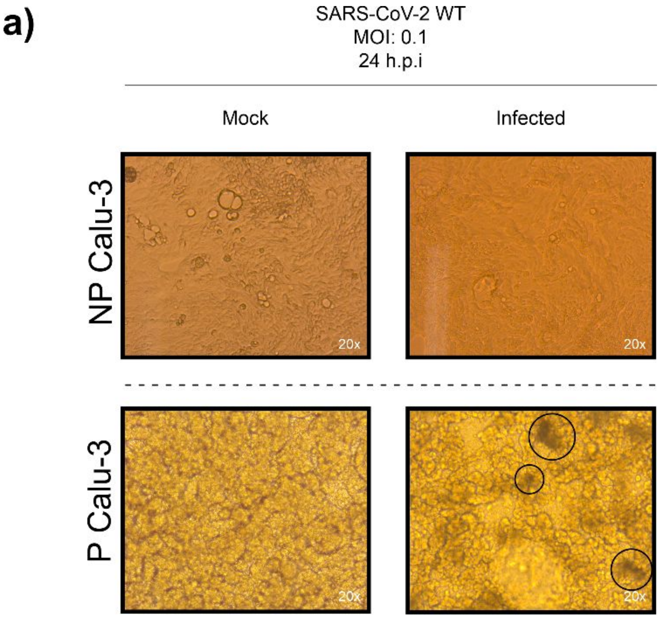

Figure S4. Cytopathic effect in Non-polarized and Polarized Calu-3 cells infected with SARS-CoV-2. Polarized (P) Calu-3 cells infected with SARS-CoV-2 wild type (MOI: 0.1) presents more cell debris (black circles) after 24 hors of infection in comparison with Non-polarized (NP) Calu-3 cells.

a)

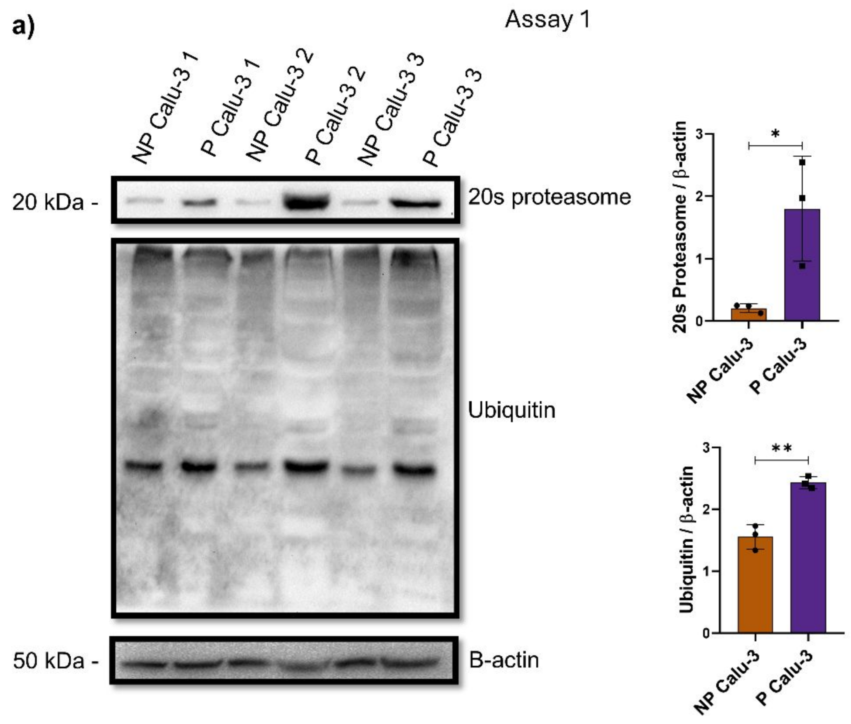
Assay 1
NP Calu-3 1
P Calu-3 1
NP Calu-3 2
P Calu-3 2
NP Calu-3 3
P Calu-3 3
20 kDa -
20s proteasome
Ubiquitin
50 kDa -
B-actin
20s Proteasome / β-actin
NP Calu-3
P Calu-3
Ubiquitin / β-actin
*
**

b)

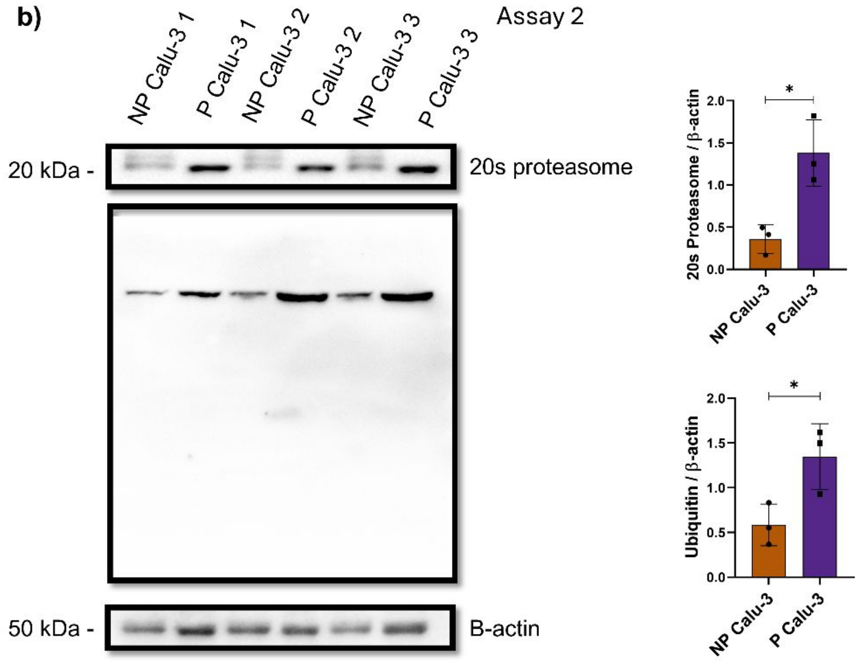
Assay 2
NP Calu-3 1
P Calu-3 1
NP Calu-3 2
P Calu-3 2
NP Calu-3 3
P Calu-3 3
20 kDa -
20s proteasome
50 kDa -
B-actin
20s Proteasome / β-actin
NP Calu-3
P Calu-3
Ubiquitin / β-actin
*

c)

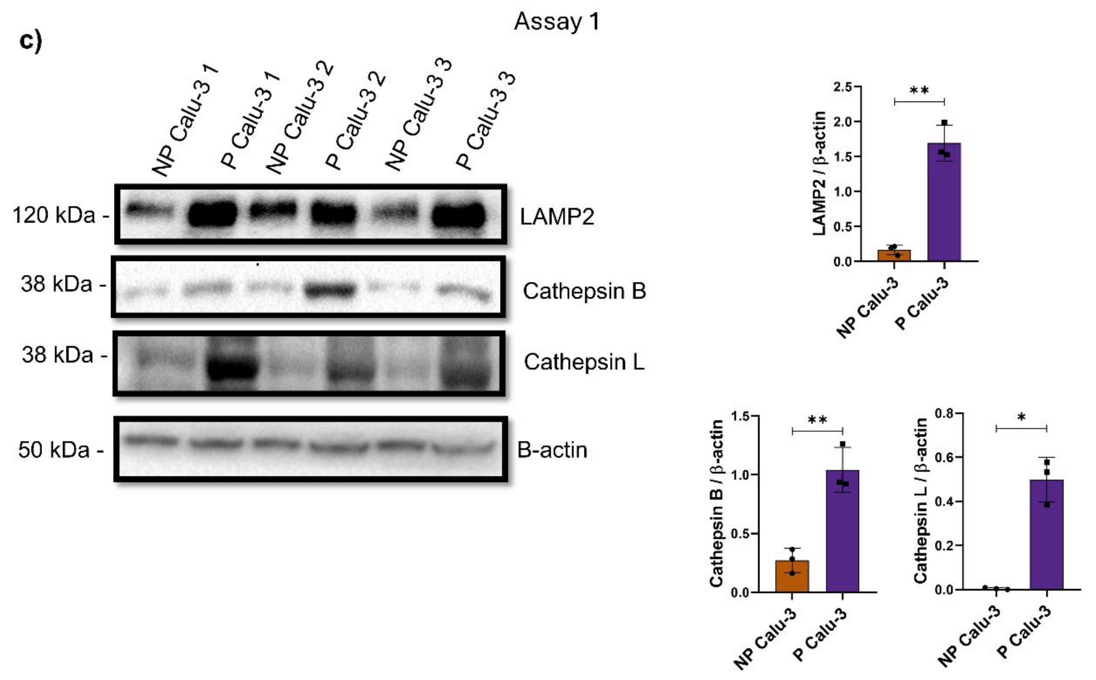
Assay 1
NP Calu-3 1
P Calu-3 1
NP Calu-3 2
P Calu-3 2
NP Calu-3 3
P Calu-3 3
120 kDa -
LAMP2
38 kDa -
Cathepsin B
38 kDa -
Cathepsin L
50 kDa -
B-actin
LAMP2 / β-actin
Cathepsin B / β-actin
Cathepsin L / β-actin
**
*
NP Calu-3
P Calu-3

d)

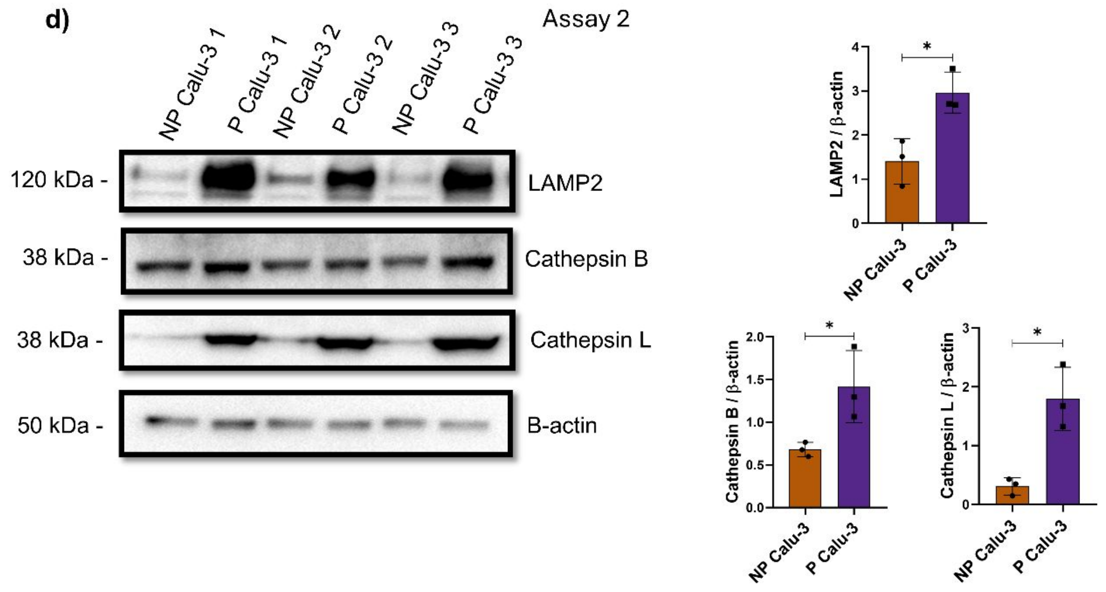
Assay 2
NP Calu-3 1
P Calu-3 1
NP Calu-3 2
P Calu-3 2
NP Calu-3 3
P Calu-3 3
120 kDa -
LAMP2
38 kDa -
Cathepsin B
38 kDa -
Cathepsin L
50 kDa -
B-actin
LAMP2 / β-actin
Cathepsin B / β-actin
Cathepsin L / β-actin
*
NP Calu-3
P Calu-3

e)

Assay 1

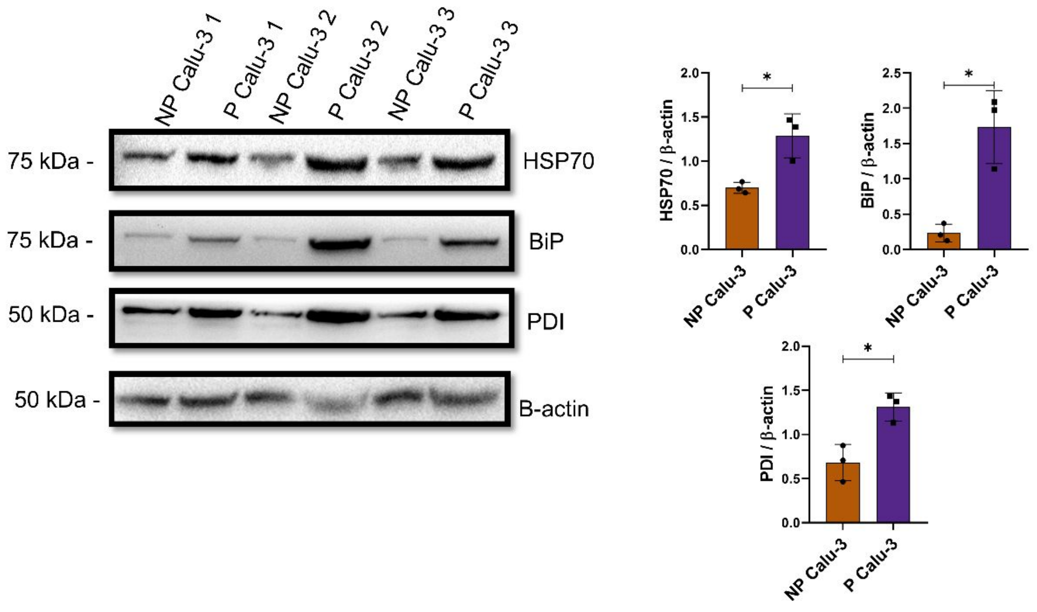

f)

Assay 2

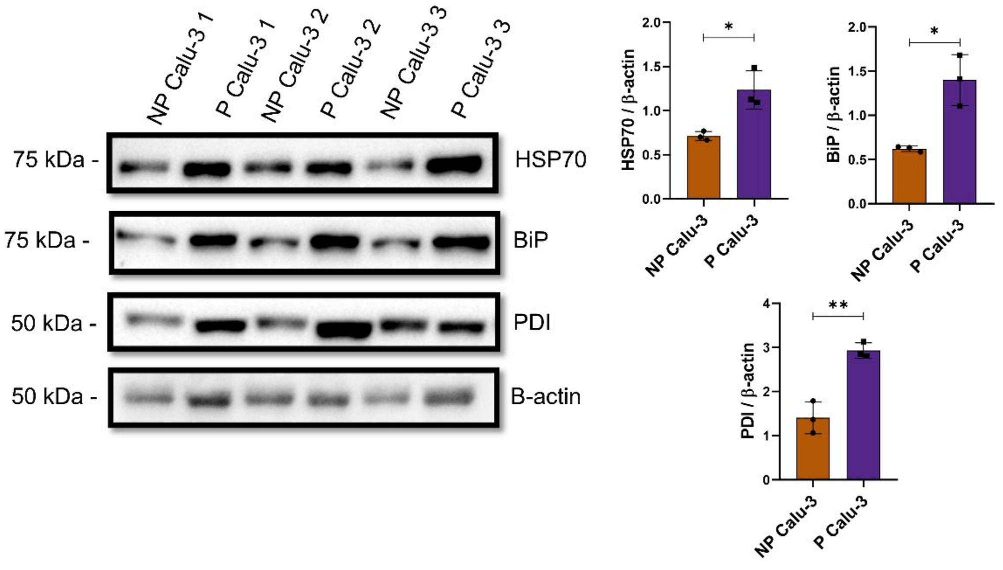

g)

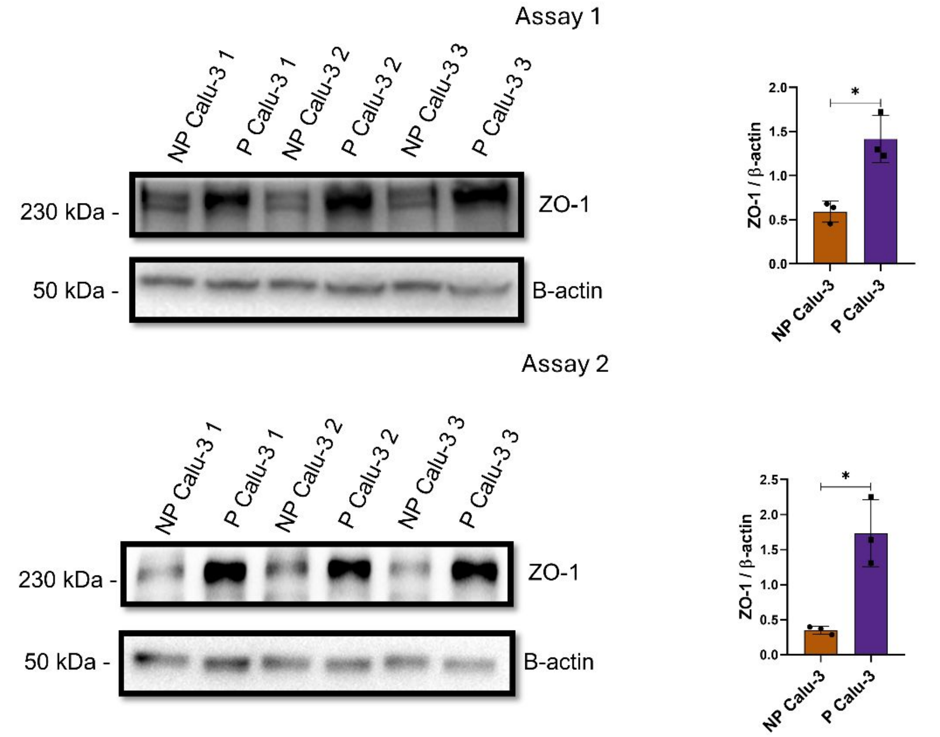
Assay 1
NP Calu-3 1
P Calu-3 1
NP Calu-3 2
P Calu-3 2
NP Calu-3 3
P Calu-3 3
230 kDa -
ZO-1
50 kDa -
B-actin
ZO-1 / β-actin
2.0
1.5
1.0
0.5
0.0
*
NP Calu-3
P Calu-3
Assay 2
NP Calu-3 1
P Calu-3 1
NP Calu-3 2
P Calu-3 2
NP Calu-3 3
P Calu-3 3
230 kDa -
ZO-1
50 kDa -
B-actin
ZO-1 / β-actin
2.5
2.0
1.5
1.0
0.5
0.0
*
NP Calu-3
P Calu-3

h)

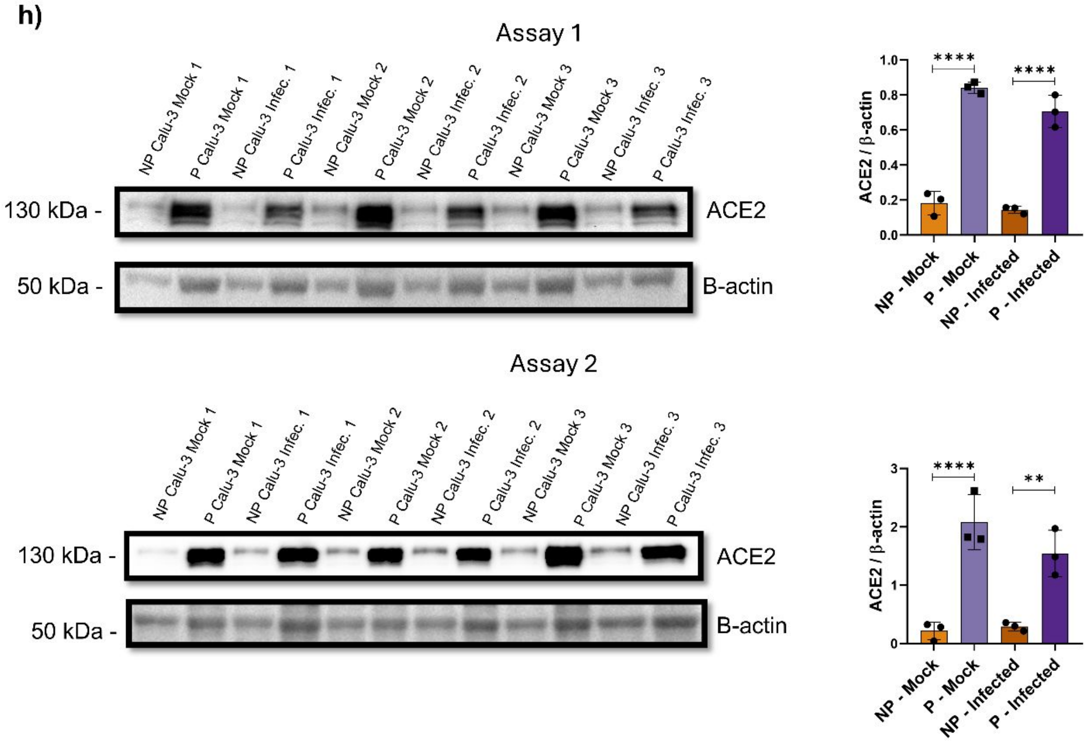
Assay 1
NP Calu-3 Mock 1
P Calu-3 Mock 1
NP Calu-3 Infec. 1
P Calu-3 Infec. 1
NP Calu-3 Mock 2
P Calu-3 Mock 2
NP Calu-3 Infec. 2
P Calu-3 Infec. 2
NP Calu-3 Mock 3
P Calu-3 Mock 3
NP Calu-3 Infec. 3
P Calu-3 Infec. 3
130 kDa -
ACE2
50 kDa -
B-actin
ACE2 / β-actin
1.0
0.8
0.6
0.4
0.2
0.0
****
****
NP - Mock
P - Mock
NP - Infected
P - Infected
Assay 2
NP Calu-3 Mock 1
P Calu-3 Mock 1
NP Calu-3 Infec. 1
P Calu-3 Infec. 1
NP Calu-3 Mock 2
P Calu-3 Mock 2
NP Calu-3 Infec. 2
P Calu-3 Infec. 2
NP Calu-3 Mock 3
P Calu-3 Mock 3
NP Calu-3 Infec. 3
P Calu-3 Infec. 3
130 kDa -
ACE2
50 kDa -
B-actin
ACE2 / β-actin
3
2
1
0
****
**
NP - Mock
P - Mock
NP - Infected
P - Infected

i)

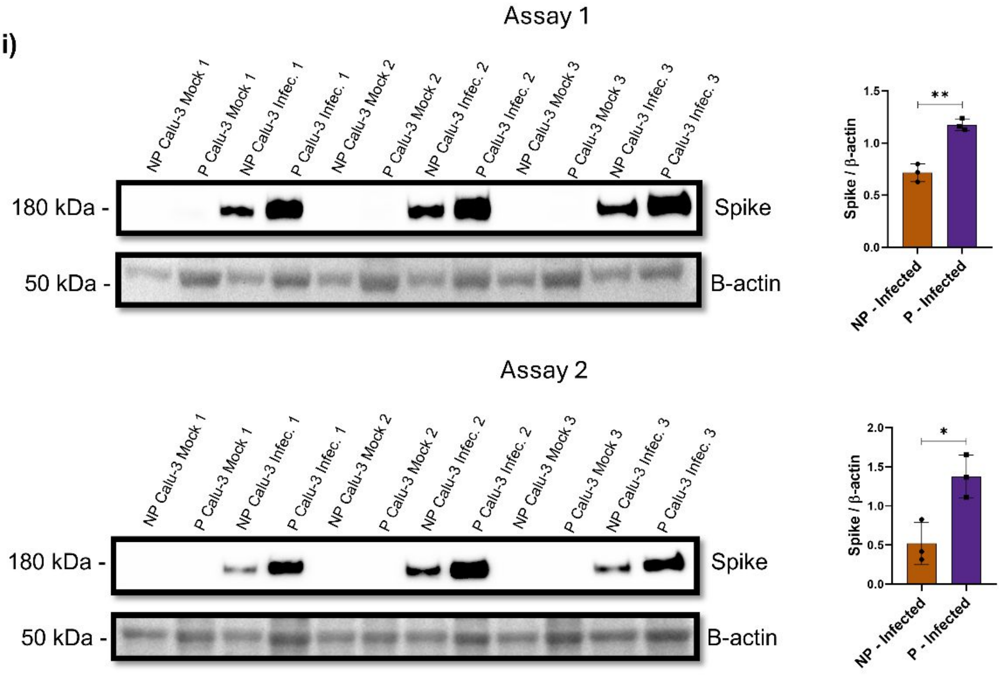

Figure S5. Western blot assays of non-polarized and polarized Calu-3 cells. Results shown in assay 1 (**a**) and assay (**b**) represent components of the Ubiquitin-proteasome pathway. Results shown in in assay 1 (**c**) and assay (**d**) represent components of lysosome. Results shown in assay 1 (**e**) and assay 2 (**f**) represent components of Proein processing in ER pathway. ZO-1 (**g**) protein levels represent Tight junctions. ACE2 (**h**) and Spike (**1**) protein levels represents the analysis of Calu-3 cells susceptibility to SARS-CoV-2 infection in non-polarized and polarized cell condition. The experiments were performed in triplicates and each dot in the graphs represents a replicate. The asterisk symbol (*) represents the statistical differences (**** p<0.0001; *** p<0.001; ** p<0.005; *p<0.05). The endogenous beta-actin blot reported for the 20S, Ubiquitin, HSP70, BiP and PDI proteins is the same since the same membrane was used to reveal these proteins. The endogeneous beta acting blot reported for ZO-1, LAMP2, CatB and CatL proteins is the same since the same membrane was used to reveal these proteins. The endogeneous beta acting blot reported for ACE-2 and SPIKE proteins is the same since the same membrane was used to reveal these proteins. This is valid for assay 1 and assay 2.

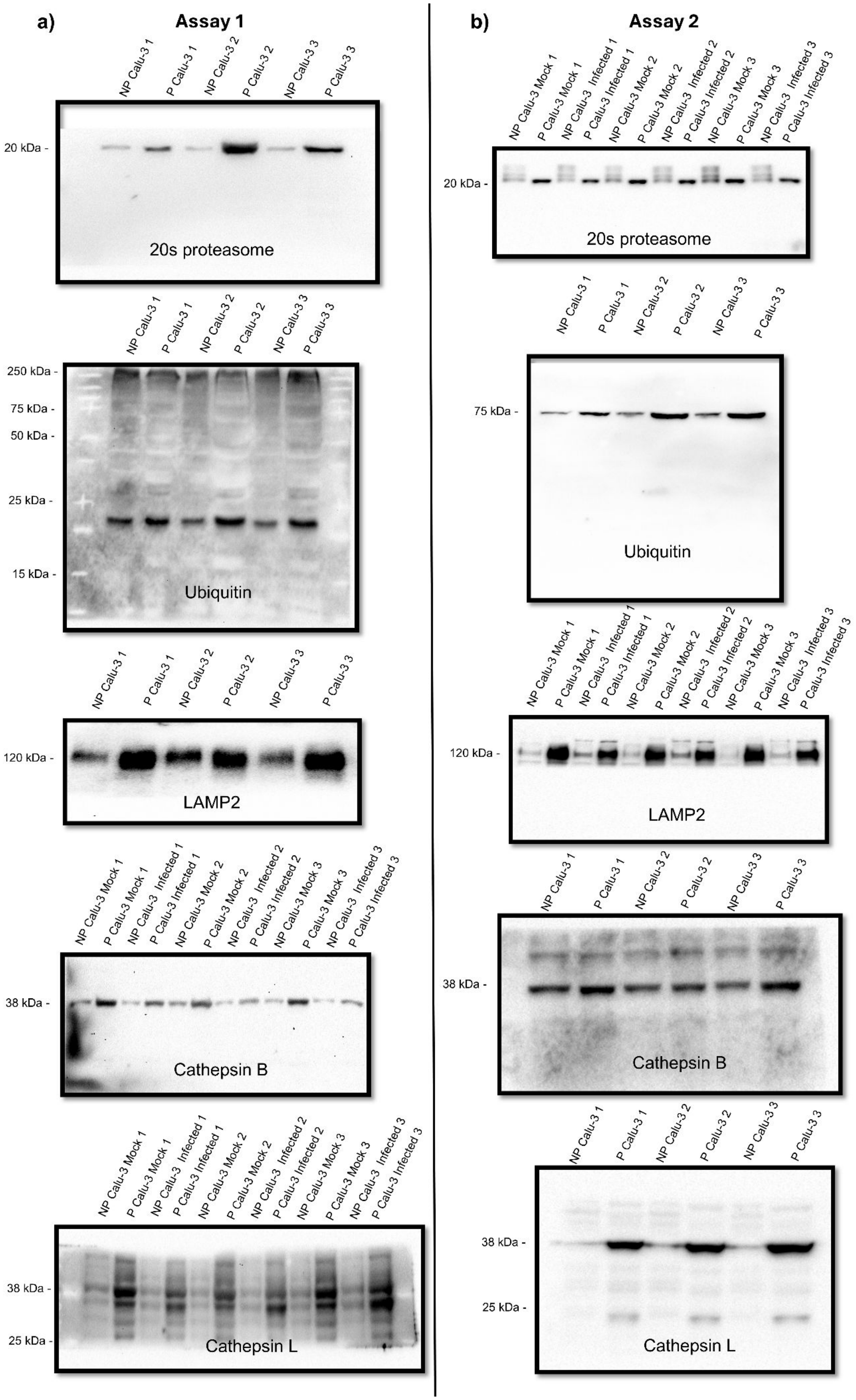
a)
Assay 1
NP Calu-3 1
P Calu-3 1
NP Calu-3 2
P Calu-3 2
NP Calu-3 3
P Calu-3 3
20 kDa -
20s proteasome
250 kDa -
75 kDa -
50 kDa -
25 kDa -
15 kDa -
Ubiquitin
120 kDa -
LAMP2
NP Calu-3 Mock 1
P Calu-3 Mock 1
NP Calu-3 Infected 1
P Calu-3 Infected 1
NP Calu-3 Mock 2
P Calu-3 Mock 2
NP Calu-3 Infected 2
P Calu-3 Infected 2
NP Calu-3 Mock 3
P Calu-3 Mock 3
NP Calu-3 Infected 3
P Calu-3 Infected 3
38 kDa -
Cathepsin B
25 kDa -
Cathepsin L
b)
Assay 2
20s proteasome
Ubiquitin
LAMP2
Cathepsin B
Cathepsin L

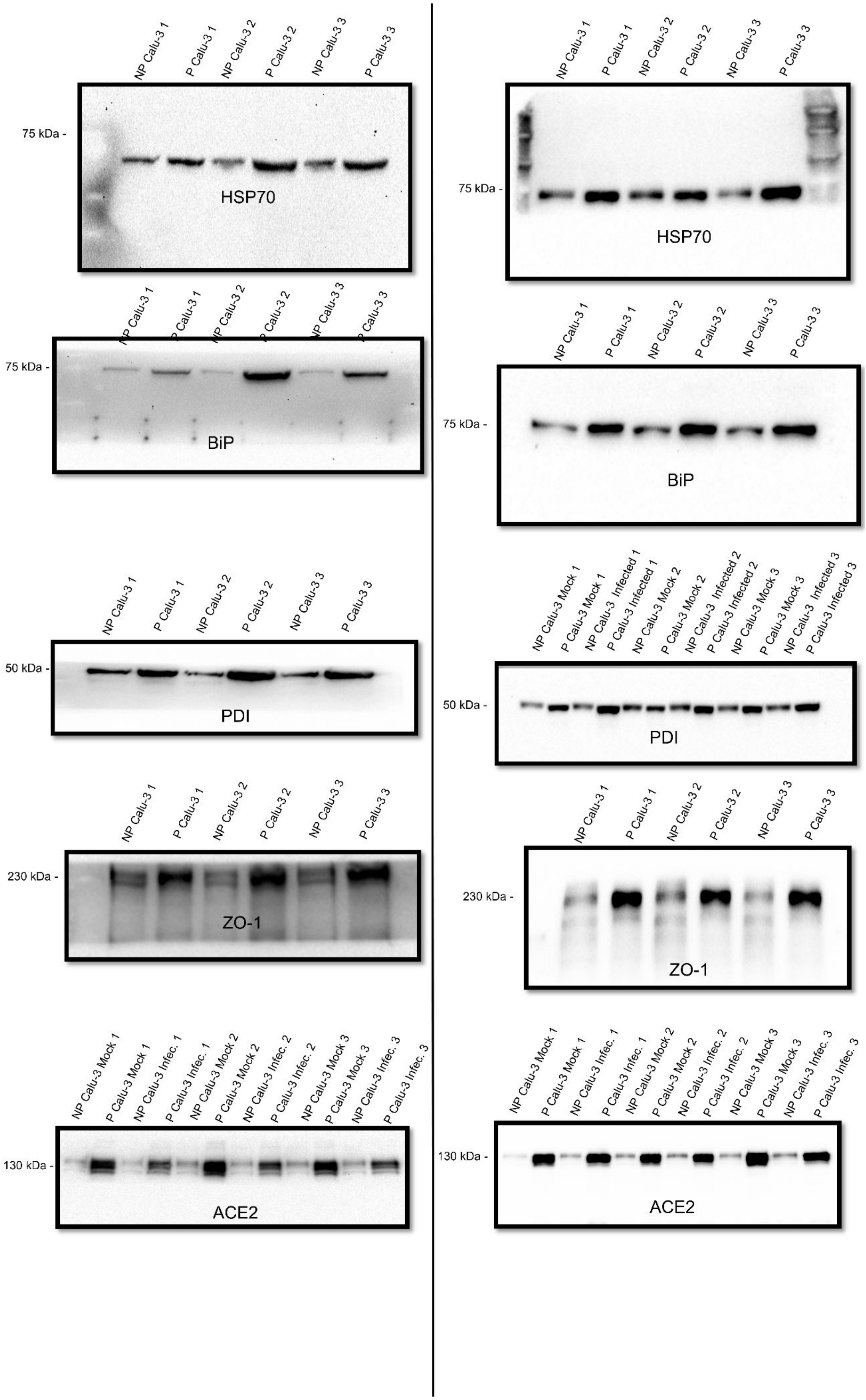
NP Calu-3 1
P Calu-3 1
NP Calu-3 2
P Calu-3 2
NP Calu-3 3
P Calu-3 3
75 kDa -
HSP70
BiP
50 kDa -
PDI
230 kDa -
ZO-1
NP Calu-3 Mock 1
P Calu-3 Mock 1
NP Calu-3 Infected 1
P Calu-3 Infected 1
NP Calu-3 Mock 2
P Calu-3 Mock 2
NP Calu-3 Infected 2
P Calu-3 Infected 2
NP Calu-3 Mock 3
P Calu-3 Mock 3
NP Calu-3 Infected 3
P Calu-3 Infected 3
NP Calu-3 Infec. 1
P Calu-3 Infec. 1
NP Calu-3 Infec. 2
P Calu-3 Infec. 2
NP Calu-3 Infec. 3
P Calu-3 Infec. 3
130 kDa -
ACE2

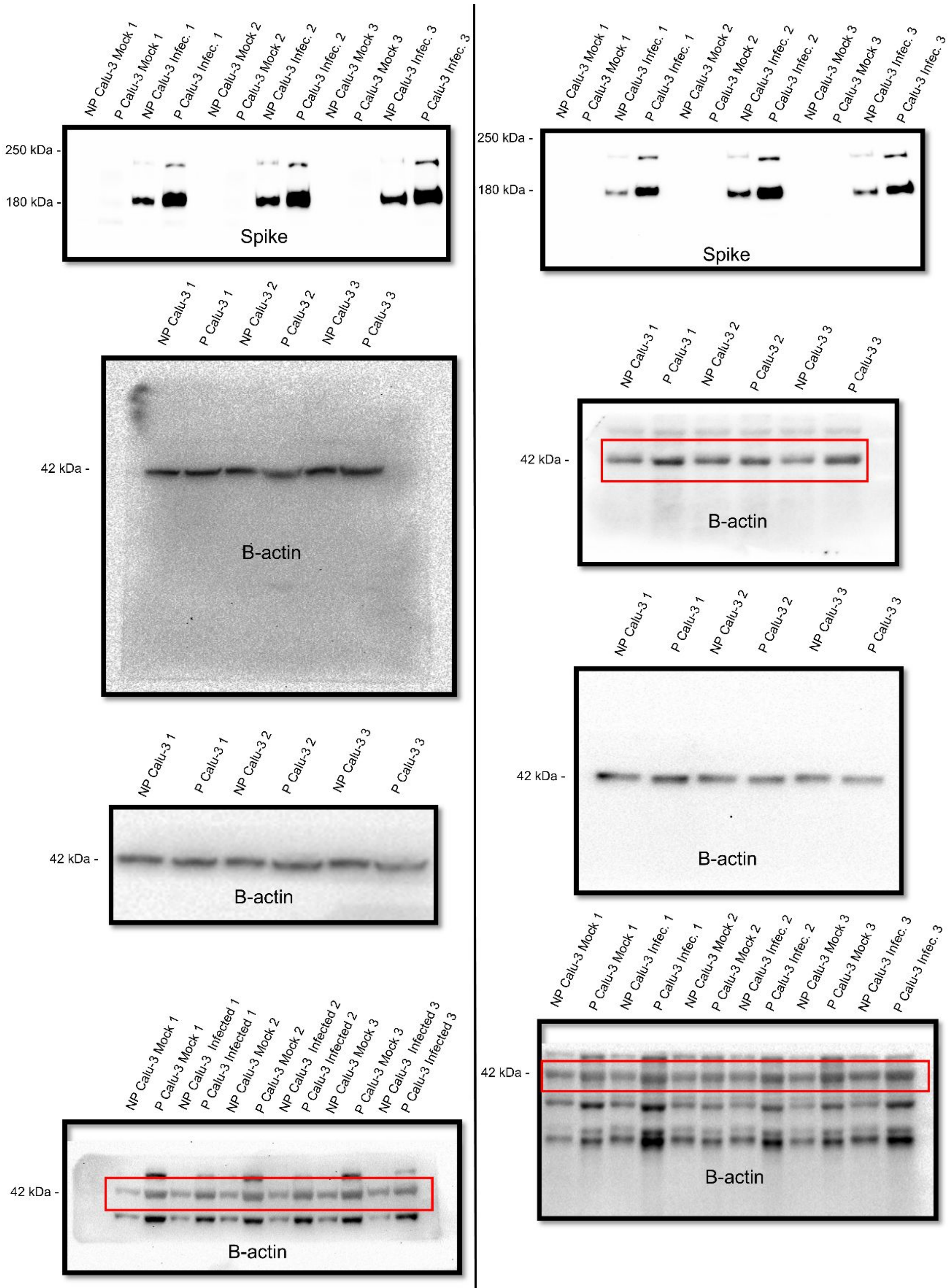
NP Calu-3 Mock 1
P Calu-3 Mock 1
NP Calu-3 Infec. 1
P Calu-3 Infec. 1
NP Calu-3 Mock 2
P Calu-3 Mock 2
NP Calu-3 Infec. 2
P Calu-3 Infec. 2
NP Calu-3 Mock 3
P Calu-3 Mock 3
NP Calu-3 Infec. 3
P Calu-3 Infec. 3
250 kDa -
180 kDa -
Spike
NP Calu-3 1
P Calu-3 1
NP Calu-3 2
P Calu-3 2
NP Calu-3 3
P Calu-3 3
42 kDa -
B-actin
NP Calu-3 Mock 1
P Calu-3 Mock 1
NP Calu-3 Infected 1
P Calu-3 Infected 1
NP Calu-3 Mock 2
P Calu-3 Mock 2
NP Calu-3 Infected 2
P Calu-3 Infected 2
NP Calu-3 Mock 3
P Calu-3 Mock 3
NP Calu-3 Infected 3
P Calu-3 Infected 3

Figure S6. Entire western blot membranes from assay 1 (a) and 2 (b). The endogeneous beta-actin blot reported for the 20S, Ubiquitin, HSP70, BiP and PDI proteins is the same since the same membrane was used to reveal these proteins. The endogeneous beta acting blot reported for ZO-1, LAMP2, CatB and CatL proteins is the same since the same membrane was used to reveal these proteins. The endogeneous beta acting blot reported for ACE-2 and SPIKE proteins is the same since the same membrane was used to reveal these proteins. This is valid for assay 1 and assay 2.

Proteome Discoverer workflows used for protein identification and quantification.

Consensus Step : Workflow

==

Result name: ALI_Oct2022

Result file: V:\MED\ClinMed_Biomed\Restrict\Chung Group\Livia\RawFiles\2022\10_2022\Deivid\ALI_Oct2022\ALI_Oct2022\ALI_Oct2022.pdResult

Description: Result filtered for high confident peptides, with enhanced peptide and protein annotations. Add FASTA file with common contaminants to the Protein Marker node. Quan abunaces are normalized to the same total peptide amount per channel and scaled, so that the average abunce per protein and peptide is 100.

Workflow based on template: CWF_Comprehensive_Enhanced Annotation_LFQ_and_Precursor_Quan

Creation date: 10/12/2022 10:27:14 PM

Created with Discoverer version: 2.4.1.15

The workflow tree:

|-(0) MSF Files

|-(1) PSM Grouper

|-(2) Peptide Validator

|-(3) Peptide and Protein Filter

|-(4) Protein Scorer

|-(5) Protein Grouping

|-(6) Peptide in Protein Annotation

|-(7) Protein FDR Validator

|-(10) Feature Mapper

|-(11) Precursor Ions Quantifier

Post-processing nodes:

|-(12) Result Statistics

|-(13) Display Settings

|-(14) Data Distributions

Processing node 0: MSF Files

----

1. Storage Settings:

- Spectra to Store: Identified or Quantified

- Feature Traces to Store: All

2. Merging of Identified Peptide and Proteins:

- Merge Mode: Globally by Search Engine Type

3. FASTA Title Line Display:

- Reported FASTA Title Lines: Best match

- Title Line Rule: standard

4. PSM Filters:

- Maximum Delta Cn: 0.05

- Maximum Rank: 0

- Maximum Delta Mass: 0 ppm

Hidden Parameters:

- MSF File(s): V:\Chung Group\Livia\RawFiles\10_2022\Deivid\ALI_Oct2022\ALI_Oct2022\ALI_Oct2022.msf

----

Processing node 1: PSM Grouper

----

1. Peptide Group Modifications:

- Site Probability Threshold: 75

Processing node 2: Peptide Validator

----

1. General Validation Settings:

- Validation Mode: Automatic (Control peptide level error rate if possible)

- Target FDR (Strict) for PSMs: 0.01

- Target FDR (Relaxed) for PSMs: 0.05

- Target FDR (Strict) for Peptides: 0.01

- Target FDR (Relaxed) for Peptides: 0.05

2. Specific Validation Settings:

- Validation Based on: q-Value

- Target/Decoy Selection for PSM Level FDR Calculation Based on Score: Automatic

- Reset Confidences for Nodes without Decoy Search (Fixed score thresholds): False

----

Processing node 3: Peptide and Protein Filter

----

1. Peptide Filters:

- Peptide Confidence At Least: High

- Keep Lower Confident PSMs: False

- Minimum Peptide Length: 6

- Remove Peptides Without Protein Reference: False

2. Protein Filters:

- Minimum Number of Peptide Sequences: 1

- Count Only Rank 1 Peptides: False

- Count Peptides Only for Top Scored Protein: False

Processing node 4: Protein Scorer

No parameters

Processing node 5: Protein Grouping

----

1. Protein Grouping:

- Apply strict parsimony principle: True

Processing node 6: Peptide in Protein Annotation

----

1. Flanking Residues:

- Annotate Flanking Residues of the Peptide: True

- Number Flanking Residues in Connection Tables: 1

2. Modifications in Peptide:

- Protein Modifications Reported: Only for Master Proteins

3. Modifications in Protein:

- Modification Sites Reported: All And Specific

- Minimum PSM Confidence: High

- Report Only PTMs: True

4. Positions in Protein:

- Protein Positions for Peptides: Only for Master Proteins

Processing node 7: Protein FDR Validator

----

1. Confidence Thresholds:

- Target FDR (Strict): 0.01

- Target FDR (Relaxed): 0.05

Processing node 10: Feature Mapper

----

1. Chromatographic Alignment:

- Perform RT Alignment: True

- Maximum RT Shift [min]: 10

- Mass Tolerance: 10 ppm

- Parameter Tuning: Coarse

2. Feature Linking and Mapping:

- RT Tolerance [min]: 0

- Mass Tolerance: 0 ppm

- Min. S/N Threshold: 5

Processing node 11: Precursor Ions Quantifier

----

1. General Quantification Settings:

- Peptides to Use: Unique + Razor

- Consider Protein Groups for Peptide Uniqueness: True

- Use Shared Quan Results: True

- Reject Quan Results with Missing Channels: False

2. Precursor Quantification:

- Precursor Abundance Based On: Intensity

- Min. # Replicate Features [%]: 0

3. Normalization and Scaling:

- Normalization Mode: Total Peptide Amount

- Scaling Mode: None

4. Exclude Peptides from Protein Quantification:

- For Normalization: Use All Peptides

- For Protein Roll-Up: Use All Peptides

- For Pairwise Ratios: Exclude Modified

5. Quan Rollup and Hypothesis Testing:

- Protein Abundance Calculation: Summed Abundances

- N for Top N: 3

- Protein Ratio Calculation: Pairwise Ratio Based

- Maximum Allowed Fold Change: 100

- Imputation Mode: None

- Hypothesis Test: t-test (Background Based)

6. Quan Ratio Distributions:

- 1st Fold Change Threshold: 2

- 2nd Fold Change Threshold: 4

- 3rd Fold Change Threshold: 6

- 4th Fold Change Threshold: 8

- 5th Fold Change Threshold: 10

Processing node 12: Result Statistics

No parameters

Processing node 13: Display Settings

----

1. General:

- Filter Set:

Filter Set MasterProteinFilter.filterset contains the following filters:

Row Filter for TargetProtein:

Master is equal to Master

###

'magellan filter set' 1 'MasterProteinFilter.filterset' FiltersetProperties 1 'LastFileName' 'C:\Users\frank.berg\Desktop\MasterProteinFilter.filterset' Filter 'TargetProtein' 1 NARY_AND 1 = FilterConditionProperties 1 'NamedComparableFilterCondition/DisplayPropertyHint' 'Master' property 'Thermo.PD.EntityDataFramework.MasterProteinAssessment, Thermo.Magellan.EntityDataFramework' 'IsMasterProtein' constant 'Thermo.PD.EntityDataFramework.MasterProteinAssessment, Thermo.Magellan.EntityDataFramework' 'IsMasterProtein'

Processing node 14: Data Distributions

----

1. ID Distributions (Bottom-up):

- Peptides to Use: Only unique peptides based on protein groups

----

Workflow messages:

10/13/2022 12:12 AM Job Execution: Processing V:\Chung Group\Livia\RawFiles\10_2022\Deivid\ALI_Oct2022\ALI_Oct2022\ALI_Oct2022.pdResult

10/13/2022 12:12 AM (0): MSF Files: V:\Chung Group\Livia\RawFiles\10_2022\Deivid\ALI_Oct2022\ALI_Oct2022\ALI_Oct2022.msf

10/13/2022 12:12 AM (0): MSF Files: All 1 files are ready for processing.

10/13/2022 12:12 AM (0): MSF Files: Start transferring results of 1 msf files...

10/13/2022 12:12 AM (0): MSF Files: Transferred 26049 Proteins to result file in 39.9 s.

10/13/2022 12:13 AM (0): MSF Files: Transferred 23668 Decoy Proteins to result file in 33.8 s.

10/13/2022 12:16 AM (0): MSF Files: Added 154146 Mass Spectra to result file.

10/13/2022 12:19 AM (0): MSF Files: Added 1712351 traces to result file.

10/13/2022 12:19 AM (0): MSF Files: Added 6 Input Files to result file.

10/13/2022 12:19 AM (0): MSF Files: Added 120745 PSMs to result file.

10/13/2022 12:19 AM (0): MSF Files: Added 33401 Decoy PSMs to result file.

10/13/2022 12:19 AM (0): MSF Files: Added 156022 MS/MS Spectrum Info to result file.

10/13/2022 12:19 AM (0): MSF Files: Added 156022 Precursors to result file.

10/13/2022 12:19 AM (0): MSF Files: Added 6 Mass Recalibrations to result file.

10/13/2022 12:19 AM (0): MSF Files: Added 42440 Correction Landmarks to result file.

10/13/2022 12:19 AM (0): MSF Files: Added 18 Specialized Traces to result file.

10/13/2022 12:19 AM (0): MSF Files: Added 462149 LCMS Features to result file.

10/13/2022 12:20 AM (0): MSF Files: Added 2137456 LCMS Peaks to result file.

10/13/2022 12:25 AM(0): MSF Files: Copying data took 12 min 57 s.

10/13/2022 12:25 AM(0): MSF Files: Finding unique sequences took 16.9 s.

10/13/2022 12:26 AM(0): MSF Files: Filtering proteins took 9.5 s.

10/13/2022 12:28 AM(0): MSF Files: -- Total execution of MSF Files (0) took 15 min 57 s --

10/13/2022 12:28 AM(1): PSM Grouper: Grouping 'PSMs'

10/13/2022 12:31 AM(1): PSM Grouper: Found 34434 Peptide Groups.

10/13/2022 12:31 AM(1): PSM Grouper: Grouping 'Decoy PSMs'

10/13/2022 12:31 AM(1): PSM Grouper: Found 23199 Decoy Peptide Groups.

10/13/2022 12:32 AM(1): PSM Grouper: -- Total execution of PSM Grouper (1) took 3 min 56 s --

10/13/2022 12:32 AM(2): Peptide Validator: Start PSM and Peptide validation in 'Automatic (Control peptide level error rate if possible)' mode...

10/13/2022 12:32 AM(2): Peptide Validator: Updated PSM confidences in 3.5 s.

10/13/2022 12:32 AM(2): Peptide Validator: Use svm score for 'peptide' qvality run.

10/13/2022 12:32 AM(2): Peptide Validator: Updated peptide confidences using qvality in 27.6 s.

10/13/2022 12:32 AM(2): Peptide Validator: Calculating Mascot thresholds.

10/13/2022 12:32 AM(2): Peptide Validator: -- Total execution of Peptide Validator (2) took 34.4 s --

10/13/2022 12:32 AM(3): Peptide and Protein Filter: Filter Peptide Groups

10/13/2022 12:33 AM(3): Peptide and Protein Filter: Filter 8998/34434 Peptide Groups (25436 excluded) and 82025/120745 PSMs (38720 excluded) took 28.6 s

10/13/2022 12:33 AM(3): Peptide and Protein Filter: Filtering Proteins

10/13/2022 12:33 AM (3): Peptide and Protein Filter: Filter 4150/26049 Proteins (21899 excluded) took 9.9 s

10/13/2022 12:33 AM (3): Peptide and Protein Filter: Filter Decoy Peptide Groups

10/13/2022 12:33 AM (3): Peptide and Protein Filter: Filter 89/23199 Decoy Peptide Groups (23110 excluded) and 212/33401 Decoy PSMs (33189 excluded) took 8.4 s

10/13/2022 12:33 AM (3): Peptide and Protein Filter: Filtering Decoy Proteins

10/13/2022 12:33 AM (3): Peptide and Protein Filter: Filter 188/23668 Decoy Proteins (23480 excluded) took 3.4 s

10/13/2022 12:34 AM (3): Peptide and Protein Filter: Updating counts took 1 min 6 s.

10/13/2022 12:34 AM (3): Peptide and Protein Filter: -- Total execution of Peptide and Protein Filter (3) took 2 min 3 s --

10/13/2022 12:34 AM (4): Protein Scorer: Calculating Coverage and Counts

10/13/2022 12:35 AM (4): Protein Scorer: Calculated counts and coverages in 27.3 s.

10/13/2022 12:35 AM (4): Protein Scorer: Scoring target proteins

10/13/2022 12:36 AM (4): Protein Scorer: Scored 4150 proteins in 49.7 s.

10/13/2022 12:36 AM (4): Protein Scorer: Scoring decoy proteins

10/13/2022 12:36 AM (4): Protein Scorer: Scored 188 decoy proteins in 1.6 s.

10/13/2022 12:36 AM (4): Protein Scorer: -- Total execution of Protein Scorer (4) took 1 min 19 s --

10/13/2022 12:36 AM (5): Protein Grouping: Retrieving 1704 protein groups took 437.5 ms.

10/13/2022 12:37 AM (5): Protein Grouping: Storing, updating and connecting protein groups, PSMs and peptides took 45.8 s.

10/13/2022 12:37 AM (5): Protein Grouping: Check 1704 protein groups.

10/13/2022 12:37 AM (5): Protein Grouping: Applying strict parsimony took 37.9 s.

10/13/2022 12:37 AM(5): Protein Grouping:Found 1693 protein groups.

10/13/2022 12:37 AM(5): Protein Grouping:Retrieving 89 decoy protein groups took 13.2 ms.

10/13/2022 12:38 AM(5): Protein Grouping:Storing, updating and connecting protein groups, PSMs and peptides took 10.1 s.

10/13/2022 12:38 AM(5): Protein Grouping:Found 89 decoy protein groups.

10/13/2022 12:38 AM(5): Protein Grouping:-- Total execution of Protein Grouping (5) took 2 min 27 s --

10/13/2022 12:38 AM(6): Peptide in Protein Annotation: Start retrieving flanking residues and positions...

10/13/2022 12:43 AM(6): Peptide in Protein Annotation: Annotated PSMs/peptides for 4150 proteins.

10/13/2022 12:43 AM(6): Peptide in Protein Annotation: Start annotating peptide groups with flanking residues and positions...

10/13/2022 12:43 AM(6): Peptide in Protein Annotation: Annotate Peptide Groups items

10/13/2022 12:44 AM(6): Peptide in Protein Annotation: Start calculating protein coverage...

10/13/2022 12:44 AM(6): Peptide in Protein Annotation: -- Total execution of Peptide in Protein Annotation (6) took 6 min 18 s --

10/13/2022 12:44 AM(7): Protein FDR Validator: Apply confidences based on protein scores of 4150 proteins.

10/13/2022 12:44 AM(7): Protein FDR Validator: Calculating Sum PEP Score for Proteins

10/13/2022 12:45 AM(7): Protein FDR Validator: Calculating Sum PEP Score for Decoy Proteins

10/13/2022 12:45 AM(7): Protein FDR Validator: -- Total execution of Protein FDR Validator (7) took 40.7 s --

10/13/2022 12:45 AM(10): Feature Mapper: Extracting features

10/13/2022 12:46 AM(10): Feature Mapper: Extracting LCMS features

10/13/2022 12:48 AM(10): Feature Mapper: Extracting feature groups took 2 min 32 s.

10/13/2022 12:48 AM(10): Feature Mapper: Start aligning 3 files ('F3', 'F4', 'F5') to reference file 'F6'...

10/13/2022 12:48 AM(10): Feature Mapper: Start aligning 2 files ('F1', 'F2') to reference file 'F3'...

10/13/2022 12:48 AM(10): Feature Mapper: Calculating RT alignment took 2 min 46 s

10/13/2022 12:48 AM(10): Feature Mapper: Start updating RTs...

10/13/2022 12:48 AM(10): Feature Mapper: Updating RTs of LCMS Features took 14.5 s.

10/13/2022 12:50 AM(10): Feature Mapper: Updating RTs of LCMS Peaks took 1 min 24 s.

10/13/2022 12:50 AM(10): Feature Mapper: Updating RTs of PSMs took 10.3 s.

10/13/2022 12:50 AM(10): Feature Mapper: Updating RTs of MS/MS Spectrum Info took 5.2 s.

10/13/2022 12:50 AM(10): Feature Mapper: -- Performing retention time alignment took 4 min 41 s. --

10/13/2022 12:50 AM(10): Feature Mapper: Extracting feature groups took 0.3 ms.

10/13/2022 12:50 AM(10): Feature Mapper: Start aligning 3 files ('F3', 'F4', 'F5') to reference file 'F6'...

10/13/2022 12:50 AM(10): Feature Mapper: Start aligning 2 files ('F1', 'F2') to reference file 'F3'...

10/13/2022 12:50 AM(10): Feature Mapper: Updating m/z values of LCMS Features took 12.2 s.

10/13/2022 12:52 AM(10): Feature Mapper: Updating m/z values of LCMS Peaks took 1 min 12 s.

10/13/2022 12:52 AM(10): Feature Mapper: Updating m/z values of PSMs took 10.5 s.

10/13/2022 12:52 AM(10): Feature Mapper: -- Performing mass over charge alignment took 1 min 46 s. --

10/13/2022 12:52 AM(10): Feature Mapper: Extracting feature groups took 0.5 ms.

10/13/2022 12:52 AM(10): Feature Mapper: Start aligning 3 files ('F3', 'F4', 'F5') to reference file 'F6'...

10/13/2022 12:52 AM(10): Feature Mapper: Start aligning 2 files ('F1', 'F2') to reference file 'F3'...

10/13/2022 12:52 AM(10): Feature Mapper: Updating area values of LCMS Features took 14.4 s.

10/13/2022 12:54 AM(10): Feature Mapper: Updating area values of LCMS Peaks took 1 min 24 s.

10/13/2022 12:54 AM(10): Feature Mapper: -- Performing abundance alignment took 1 min 50 s. --

10/13/2022 12:54 AM(10): Feature Mapper: Using RT tolerance of 1.92 min and mass tolerance of 3.6 ppm for mapping

10/13/2022 12:54 AM(10): Feature Mapper: Mapping 1 Fraction(s)

10/13/2022 12:56 AM(10): Feature Mapper: Mapping Fraction 1 of 1

10/13/2022 01:09 AM(10): Feature Mapper: Mapping 1 Fraction(s) took 15 min 49 s

10/13/2022 01:09 AM(10): Feature Mapper: -- Performing cross file mapping took 15 min 49 s. --

10/13/2022 01:09 AM(10): Feature Mapper: Storing Top Apex RT for Peptide Groups

10/13/2022 01:10 AM(10): Feature Mapper: Storing Top Apex RT for Peptide Groups took 17.7 s

10/13/2022 01:10 AM(10): Feature Mapper: -- Total execution of Feature Mapper (10) took 24 min 42 s --

10/13/2022 01:10 AM(11): Precursor Ions Quantifier: Validating LCMS features

10/13/2022 01:10 AM(11): Precursor Ions Quantifier: Validating Consensus Features

10/13/2022 01:11 AM(11): Precursor Ions Quantifier: Validation of quantification results took 1 min 29 s

10/13/2022 01:11 AM(11): Precursor Ions Quantifier: Updating spectrum match areas

10/13/2022 01:11 AM(11): Precursor Ions Quantifier: Classify quan usage of PSMs

10/13/2022 01:12 AM(11): Precursor Ions Quantifier: Classifying Peptide Groups quan usage

10/13/2022 01:12 AM(11): Precursor Ions Quantifier: Classifying Peptide Groups quan usage for 8998 items took 11.1 s

10/13/2022 01:12 AM(11): Precursor Ions Quantifier: Calculating Peptide Group Abundances

10/13/2022 01:12 AM(11): Precursor Ions Quantifier: Classifying Protein Quan Usage

10/13/2022 01:12 AM(11): Precursor Ions Quantifier: Counting Razor Peptides

10/13/2022 01:12 AM(11): Precursor Ions Quantifier: Calculating Protein Abundances

10/13/2022 01:12 AM(11): Precursor Ions Quantifier: Start normalization of abundance values...

10/13/2022 01:13 AM(11): Precursor Ions Quantifier: Calculating normalization took 12.9 s

10/13/2022 01:13 AM (11): Precursor Ions Quantifier: Calculating Normalized Peptide Group Abundances

10/13/2022 01:13 AM (11): Precursor Ions Quantifier: Calculating Normalized Protein Abundances

10/13/2022 01:13 AM (11): Precursor Ions Quantifier: Calculating and applying normalization took 30.7 s

10/13/2022 01:13 AM (11): Precursor Ions Quantifier: Calculating ratios for Peptide Groups...

10/13/2022 01:13 AM (11): Precursor Ions Quantifier: Calculating ratios for Proteins...

10/13/2022 01:13 AM (11): Precursor Ions Quantifier: Start calculating p-values based on background for Peptide Groups...

10/13/2022 01:14 AM (11): Precursor Ions Quantifier: Calculating p-values based on background took 58.1 s

10/13/2022 01:14 AM (11): Precursor Ions Quantifier: Calculating p-values for Peptide Groups took 58.3 s.

10/13/2022 01:14 AM (11): Precursor Ions Quantifier: Start calculating p-values based on background for Proteins...

10/13/2022 01:14 AM (11): Precursor Ions Quantifier: Calculating p-values based on background took 7.5 s

10/13/2022 01:14 AM (11): Precursor Ions Quantifier: Calculating p-values for Proteins took 7.7 s.

10/13/2022 01:14 AM (11): Precursor Ions Quantifier: Precursor Ion Quantification took 4 min 36 s.

10/13/2022 01:15 AM (11): Precursor Ions Quantifier: Preparing spectrum visualization took 22 s.

10/13/2022 01:15 AM (11): Precursor Ions Quantifier: -- Total execution of Precursor Ions Quantifier (11) took 4 min 59 s --

10/13/2022 01:15 AM(12): Result Statistics: -- Total execution of Result Statistics (12) took 36.7 s --

10/13/2022 01:15 AM(13): Display Settings: Applying display filter and layout

10/13/2022 01:15 AM(13): Display Settings: -- Total execution of Display Settings (13) took 114 ms --

10/13/2022 01:15 AM(14): Data Distributions: Calculating found in files

10/13/2022 01:17 AM(14): Data Distributions: Calculating found for samples

10/13/2022 01:18 AM(14): Data Distributions: Calculating found for sample groups

10/13/2022 01:20 AM(14): Data Distributions: -- Total execution of Data Distributions (14) took 4 min 35 s --

10/13/2022 01:22 AM Job Execution: Finalizing file took 1 min 43 s.

10/13/2022 01:22 AM Job Execution: Finished V:\Chung Group\Livia\RawFiles\10_2022\Deivid\ALI_Oct2022\ALI_Oct2022\ALI_Oct2022.pdResult

10/13/2022 01:22 AM Job Execution: ----- Total Job execution took: 1 h 9 min. -----

==

Processing Step A: Workflow

==

Result name: ALI_Oct2022

Result file: V:\Chung Group\Livia\RawFiles\10_2022\Deivid\ALI_Oct2022\ALI_Oct2022\ALI_Oct2022.msf

Description: Processing workflow for precursor-based quantification. CID spectra using SequestHT with Percolator validation. Specify the FASTA database, labels used, and any additional modifications.

Workflow based on template: PWF_OT_Precursor_Quan_and_LFQ_CID_SequestHT_Percolator

Creation date: 10/12/2022 10:27:12 PM

Created with Discoverer version: 2.4.1.15

The workflow tree:

```
 |-(0) Spectrum Files RC
   |-(1) Spectrum Selector
     |-(2) Sequest HT
       |-(3) Percolator
   |-(4) Minora Feature Detector
```

Processing node 0: Spectrum Files RC

----

1. Search Settings:

- File Name(s) (Hidden):

V:\Chung Group\Livia\RawFiles\10_2022\Deivid\A1.raw

V:\Chung Group\Livia\RawFiles\10_2022\Deivid\A2.raw

V:\Chung Group\Livia\RawFiles\10_2022\Deivid\A3.raw

V:\Chung Group\Livia\RawFiles\10_2022\Deivid\C1.raw

V:\Chung Group\Livia\RawFiles\10_2022\Deivid\C2.raw

V:\Chung Group\Livia\RawFiles\10_2022\Deivid\C3.raw

- Protein Database: Homo sapiens (SwissProt TaxID=9606) (v2017-10-25)

- Enzyme Name: Trypsin (Full)

- Precursor Mass Tolerance: 20 ppm

- Fragment Mass Tolerance: 0.5 Da

- 1. Static Modification: Carbamidomethyl / +57.021 Da (C)

2. Regression Settings:

- Regression Model: Non-linear Regression

- Parameter Tuning: Coarse

Processing node 1: Spectrum Selector

----

1. General Settings:

- Precursor Selection: Use MS1 Precursor

- Use Isotope Pattern in Precursor Reevaluation: True

- Provide Profile Spectra: Automatic

2. Spectrum Properties Filter:

- Lower RT Limit: 0

- Upper RT Limit: 0

- First Scan: 0

- Last Scan: 0

- Lowest Charge State: 0

- Highest Charge State: 0

- Min. Precursor Mass: 350 Da

- Max. Precursor Mass: 5000 Da

- Total Intensity Threshold: 0

- Minimum Peak Count: 1

3. Scan Event Filters:

- MS Order: Is Not MS1

- Min. Collision Energy: 0

- Max. Collision Energy: 1000

- Scan Type: Is Full

4. Peak Filters:

- S/N Threshold (FT-only): 1.5

5. Replacements for Unrecognized Properties:

- Unrecognized Charge Replacements: Automatic

- Unrecognized Mass Analyzer Replacements: ITMS

- Unrecognized MS Order Replacements: MS2

- Unrecognized Activation Type Replacements: CID

- Unrecognized Polarity Replacements: +

- Unrecognized MS Resolution@200 Replacements: 60000

- Unrecognized MSn Resolution@200 Replacements: 30000

6. Precursor Pattern Extraction:

- Precursor Clipping Range Before: 2.5 Da

- Precursor Clipping Range After: 5.5 Da

Processing node 2: Sequest HT

----

1. Input Data:

- Protein Database: Homo sapiens (SwissProt TaxID=9606) (v2017-10-25)

- Enzyme Name: Trypsin (Full)

- Max. Missed Cleavage Sites: 2

- Min. Peptide Length: 6

- Max. Peptide Length: 144

- Max. Number of Peptides Reported: 10

2. Tolerances:

- Precursor Mass Tolerance: 10 ppm

- Fragment Mass Tolerance: 0.6 Da

- Use Average Precursor Mass: False

- Use Average Fragment Mass: False

3. Spectrum Matching:

- Use Neutral Loss a Ions: True

- Use Neutral Loss b Ions: True

- Use Neutral Loss y Ions: True

- Use Flanking Ions: True

- Weight of a Ions: 0

- Weight of b Ions: 1

- Weight of c Ions: 0

- Weight of x Ions: 0

- Weight of y Ions: 1

- Weight of z Ions: 0

4. Dynamic Modifications:

- Max. Equal Modifications Per Peptide: 3

- Max. Dynamic Modifications Per Peptide: 4

- 1. Dynamic Modification: Oxidation / +15.995 Da (M)

6. Dynamic Modifications (protein terminus):

- 1. N-Terminal Modification: Acetyl / +42.011 Da (N-Terminus)

- 2. N-Terminal Modification: Met-loss / -131.040 Da (M)

- 3. N-Terminal Modification: Met-loss+Acetyl / -89.030 Da (M)

7. Static Modifications:

- 1. Static Modification: Carbamidomethyl / +57.021 Da (C)

Processing node 3: Percolator

----

1. Target/Decoy Strategy:

- Target/Decoy Selection: Concatenated

- Validation based on: q-Value

2. Input Data:

- Maximum Delta Cn: 0.05

- Maximum Rank: 0

3. FDR Targets:

- Target FDR (Strict): 0.01

- Target FDR (Relaxed): 0.05

Processing node 4: Minora Feature Detector

----

1. Peak & Feature Detection:

- Min. Trace Length: 5

- Max. ΔRT of Isotope Pattern Multiplets [min]: 0.2

2. Feature to ID Linking:

- PSM Confidence At Least: High

Workflow messages:

10/12/2022 10:27 PM Job Execution:	Processing V:\Chung Group\Livia\RawFiles\10_2022\Deivid\ALI_Oct2022\ALI_Oct2022\ALI_Oct2022.msf

10/12/2022 10:27 PM (0): Spectrum Files RC:	Start processing file F1: A1.raw...

10/12/2022 10:28 PM (0): Spectrum Files RC:	Retrieving 27304 spectra took 1 min 33 s

10/12/2022 10:28 PM (0): Spectrum Files RC:	There is already an adequate target FASTA index for ProteinCenter_9606_SwissProt.fasta.

10/12/2022 10:28 PM (0): Spectrum Files RC:	Start searching spectra (CID (Collision Induced Dissociation))...

10/12/2022 10:28 PM (0): Spectrum Files RC:	ISE (1.1.0.189, x64) started at 10/12/2022 10:28:57 PM on MEDCHUNGDW457 (x64) [16 CPUs] running Microsoft Windows NT 6.2.9200.0 (64bit) [.NET: 4.0.30319.42000]

10/12/2022 10:28 PM (0): Spectrum Files RC:	Workload level: #parallel tasks: 10

10/12/2022 10:28 PM (0): Spectrum Files RC:	Workload level: #spectra loaded and processed at once: 10000

10/12/2022 10:28 PM (0): Spectrum Files RC:	On-Disk search is performed

10/12/2022 10:30 PM (0): Spectrum Files RC:	Average search time per spectrum was 2.6 ms.

10/12/2022 10:30 PM(0): Spectrum Files RC: Start reading spectrum results...

10/12/2022 10:30 PM(0): Spectrum Files RC: Start calculating calibration...

10/12/2022 10:30 PM(0): Spectrum Files RC: Processing file F1 took 2 min 55 s.

10/12/2022 10:30 PM(0): Spectrum Files RC: Start processing file F2: A2.raw...

10/12/2022 10:31 PM(0): Spectrum Files RC: Retrieving 27584 spectra took 1 min 38 s

10/12/2022 10:31 PM(0): Spectrum Files RC: Start searching spectra (CID (Collision Induced Dissociation))...

10/12/2022 10:31 PM(0): Spectrum Files RC: ISE (1.1.0.189, x64) started at 10/12/2022 10:31:58 PM on MEDCHUNGDW457 (x64) [16 CPUs] running Microsoft Windows NT 6.2.9200.0 (64bit) [.NET: 4.0.30319.42000]

10/12/2022 10:31 PM(0): Spectrum Files RC: Workload level: #parallel tasks: 10

10/12/2022 10:31 PM(0): Spectrum Files RC: Workload level: #spectra loaded and processed at once: 10000

10/12/2022 10:31 PM(0): Spectrum Files RC: On-Disk search is performed

10/12/2022 10:33 PM(0): Spectrum Files RC: Average search time per spectrum was 2.5 ms.

10/12/2022 10:33 PM(0): Spectrum Files RC: Start reading spectrum results...

10/12/2022 10:33 PM(0): Spectrum Files RC: Start calculating calibration...

10/12/2022 10:33 PM(0): Spectrum Files RC: Processing file F2 took 3 min 0 s.

10/12/2022 10:33 PM(0): Spectrum Files RC: Start processing file F3: A3.raw...

10/12/2022 10:34 PM(0): Spectrum Files RC: Retrieving 27513 spectra took 1 min 35 s

10/12/2022 10:34 PM(0): Spectrum Files RC: Start searching spectra (CID (Collision Induced Dissociation))...

10/12/2022 10:34 PM(0): Spectrum Files RC: ISE (1.1.0.189, x64) started at 10/12/2022 10:34:55 PM on MEDCHUNGDW457 (x64) [16 CPUs] running Microsoft Windows NT 6.2.9200.0 (64bit) [.NET: 4.0.30319.42000]

10/12/2022 10:34 PM(0): Spectrum Files RC: Workload level: #parallel tasks: 10

10/12/2022 10:34 PM(0): Spectrum Files RC: Workload level: #spectra loaded and processed at once: 10000

10/12/2022 10:34 PM(0): Spectrum Files RC: On-Disk search is performed

10/12/2022 10:36 PM(0): Spectrum Files RC: Average search time per spectrum was 2.5 ms.

10/12/2022 10:36 PM(0): Spectrum Files RC: Start reading spectrum results...

10/12/2022 10:36 PM(0): Spectrum Files RC: Start calculating calibration...

10/12/2022 10:36 PM(0): Spectrum Files RC: Processing file F3 took 2 min 54 s.

10/12/2022 10:36 PM(0): Spectrum Files RC: Start processing file F4: C1.raw...

10/12/2022 10:37 PM(0): Spectrum Files RC: Retrieving 26868 spectra took 1 min 28 s

10/12/2022 10:37 PM(0): Spectrum Files RC: Start searching spectra (CID (Collision Induced Dissociation))...

10/12/2022 10:37 PM(0): Spectrum Files RC: ISE (1.1.0.189, x64) started at 10/12/2022 10:37:42 PM on MEDCHUNGDW457 (x64) [16 CPUs] running Microsoft Windows NT 6.2.9200.0 (64bit) [.NET: 4.0.30319.42000]

10/12/2022 10:37 PM(0): Spectrum Files RC: Workload level: #parallel tasks: 10

10/12/2022 10:37 PM(0): Spectrum Files RC: Workload level: #spectra loaded and processed at once: 10000

10/12/2022 10:37 PM(0): Spectrum Files RC: On-Disk search is performed

10/12/2022 10:38 PM(0): Spectrum Files RC: Average search time per spectrum was 2.4 ms.

10/12/2022 10:38 PM(0): Spectrum Files RC: Start reading spectrum results...

10/12/2022 10:38 PM(0): Spectrum Files RC: Start calculating calibration...

10/12/2022 10:38 PM(0): Spectrum Files RC: Processing file F4 took 2 min 43 s.

10/12/2022 10:38 PM(0): Spectrum Files RC: Start processing file F5: C2.raw...

10/12/2022 10:40 PM(0): Spectrum Files RC: Retrieving 27601 spectra took 1 min 34 s

10/12/2022 10:40 PM(0): Spectrum Files RC: Start searching spectra (CID (Collision Induced Dissociation))...

10/12/2022 10:40 PM(0): Spectrum Files RC: ISE (1.1.0.189, x64) started at 10/12/2022 10:40:32 PM on MEDCHUNGDW457 (x64) [16 CPUs] running Microsoft Windows NT 6.2.9200.0 (64bit) [.NET: 4.0.30319.42000]

10/12/2022 10:40 PM(0): Spectrum Files RC: Workload level: #parallel tasks: 10

10/12/2022 10:40 PM(0): Spectrum Files RC: Workload level: #spectra loaded and processed at once: 10000

10/12/2022 10:40 PM(0): Spectrum Files RC: On-Disk search is performed

10/12/2022 10:41 PM(0): Spectrum Files RC: Average search time per spectrum was 2.5 ms.

10/12/2022 10:41 PM(0): Spectrum Files RC: Start reading spectrum results...

10/12/2022 10:41 PM(0): Spectrum Files RC: Start calculating calibration...

10/12/2022 10:41 PM(0): Spectrum Files RC: Processing file F5 took 2 min 53 s.

10/12/2022 10:41 PM(0): Spectrum Files RC: Start processing file F6: C3.raw...

10/12/2022 10:43 PM(0): Spectrum Files RC: Retrieving 27765 spectra took 1 min 28 s

10/12/2022 10:43 PM(0): Spectrum Files RC: Start searching spectra (CID (Collision Induced Dissociation))...

10/12/2022 10:43 PM(0): Spectrum Files RC: ISE (1.1.0.189, x64) started at 10/12/2022 10:43:19 PM on MEDCHUNGDW457 (x64) [16 CPUs] running Microsoft Windows NT 6.2.9200.0 (64bit) [.NET: 4.0.30319.42000]

10/12/2022 10:43 PM(0): Spectrum Files RC: Workload level: #parallel tasks: 10

10/12/2022 10:43 PM(0): Spectrum Files RC: Workload level: #spectra loaded and processed at once: 10000

10/12/2022 10:43 PM(0): Spectrum Files RC: On-Disk search is performed

10/12/2022 10:44 PM(0): Spectrum Files RC: Average search time per spectrum was 2.4 ms.

10/12/2022 10:44 PM(0): Spectrum Files RC: Start reading spectrum results...

10/12/2022 10:44 PM(0): Spectrum Files RC: Start calculating calibration...

10/12/2022 10:44 PM(0): Spectrum Files RC: Processing file F6 took 2 min 45 s.

10/12/2022 10:44 PM(0): Spectrum Files RC: -- Total execution of Spectrum Files RC (0) took 17 min 13 s --

10/12/2022 10:44 PM(1): Spectrum Selector: Profile spectra are not sent.

10/12/2022 10:44 PM(1): Spectrum Selector: Reading from file 1 of 6 F1: V:\Chung Group\Livia\RawFiles\10_2022\Deivid\A1.raw (29393 spectra total)

10/12/2022 10:44 PM(2): Sequest HT: Sequence Database: Homo sapiens (SwissProt TaxID=9606) Version: 2017-10-25

10/12/2022 10:48 PM(1): Spectrum Selector: Sent 26137 spectra from file F1.

10/12/2022 10:48 PM(1): Spectrum Selector: Reading from file 2 of 6 F2: V:\Chung Group\Livia\RawFiles\10_2022\Deivid\A2.raw (29646 spectra total)

10/12/2022 10:52 PM(1): Spectrum Selector: Sent 26013 spectra from file F2.

10/12/2022 10:52 PM(1): Spectrum Selector: Reading from file 3 of 6 F3: V:\Chung Group\Livia\RawFiles\10_2022\Deivid\A3.raw (29583 spectra total)

10/12/2022 10:56 PM(1): Spectrum Selector: Sent 26338 spectra from file F3.

10/12/2022 10:56 PM(1): Spectrum Selector: Reading from file 4 of 6 F4: V:\Chung Group\Livia\RawFiles\10_2022\Deivid\C1.raw (28970 spectra total)

10/12/2022 10:59 PM(1): Spectrum Selector: Sent 25726 spectra from file F4.

10/12/2022 10:59 PM (1): Spectrum Selector: Reading from file 5 of 6 F5: V:\Chung Group\Livia\RawFiles\10_2022\Deivid\C2.raw (29642 spectra total)

10/12/2022 11:03 PM (1): Spectrum Selector: Sent 25516 spectra from file F5.

10/12/2022 11:03 PM (1): Spectrum Selector: Reading from file 6 of 6 F6: V:\Chung Group\Livia\RawFiles\10_2022\Deivid\C3.raw (29810 spectra total)

10/12/2022 11:07 PM (1): Spectrum Selector: Sent 26292 spectra from file F6.

10/12/2022 11:07 PM (1): Spectrum Selector: Sent 156022 spectra from 6 files (processing time: 6 min 25 s).

10/12/2022 11:07 PM (1): Spectrum Selector: -- Total execution of Spectrum Selector (1) took 22 min 45 s --

10/12/2022 11:07 PM (2): Sequest HT: Storing spectra took 15 min 35 s.

10/12/2022 11:07 PM (2): Sequest HT: There is already an adequate target FASTA index for ProteinCenter_9606_SwissProt.fasta.

10/12/2022 11:07 PM (2): Sequest HT: Start Sequest HT target search for 156022 spectra (156022 precursors)...

10/12/2022 11:07 PM (2): Sequest HT: ISE (1.1.0.189, x64) started at 10/12/2022 11:07:22 PM on MEDCHUNGDW457 (x64) [16 CPUs] running Microsoft Windows NT 6.2.9200.0 (64bit) [.NET: 4.0.30319.42000]

10/12/2022 11:07 PM (2): Sequest HT: Workload level: #parallel tasks: 10

10/12/2022 11:07 PM (2): Sequest HT: Workload level: #spectra loaded and processed at once: 10000

10/12/2022 11:07 PM (2): Sequest HT: On-Disk search is performed

10/12/2022 11:14 PM (2): Sequest HT: Average search time per spectrum was 2.6 ms.

10/12/2022 11:14 PM (2): Sequest HT: Performing target search took 6 min 50 s.

10/12/2022 11:16 PM (2): Sequest HT: Stored 190448 PSMs for 156022 spectra

10/12/2022 11:16 PM(2): Sequest HT: Discarded 628659 peptide(s) that did not match the conditions for protein terminal modifications.

10/12/2022 11:16 PM(2): Sequest HT: Reading search results took 42 s.

10/12/2022 11:16 PM(2): Sequest HT: Saving results took 1 min 17 s.

10/12/2022 11:16 PM(2): Sequest HT: Saving proteins took 34.6 s.

10/12/2022 11:16 PM(2): Sequest HT: Reading results took 2 min 37 s.

10/12/2022 11:16 PM(2): Sequest HT: There is already an adequate decoy FASTA index for ProteinCenter_9606_SwissProt_reversed.fasta.

10/12/2022 11:16 PM(2): Sequest HT: Start Sequest HT decoy search for 156022 spectra (156022 precursors)...

10/12/2022 11:16 PM(2): Sequest HT: ISE (1.1.0.189, x64) started at 10/12/2022 11:16:50 PM on MEDCHUNGDW457 (x64) [16 CPUs] running Microsoft Windows NT 6.2.9200.0 (64bit) [.NET: 4.0.30319.42000]

10/12/2022 11:16 PM(2): Sequest HT: Workload level: #parallel tasks: 10

10/12/2022 11:16 PM(2): Sequest HT: Workload level: #spectra loaded and processed at once: 10000

10/12/2022 11:16 PM(2): Sequest HT: On-Disk search is performed

10/12/2022 11:23 PM(2): Sequest HT: Average search time per spectrum was 2.6 ms.

10/12/2022 11:23 PM(2): Sequest HT: Performing decoy search took 6 min 52 s.

10/12/2022 11:26 PM(2): Sequest HT: Stored 417585 decoy PSMs for 156022 spectra

10/12/2022 11:26 PM(2): Sequest HT: Discarded 695496 peptide(s) that did not match the conditions for protein terminal modifications.

10/12/2022 11:26 PM(2): Sequest HT: Reading search results took 49.3 s.

10/12/2022 11:26 PM(2): Sequest HT: Saving results took 1 min 31 s.

10/12/2022 11:26 PM(2): Sequest HT: Saving proteins took 37.9 s.

10/12/2022 11:26 PM(2): Sequest HT: Reading results took 2 min 59 s.

10/12/2022 11:26 PM(2): Sequest HT: Finalizing search results...

10/12/2022 11:32 PM(2): Sequest HT: -- Total search time was 19 min 47 s --

10/12/2022 11:46 PM(3): Percolator: Creating input file for Sequest HT (2) took 14 min 38 s.

10/12/2022 11:46 PM(3): Percolator: The input file contains 120745 PSMs, 33401 decoy PSMs and 32 features.

10/12/2022 11:46 PM(3): Percolator: Generated percolator input for concatenated mode

10/12/2022 11:46 PM(3): Percolator: Percolator version 3.02.1, Build Date Aug 14 2018 00:44:01

10/12/2022 11:46 PM(3): Percolator: Copyright (c) 2006-9 University of Washington. All rights reserved.

10/12/2022 11:46 PM(3): Percolator: Written by Lukas K├ñll (lukall@u.washington.edu) in the

10/12/2022 11:46 PM(3): Percolator: Department of Genome Sciences at the University of Washington.

10/12/2022 11:46 PM(3): Percolator: Issued command:

10/12/2022 11:46 PM(3): Percolator: C:\Program Files\Thermo\Proteome Discoverer 2.4\Tools\Percolator\percolator.exe -s -X C:\ProgramData\Thermo\Proteome Discoverer 2.4\Scratch\Job804\Percol(3)\output2.xml -Z -I auto -k C:\ProgramData\Thermo\Proteome Discoverer 2.4\Scratch\Job804\Percol(3)\input2.xml

10/12/2022 11:46 PM(3): Percolator: Started Wed Oct 12 23:46:48 2022

10/12/2022 11:46 PM(3): Percolator: Hyperparameters: selectionFdr=0.01, Cpos=0, Cneg=0, maxNiter=10

10/12/2022 11:46 PM (3): Percolator: Reading pin-xml input from datafile C:\ProgramData\Thermo\Proteome Discoverer 2.4\Scratch\Job804\Percol(3)\input2.xml

10/12/2022 11:46 PM (3): Percolator: enzyme=Trypsin

10/12/2022 11:46 PM (3): Percolator: Features:

10/12/2022 11:46 PM (3): Percolator: XCorr Delta Cn From Second PSM Binomial Score Isolation Interference [%] MH+ [Da] Delta Mass [Da] Delta Mass [ppm] Absolute Delta Mass [Da] Absolute Delta Mass [ppm] Peptide Length Is z=1 Is z=2 Is z=3 Is z=4 Is z=5 Is z>5 # Missed Cleavages Log Peptides Matched Log Total Intensity Fraction Matched Intensity [%] Fragment Coverage Series A, B, C [%] Fragment Coverage Series X, Y, Z [%] Log Matched Fragment Series Intensities A, B, C Log Matched Fragment Series Intensities X, Y, Z Longest Sequence Series A, B, C Longest Sequence Series X, Y, Z IQR Fragment Delta Mass [Da] IQR Fragment Delta Mass [ppm] Mean Fragment Delta Mass [Da] Mean Fragment Delta Mass [ppm] Mean Absolute Fragment Delta Mass [Da] Mean Absolute Fragment Delta Mass [ppm]

10/12/2022 11:47 PM (3): Percolator: Concatenated search input detected, skipping both target-decoy competition and mix-max.

10/12/2022 11:47 PM (3): Percolator: Train/test set contains 120745 positives and 33401 negatives, size ratio=3.61501 and pi0=1

10/12/2022 11:47 PM (3): Percolator: Selecting Cpos by cross-validation.

10/12/2022 11:47 PM (3): Percolator: Selecting Cneg by cross-validation.

10/12/2022 11:47 PM (3): Percolator: Split 1: Selected feature 3 as initial direction. Could separate 50232 training set positives with q<0.01 in that direction.

10/12/2022 11:47 PM (3): Percolator: Split 2: Selected feature 3 as initial direction. Could separate 50086 training set positives with q<0.01 in that direction.

10/12/2022 11:47 PM (3): Percolator: Split 3: Selected feature 3 as initial direction. Could separate 49938 training set positives with q<0.01 in that direction.

10/12/2022 11:47 PM (3): Percolator: Found 75012 test set positives with q<0.01 in initial direction

10/12/2022 11:47 PM (3): Percolator: Reading in data and feature calculation took 34.51 cpu seconds or 34 seconds wall clock time.

10/12/2022 11:47 PM (3): Percolator: ---Training with Cpos selected by cross validation, Cneg selected by cross validation, initial_fdr=0.01, fdr=0.01

10/12/2022 11:47 PM (3): Percolator: Iteration 1: Estimated 83266 PSMs with q<0.01

10/12/2022 11:47 PM (3): Percolator: Iteration 2: Estimated 83810 PSMs with q<0.01

10/12/2022 11:47 PM (3): Percolator: Iteration 3: Estimated 83956 PSMs with q<0.01

10/12/2022 11:47 PM (3): Percolator: Iteration 4: Estimated 83963 PSMs with q<0.01

10/12/2022 11:47 PM (3): Percolator: Iteration 5: Estimated 83971 PSMs with q<0.01

10/12/2022 11:47 PM (3): Percolator: Iteration 6: Estimated 83970 PSMs with q<0.01

10/12/2022 11:48 PM (3): Percolator: Iteration 7: Estimated 83968 PSMs with q<0.01

10/12/2022 11:48 PM (3): Percolator: Iteration 8: Estimated 83967 PSMs with q<0.01

10/12/2022 11:48 PM (3): Percolator: Iteration 9: Estimated 83972 PSMs with q<0.01

10/12/2022 11:48 PM (3): Percolator: Iteration 10: Estimated 83977 PSMs with q<0.01

10/12/2022 11:48 PM (3): Percolator: Learned normalized SVM weights for the 3 cross-validation splits:

10/12/2022 11:48 PM (3): Percolator: Split1 Split2 Split3 FeatureName

10/12/2022 11:48 PM(3): Percolator: XCorr	0.4830	0.4659	0.4078
10/12/2022 11:48 PM(3): Percolator: Delta Cn From Second PSM	0.0618	0.0829	0.0723
10/12/2022 11:48 PM(3): Percolator: Binomial Score	2.3140	2.1834	2.4532
10/12/2022 11:48 PM(3): Percolator: Isolation Interference [%]	0.1130	0.0760	0.1424
10/12/2022 11:48 PM(3): Percolator: MH+ [Da]	0.1928	0.3107	0.3705
10/12/2022 11:48 PM(3): Percolator: Delta Mass [Da]	0.3533	0.2419	0.1039
10/12/2022 11:48 PM(3): Percolator: Delta Mass [ppm]	-0.4922	-0.3717	-0.2214
10/12/2022 11:48 PM(3): Percolator: Absolute Delta Mass [Da]	0.2299	0.0284	0.2424
10/12/2022 11:48 PM(3): Percolator: Absolute Delta Mass [ppm]	-1.4769	-1.2884	-1.4878
10/12/2022 11:48 PM(3): Percolator: Peptide Length	-0.1687	-0.3000	-0.3585
10/12/2022 11:48 PM(3): Percolator: Is z=1	0.0000	0.0000	0.0000
10/12/2022 11:48 PM(3): Percolator: Is z=2	-0.0593	0.0958	0.1767
10/12/2022 11:48 PM(3): Percolator: Is z=3	0.1044	0.0942	0.0910
10/12/2022 11:48 PM(3): Percolator: Is z=4	0.0036	-0.1854	-0.2701

10/12/2022 11:48 PM(3): Percolator: Is z=5	-0.0688	-0.1483	-0.2337
10/12/2022 11:48 PM(3): Percolator: Is z>5	-0.1656	-0.2795	-0.3320
10/12/2022 11:48 PM(3): Percolator: # Missed Cleavages	-0.2976	-0.2767	-0.3237
10/12/2022 11:48 PM(3): Percolator: Log Peptides Matched	-0.0008	-0.0133	-0.0098
10/12/2022 11:48 PM(3): Percolator: Log Total Intensity	-0.1999	-0.2342	-0.2867
10/12/2022 11:48 PM(3): Percolator: Fraction Matched Intensity [%]	0.3568	0.3272	0.3375
10/12/2022 11:48 PM(3): Percolator: Fragment Coverage Series A, B, C [%]	-0.4963	-0.5741	-0.6159
10/12/2022 11:48 PM(3): Percolator: Fragment Coverage Series X, Y, Z [%]	-0.4166	-0.4313	-0.4209
10/12/2022 11:48 PM(3): Percolator: Log Matched Fragment Series Intensities A, B, C	-1.1780	-0.9453	-0.9005
10/12/2022 11:48 PM(3): Percolator: Log Matched Fragment Series Intensities X, Y, Z	1.3073	1.3279	1.5062
10/12/2022 11:48 PM(3): Percolator: Longest Sequence Series A, B, C	0.2019	0.2620	0.2318
10/12/2022 11:48 PM(3): Percolator: Longest Sequence Series X, Y, Z	0.8225	0.7702	0.8309
10/12/2022 11:48 PM(3): Percolator: IQR Fragment Delta Mass [Da]	-0.1186	0.0240	-0.0228
10/12/2022 11:48 PM(3): Percolator: IQR Fragment Delta Mass [ppm]	-0.1612	-0.2504	-0.1129

10/12/2022 11:48 PM (3): Percolator: -0.5338 -0.5393 -0.6068
Mean Fragment Delta Mass [Da]

10/12/2022 11:48 PM (3): Percolator: 0.1484 0.1229 0.1805
Mean Fragment Delta Mass [ppm]

10/12/2022 11:48 PM (3): Percolator: -0.4532 -0.5200 -0.5763
Mean Absolute Fragment Delta Mass [Da]

10/12/2022 11:48 PM (3): Percolator: -0.0774 -0.0671 -0.1176
Mean Absolute Fragment Delta Mass [ppm]

10/12/2022 11:48 PM (3): Percolator: 0.7006 0.2864 0.7235
m0

10/12/2022 11:48 PM (3): Percolator: Found 83851 test set PSMs with q<0.01.

10/12/2022 11:48 PM (3): Percolator: Tossing out "redundant" PSMs keeping only the best scoring PSM for each unique peptide.

10/12/2022 11:48 PM (3): Percolator: Calculating q values.

10/12/2022 11:48 PM (3): Percolator: Final list yields 8901 target peptides with q<0.01.

10/12/2022 11:48 PM (3): Percolator: Calculating posterior error probabilities (PEPs).

10/12/2022 11:49 PM (3): Percolator: Processing took 99.17 cpu seconds or 100 seconds wall clock time.

10/12/2022 11:49 PM (3): Percolator: PSMId score q-value
posterior_error_prob peptide proteinIds

10/12/2022 11:50 PM (3): Percolator: 83853/837 high confident target/decoy peptides were found for Sequest HT (2).

10/12/2022 11:50 PM (3): Percolator: 6134/3660 medium confident target/decoy peptides were found for Sequest HT (2).

10/12/2022 11:50 PM (3): Percolator: -- Total execution of Percolator (64Bit) for Sequest HT (2) took 18 min 27 s --

10/12/2022 11:50 PM(4): Minora Feature Detector: Start detecting lcmspeaks...

10/12/2022 11:50 PM(4): Minora Feature Detector: Running 6 jobs with 4 jobs in parallel.

10/12/2022 11:50 PM(4): Minora Feature Detector: Start F5 with 'FTMS + p NSI Full ms [350.00-1500.00]'

10/12/2022 11:50 PM(4): Minora Feature Detector: Start F1 with 'FTMS + p NSI Full ms [350.00-1500.00]'

10/12/2022 11:50 PM(4): Minora Feature Detector: Start F6 with 'FTMS + p NSI Full ms [350.00-1500.00]'

10/12/2022 11:50 PM(4): Minora Feature Detector: Start F3 with 'FTMS + p NSI Full ms [350.00-1500.00]'

10/12/2022 11:56 PM(4): Minora Feature Detector: Start F2 with 'FTMS + p NSI Full ms [350.00-1500.00]'

10/12/2022 11:56 PM(4): Minora Feature Detector: Start F4 with 'FTMS + p NSI Full ms [350.00-1500.00]'

10/13/2022 12:01 AM(4): Minora Feature Detector: Detecting LcmsPeaks took 10 min 43 s

10/13/2022 12:01 AM(4): Minora Feature Detector: Start processing F1: 'A1.raw'.

10/13/2022 12:01 AM(4): Minora Feature Detector: Start processing F2: 'A2.raw'.

10/13/2022 12:01 AM(4): Minora Feature Detector: Start processing F3: 'A3.raw'.

10/13/2022 12:01 AM(4): Minora Feature Detector: Start processing F4: 'C1.raw'.

10/13/2022 12:01 AM(4): Minora Feature Detector: Importing peaks for F1 (scan filter: FTMS + p NSI Full ms [350.00-1500.00])...

10/13/2022 12:01 AM(4): Minora Feature Detector: Importing peaks for F2 (scan filter: FTMS + p NSI Full ms [350.00-1500.00])...

10/13/2022 12:01 AM(4): Minora Feature Detector: Importing peaks for F4 (scan filter: FTMS + p NSI Full ms [350.00-1500.00])...

10/13/2022 12:01 AM(4): Minora Feature Detector: Importing peaks for F3 (scan filter: FTMS + p NSI Full ms [350.00-1500.00])...

10/13/2022 12:01 AM(4): Minora Feature Detector: F2: Found 357316 chromatographic peaks.

10/13/2022 12:01 AM(4): Minora Feature Detector: F1: Found 352436 chromatographic peaks.

10/13/2022 12:01 AM(4): Minora Feature Detector: F4: Found 348025 chromatographic peaks.

10/13/2022 12:01 AM(4): Minora Feature Detector: F3: Found 362793 chromatographic peaks.

10/13/2022 12:01 AM(4): Minora Feature Detector: F2: Importing auto traces...

10/13/2022 12:01 AM(4): Minora Feature Detector: F2: Imported 285034 auto traces.

10/13/2022 12:01 AM(4): Minora Feature Detector: F1: Importing auto traces...

10/13/2022 12:02 AM(4): Minora Feature Detector: F1: Imported 281509 auto traces.

10/13/2022 12:02 AM(4): Minora Feature Detector: F4: Importing auto traces...

10/13/2022 12:02 AM(4): Minora Feature Detector: F4: Imported 280652 auto traces.

10/13/2022 12:02 AM(4): Minora Feature Detector: F3: Importing auto traces...

10/13/2022 12:02 AM(4): Minora Feature Detector: F3: Imported 290673 auto traces.

10/13/2022 12:02 AM(4): Minora Feature Detector: Importing peak to spectrum links...

10/13/2022 12:05 AM (4): Minora Feature Detector: Found 21058 features (F3: 4993, F1: 5092, F2: 4816, F4: 6157)

10/13/2022 12:05 AM (4): Minora Feature Detector: Writing data took 18 s.

10/13/2022 12:05 AM (4): Minora Feature Detector: Creating features for PSMs took 30.5 s.

10/13/2022 12:07 AM (4): Minora Feature Detector: F4: Unidentified feature detection took 1 min 37 s

10/13/2022 12:07 AM (4): Minora Feature Detector: F1: Unidentified feature detection took 1 min 38 s

10/13/2022 12:07 AM (4): Minora Feature Detector: F2: Unidentified feature detection took 1 min 41 s

10/13/2022 12:07 AM (4): Minora Feature Detector: F3: Unidentified feature detection took 1 min 42 s

10/13/2022 12:07 AM (4): Minora Feature Detector: Found 285809 unidentified features (F4 : 70280, F1 : 71072, F2 : 71514, F3 : 72943).

10/13/2022 12:07 AM (4): Minora Feature Detector: Start processing F5: 'C2.raw'.

10/13/2022 12:07 AM (4): Minora Feature Detector: Start processing F6: 'C3.raw'.

10/13/2022 12:07 AM (4): Minora Feature Detector: Importing peaks for F5 (scan filter: FTMS + p NSI Full ms [350.00-1500.00])...

10/13/2022 12:07 AM (4): Minora Feature Detector: Importing peaks for F6 (scan filter: FTMS + p NSI Full ms [350.00-1500.00])...

10/13/2022 12:07 AM (4): Minora Feature Detector: F6: Found 364740 chromatographic peaks.

10/13/2022 12:07 AM (4): Minora Feature Detector: F5: Found 352599 chromatographic peaks.

10/13/2022 12:07 AM (4): Minora Feature Detector: F6: Importing auto traces...

10/13/2022 12:07 AM(4): Minora Feature Detector: F6: Imported 291815 auto traces.

10/13/2022 12:08 AM(4): Minora Feature Detector: F5: Importing auto traces...

10/13/2022 12:08 AM(4): Minora Feature Detector: F5: Imported 282650 auto traces.

10/13/2022 12:08 AM(4): Minora Feature Detector: Importing peak to spectrum links...

10/13/2022 12:10 AM(4): Minora Feature Detector: Found 13363 features (F5: 6496, F6: 6867)

10/13/2022 12:10 AM(4): Minora Feature Detector: Writing data took 9 s.

10/13/2022 12:10 AM(4): Minora Feature Detector: Creating features for PSMs took 17.9 s.

10/13/2022 12:11 AM(4): Minora Feature Detector: F5: Unidentified feature detection took 1 min 6 s

10/13/2022 12:11 AM(4): Minora Feature Detector: F6: Unidentified feature detection took 1 min 10 s

10/13/2022 12:12 AM(4): Minora Feature Detector: Found 141919 unidentified features (F5 : 69956, F6 : 71963).

10/13/2022 12:12 AM(4): Minora Feature Detector: -- Total execution of Minora Feature Detector (4) took 21 min 25 s --

10/13/2022 12:12 AM Job Execution: Finished V:\Chung Group\Livia\RawFiles\10_2022\Deivid\ALI_Oct2022\ALI_Oct2022\ALI_Oct2022.msf

10/13/2022 12:12 AM Job Execution: ----- Total Job execution took: 1 h 44 min. -----

==

Validation

==

==

Consensus Step Validation

==

Result name: ALI_Oct2022

Result file: V:\MED\ClinMed_Biomed\Restrict\Chung Group\Livia\RawFiles\2022\10_2022\Deivid\ALI_Oct2022\ALI_Oct2022\ALI_Oct2022.pdResult

Description: Result filtered for high confident peptides, with enhanced peptide and protein annotations. Add FASTA file with common contaminants to the Protein Marker node. Quan abunaces are normalized to the same total peptide amount per channel and scaled, so that the average abunce per protein and peptide is 100.

Workflow based on template: CWF_Comprehensive_Enhanced Annotation_LFQ_and_Precursor_Quan

Creation date: 10/12/2022 10:27:14 PM

Created with Discoverer version: 2.4.1.15

Peptide Validator nodes:

Processing node 2: Peptide Validator

----

1. General Validation Settings:

- Validation Mode: Automatic (Control peptide level error rate if possible)

- Target FDR (Strict) for PSMs: 0.01

- Target FDR (Relaxed) for PSMs: 0.05

- Target FDR (Strict) for Peptides: 0.01

- Target FDR (Relaxed) for Peptides: 0.05

2. Specific Validation Settings:

- Validation Based on: q-Value

- Target/Decoy Selection for PSM Level FDR Calculation Based on Score: Automatic

- Reset Confidences for Nodes without Decoy Search (Fixed score thresholds): False

Additional information:

Used validation mode: 'Automatic (Control peptide level error rate if possible)'.

All PSMs have PEPs. Qvality will be used for peptides.

Updated peptide confidences using qvality.

Protein Validator nodes:

Processing node 7: Protein FDR Validator

----

1. Confidence Thresholds:

- Target FDR (Strict): 0.01

- Target FDR (Relaxed): 0.05

==
==============

Processing Step A: Validation

==
==============

Result name: ALI_Oct2022

Result file: V:\Chung Group\Livia\RawFiles\10_2022\Deivid\ALI_Oct2022\ALI_Oct2022\ALI_Oct2022.msf

Description: Processing workflow for precursor-based quantification. CID spectra using SequestHT with Percolator validation. Specify the FASTA database, labels used, and any additional modifications.

Workflow based on template: PWF_OT_Precursor_Quan_and_LFQ_CID_SequestHT_Percolator

Creation date: 10/12/2022 10:27:12 PM

Created with Discoverer version: 2.4.1.15

Psm Validator nodes:

Processing node 3: Percolator

----

1. Target/Decoy Strategy:

- Target/Decoy Selection: Concatenated

- Validation based on: q-Value

2. Input Data:

- Maximum Delta Cn: 0.05

- Maximum Rank: 0

3. FDR Targets:

- Target FDR (Strict): 0.01

- Target FDR (Relaxed): 0.05

Validation for Processing Node: Sequest HT (2)

Percolator Output:

Results for Sequest HT (2):

Iteration 1:	Estimated 83266 PSMs with q<0.01

Iteration 2:	Estimated 83810 PSMs with q<0.01

Iteration 3: Estimated 83956 PSMs with q<0.01

Iteration 4: Estimated 83963 PSMs with q<0.01

Iteration 5: Estimated 83971 PSMs with q<0.01

Iteration 6: Estimated 83970 PSMs with q<0.01

Iteration 7: Estimated 83968 PSMs with q<0.01

Iteration 8: Estimated 83967 PSMs with q<0.01

Iteration 9: Estimated 83972 PSMs with q<0.01

Iteration 10: Estimated 83977 PSMs with q<0.01

Learned normalized SVM weights for the 3 cross-validation splits:

Split1	Split2	Split3	FeatureName
0.4830	0.4659	0.4078	XCorr
0.0618	0.0829	0.0723	Delta Cn From Second PSM
2.3140	2.1834	2.4532	Binomial Score
0.1130	0.0760	0.1424	Isolation Interference [%]
0.1928	0.3107	0.3705	MH+ [Da]
0.3533	0.2419	0.1039	Delta Mass [Da]
-0.4922	-0.3717	-0.2214	Delta Mass [ppm]
0.2299	0.0284	0.2424	Absolute Delta Mass [Da]
-1.4769	-1.2884	-1.4878	Absolute Delta Mass [ppm]
-0.1687	-0.3000	-0.3585	Peptide Length
0.0000	0.0000	0.0000	Is z=1
-0.0593	0.0958	0.1767	Is z=2
0.1044	0.0942	0.0910	Is z=3

0.0036	-0.1854	-0.2701	Is z=4
-0.0688	-0.1483	-0.2337	Is z=5
-0.1656	-0.2795	-0.3320	Is z>5
-0.2976	-0.2767	-0.3237	# Missed Cleavages
-0.0008	-0.0133	-0.0098	Log Peptides Matched
-0.1999	-0.2342	-0.2867	Log Total Intensity
0.3568	0.3272	0.3375	Fraction Matched Intensity [%]
-0.4963	-0.5741	-0.6159	Fragment Coverage Series A, B, C [%]
-0.4166	-0.4313	-0.4209	Fragment Coverage Series X, Y, Z [%]
-1.1780	-0.9453	-0.9005	Log Matched Fragment Series Intensities A, B, C
1.3073	1.3279	1.5062	Log Matched Fragment Series Intensities X, Y, Z
0.2019	0.2620	0.2318	Longest Sequence Series A, B, C
0.8225	0.7702	0.8309	Longest Sequence Series X, Y, Z
-0.1186	0.0240	-0.0228	IQR Fragment Delta Mass [Da]
-0.1612	-0.2504	-0.1129	IQR Fragment Delta Mass [ppm]
-0.5338	-0.5393	-0.6068	Mean Fragment Delta Mass [Da]
0.1484	0.1229	0.1805	Mean Fragment Delta Mass [ppm]
-0.4532	-0.5200	-0.5763	Mean Absolute Fragment Delta Mass [Da]
-0.0774	-0.0671	-0.1176	Mean Absolute Fragment Delta Mass [ppm]
0.7006	0.2864	0.7235	m0

==
=================

Filters and Counts

==
=================

Applied display filters:

This file contains the following filters:

Row Filter for Proteins:

AND

|

+--Master is equal to Master

|

+--in category

  |

  +--Protein FDR Confidence

  |

  +--1

Row Filter for Input Files:

FileName does not end with msf

Number of result items:

Proteins:

1540 filtered / 4150 included / 26049 total

Protein Groups:

1693 included / 1704 total

Peptide Groups:

8998 included / 34434 total

PSMs:

82025 included / 120745 total

MS/MS Spectrum Info:

156022 total

Input Files:

6 filtered / 7 included / 7 total

Specialized Traces:

18 total

Consensus Features:

126868 total

Result Statistics:

251 total

==
==============

FDR Values for Entire Result

==
==============

Actual estimated FDR values (without applied display filters):

These estimated FDR values are based on simple counting of target and decoy items

and may divert from the target FDR values you have set in the validation nodes

used during workflow processing.

Usually the values will be slightly more conservative than using validation

based on linear discriminant analysis or other sophisticated methods.

High confident results:

0.003 (82025 targets, 212 decoys) for Peptide-Spectrum Matches

0.010 (8998 targets, 89 decoys) for Peptide Groups

0.005 (3755 targets, 20 decoys) for Proteins

(No confidence was assigned to Protein Groups)

Medium and High confident results:

0.003 (82025 targets, 212 decoys) for Peptide-Spectrum Matches

0.010 (8998 targets, 89 decoys) for Peptide Groups

0.045 (4150 targets, 187 decoys) for Proteins

(No confidence was assigned to Protein Groups)

Whole dataset:

0.003 (82025 targets, 212 decoys) for Peptide-Spectrum Matches

0.010 (8998 targets, 89 decoys) for Peptide Groups

0.045 (4150 targets, 188 decoys) for Proteins

0.053 (1693 targets, 89 decoys) for Protein Groups

==
==================================

Quantification

==
==================================

==
==============

Consensus Step Quantification

==
==============

Result name: ALI_Oct2022

Result file: V:\MED\ClinMed_Biomed\Restrict\Chung Group\Livia\RawFiles\2022\10_2022\Deivid\ALI_Oct2022\ALI_Oct2022\ALI_Oct2022.pdResult

Description: Result filtered for high confident peptides, with enhanced peptide and protein annotations. Add FASTA file with common contaminants to the Protein Marker node. Quan abunaces are normalized to the same total peptide amount per channel and scaled, so that the average abunce per protein and peptide is 100.

Workflow based on template: CWF_Comprehensive_Enhanced Annotation_LFQ_and_Precursor_Quan

Creation date: 10/12/2022 10:27:14 PM

Created with Discoverer version: 2.4.1.15

Processing node 11: Precursor Ions Quantifier

----

1. General Quantification Settings:

- Peptides to Use: Unique + Razor

- Consider Protein Groups for Peptide Uniqueness: True

- Use Shared Quan Results: True

- Reject Quan Results with Missing Channels: False

2. Precursor Quantification:

- Precursor Abundance Based On: Intensity

- Min. # Replicate Features [%]: 0

3. Normalization and Scaling:

- Normalization Mode: Total Peptide Amount

- Scaling Mode: None

4. Exclude Peptides from Protein Quantification:

- For Normalization: Use All Peptides

- For Protein Roll-Up: Use All Peptides

- For Pairwise Ratios: Exclude Modified

5. Quan Rollup and Hypothesis Testing:

- Protein Abundance Calculation: Summed Abundances

- N for Top N: 3

- Protein Ratio Calculation: Pairwise Ratio Based

- Maximum Allowed Fold Change: 100

- Imputation Mode: None

- Hypothesis Test: t-test (Background Based)

6. Quan Ratio Distributions:

- 1st Fold Change Threshold: 2

- 2nd Fold Change Threshold: 4

- 3rd Fold Change Threshold: 6

- 4th Fold Change Threshold: 8

- 5th Fold Change Threshold: 10

==

Processing Step A: Quantification

==

Result name: ALI_Oct2022

Result file: V:\Chung Group\Livia\RawFiles\10_2022\Deivid\ALI_Oct2022\ALI_Oct2022\ALI_Oct2022.msf

Description: Processing workflow for precursor-based quantification. CID spectra using SequestHT with Percolator validation. Specify the FASTA database, labels used, and any additional modifications.

Workflow based on template:
PWF_OT_Precursor_Quan_and_LFQ_CID_SequestHT_Percolator

Creation date: 10/12/2022 10:27:12 PM

Created with Discoverer version: 2.4.1.15

Processing node 4: Minora Feature Detector

----

1. Peak & Feature Detection:

- Min. Trace Length: 5

- Max. ΔRT of Isotope Pattern Multiplets [min]: 0.2

2. Feature to ID Linking:

- PSM Confidence At Least: High

==

Derived Quantification Values

==

Normalization/ Scaling:

Normalization Mode = Total Peptide Amount

Protein For Normalization = No FASTA File Selected

Scaling Mode = None

Applied Normalization Values:

1.435: F4: Sample, 2D

1.127: F5: Sample, 2D

1.000: F6: Sample, 2D

1.438: F1: Sample, ALI

1.390: F2: Sample, ALI

1.466: F3: Sample, ALI

==

==============

Ratios and Ratio Groups

==

==============

Quantification Group Settings:

Study variable(s) for grouping: TYPE

Groups sorted by: TYPE (SortAscending)

Quantification Ratios:

(ALI) / (2D)

Quantification Ratio Coloring:

Normal space ratio threshold (log2 ratio threshold)

blue (1): < 0.1 (< -3.3)

blue (2): < 0.125 (< -3)

blue (3): < 0.167 (< -2.6)

blue (4): < 0.25 (< -2)

blue (5): < 0.5 (< -1)

white: between 0.5 and 2 (between -1 and 1)

red (5): > 2 (> 1)

red (4): > 4 (> 2)

red (3): > 6 (> 2.6)

red (2): > 8 (> 3)

red (1): > 10 (> 3.3)

==
=======================================

Configuration

==
=======================================

==
================

Consensus Workflow Configuration

==
================

Result name: ALI_Oct2022

Result file: V:\MED\ClinMed_Biomed\Restrict\Chung Group\Livia\RawFiles\2022\10_2022\Deivid\ALI_Oct2022\ALI_Oct2022\ALI_Oct2022.pdResult

Description: Result filtered for high confident peptides, with enhanced peptide and protein annotations. Add FASTA file with common contaminants to the Protein Marker node. Quan abunaces are normalized to the same total peptide amount per channel and scaled, so that the average abunce per protein and peptide is 100.

Workflow based on template: CWF_Comprehensive_Enhanced Annotation_LFQ_and_Precursor_Quan

Creation date: 10/12/2022 10:27:14 PM

Created with Discoverer version: 2.4.1.15

Configuration for: MSF Files

Scores:

- PSM scores (Hidden):

Mascot: Ions Score

Sequest HT: XCorr

SEQUEST: XCorr

MSPepSearch: dot Score

MSPepSearch: rev-dot Score

MSPepSearch: MSPepSearch Score

PMI-Byonic: |Log Prob|

PMI-Byonic: Byonic Score

MS Amanda: Amanda Score

Configuration for: Protein Scorer

Configuration Settings for Protein Score 'SequestSummationScore':

Protein Scoring Options:

- Peptide Relevance Factor: 0.4

Configuration for: Display Settings

Default Display Filter:

- Default Filter Set (Hidden):

Master Proteins Default Filter:

Row Filter for TargetProtein:

Master is equal to Master

###

'magellan filter set' 1 'MasterFilter.filterset' Filter 'TargetProtein' FilterProperties 1 'FilterConditionProperties/FilterScope' 'FilterConditionProperties/FilterScopeValueMainGrid' 1 NARY_AND 1 = FilterConditionProperties 1 'NamedComparableFilterCondition/DisplayPropertyHint' 'Master' property 'Thermo.PD.EntityDataFramework.MasterProteinAssessment, Thermo.Magellan.EntityDataFramework' 'IsMasterProtein' constant 'Thermo.PD.EntityDataFramework.MasterProteinAssessment, Thermo.Magellan.EntityDataFramework' 'IsMasterProtein'

Default Layout:

- Default Layout: (not specified)

==
==============

Processing Workflow A: Configuration

==
==============

Result name: ALI_Oct2022

Result file: V:\Chung Group\Livia\RawFiles\10_2022\Deivid\ALI_Oct2022\ALI_Oct2022\ALI_Oct2022.msf

Description: Processing workflow for precursor-based quantification. CID spectra using SequestHT with Percolator validation. Specify the FASTA database, labels used, and any additional modifications.

Workflow based on template: PWF_OT_Precursor_Quan_and_LFQ_CID_SequestHT_Percolator

Creation date: 10/12/2022 10:27:12 PM

Created with Discoverer version: 2.4.1.15

Configuration for: Spectrum Files RC

Search settings:

- Ion Series settings (Hidden):

CID: by

HCD: by

ECD: cyz

ETD: cyz

EThcD: bcyz

UVPD: abxyz

Configuration for: Sequest HT

----

1. Workload Level:

- Automatic: True

- Number of Spectra Processed At Once: 3000

- Number of Parallel Tasks: 0

2. XCorr Confidence Thresholds (low-resolution data):

- z=1: High Confidence XCorr: 1.5

- z=1: Medium Confidence XCorr: 0.7

- z=2: High Confidence XCorr: 2

- z=2: Medium Confidence XCorr: 0.9

- z=3: High Confidence XCorr: 2.5

- z=3: Medium Confidence XCorr: 1.2

- z>=4: High Confidence XCorr: 3

- z>=4: Medium Confidence XCorr: 1.5

3. XCorr Confidence Thresholds (high-resolution data):

- z=1: High Confidence XCorr: 1.2

- z=1: Medium Confidence XCorr: 0.7

- z=2: High Confidence XCorr: 1.9

- z=2: Medium Confidence XCorr: 0.8

- z=3: High Confidence XCorr: 2.3

- z=3: Medium Confidence XCorr: 1

- z>=4: High Confidence XCorr: 2.6

- z>=4: Medium Confidence XCorr: 1.2

Configuration for: Minora Feature Detector

Configuration Settings:

- Number of Parallel Tasks: 0
